# *Ganoderma lucidum* spore powder enhances IFN-α-mediated antiviral capacity of COVID-19 vaccine boosters revealed by single-cell multi-omics sequencing

**DOI:** 10.1016/j.jare.2025.10.014

**Published:** 2025-10-12

**Authors:** Anyao Li, Xiaoyan Lu, Rongfang Guo, Wenbo Guo, Penghui Yang, He Lou, Jie Chen, Enshan Huang, Ronghua Zhang, Hanbo Wang, Jihong Yang, Zhenhao Li, Xiaohui Fan

**Affiliations:** a College of Pharmaceutical Sciences, Zhejiang University, Hangzhou 310058, China; b State Key Laboratory of Chinese Medicine Modernization, Innovation Center of Yangtze River Delta, Zhejiang University, Jiaxing, Zhejiang, China; c The Joint-laboratory of Clinical Multi-Omics Research Between Zhejiang University and Ningbo Municipal Hospital of TCM, Ningbo Municipal Hospital of TCM, Ningbo 315100, China; d ShouXianGu Botanical Drug Institute, Hangzhou 311121 Zhejiang, China; e Zhejiang Key Laboratory of Biological Breeding and Exploitation of Edible and Medicinal Mushrooms, Wuyi 321200 Zhejiang, China; f Zhejiang Provincial Center for Disease Control and Prevention, Hangzhou, Zhejiang 310051, China

**Keywords:** *Ganoderma lucidum* spore powder, Adjuvant, COVID-19 vaccine, Single-cell, IFN-α

## Abstract

**Introduction:**

Reduced vaccine efficacy may hinder stable herd immunity. Traditional natural medicinal ingredients, such as *Ganoderma lucidum* products, have shown potential as safe and effective adjuvants to improve vaccine efficacy. However, their roles in assisting human epidemiological vaccines remain uninvestigated.

**Objectives:**

This study aimed to evaluate *Ganoderma lucidum* spore powder (GLSP) as an adjuvant for coronavirus disease 2019 (COVID-19) vaccine boosters and to elucidate its underlying immunomodulatory mechanisms.

**Methods:**

The microneutralization assay was utilized to measure the percentage of neutralizing antibodies (NAbs) inhibition. The single-cell multi-omics sequencing was performed to investigate the mechanism of GLSP. The ultra-performance liquid chromatography quadrupole time-of-flight mass spectrometry (UPLC-QTOF-MS) and ultra-performance liquid chromatography multiple reaction monitoring mass spectrometry (UPLC-MRM-MS) were used to characterize the chemical composition of GLSP. The molecular docking and in vitro validated experiments were performed to identify triterpenoids that are active in inducing interferon (IFN)-α production and signaling.

**Results:**

In comparison with vaccination alone, GLSP significantly enhanced the NAbs percent inhibition in participants with moderate baseline immunity at 90 days post-vaccination. At the cellular level, GLSP substantially expanded the myeloid cell proportions, particularly classical monocytes (CMs), and augmented their interactions with T and B cells. It also promoted a stronger IFN-α response in innate and adaptive immune cells. In innate immunity, GLSP enhanced the transcription factor activity and chromatin accessibility related to IFN-α stimulation. In adaptive immunity, GLSP induced distinct biases for gene usage and clonal expansion of BCRs and TCRs, contributing to stronger antiviral immunity. Crucially, a combination of chemical profiling and functional validation identified the triterpenoid ganoderic acid H (GH) as the key active component responsible for inducing the IFN-α response.

**Conclusions:**

GLSP demonstrates significant potential as a safe, orally administered adjuvant to counteract waning vaccine efficacy by promoting a robust, IFN-α-mediated antiviral state at the multi-omics level.

## Highlights

• GLSP increased vaccine efficacy in individuals with moderate immunity.
• GLSP improved myeloid proportions and interactions with adaptive immune cells.
• GSLP influenced the upstream regulation to enhance the IFN response.
• GLSP induced biases of BCR and TCR for stronger resistance to virus.
• GLSP enhanced IFN-α-mediated antiviral ability in both innate and adaptive immunity.

## Graphical abstract

**Figure:**
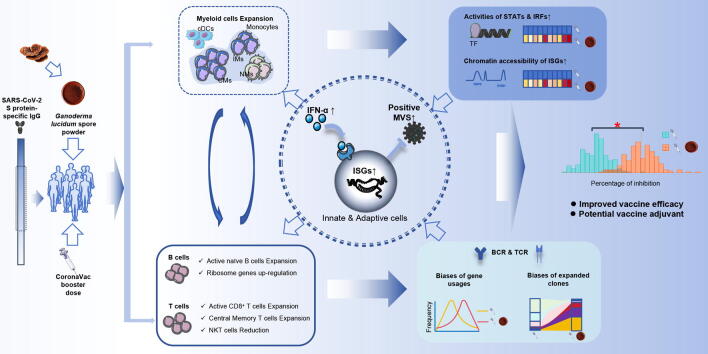

## Introduction

Coronavirus disease 2019 (COVID-19) poses a major threat to the lives and property of people around the world, with more than 770 million cases and 7 million deaths as of September 2024 (https://covid19.who.int). Large-scale epidemics that claim thousands of lives and cause significant economic losses have occurred throughout human history [1,2]. Due to factors such as increased urbanization and inter-regional travel, pandemics are still possible in the future [2]. Vaccine development and mass injections are key to preventing and controlling outbreaks and reducing mortality [3]. Currently, multiple vaccines have been approved and are being administered globally. Studies suggest that a 10 % increase in COVID-19 vaccination coverage is associated with a 1.3 %–1.7 % reduction in new cases, a 5 % reduction in hospitalizations, a 12 % reduction in ICU admissions, and a 2 % reduction in deaths [4]. A common problem with most vaccines, however, is waning efficacy. The mRNA-1273, BNT162b2, and Ad26.COV2.S vaccines each experienced at least a 20 % decrease in effectiveness five months after injection compared to 14 days post-injection [5]. Similarly, the effectiveness of Vaxzevria and Comirnaty against symptomatic disease declined significantly five months after the second dose, while the vaccine’s effectiveness in preventing hospitalization and death declined to a lesser extent [6]. Therefore, vaccine booster shots are becoming increasingly necessary [7,8].

The development of vaccine adjuvants is an alternative strategy to achieve a more rapid, stronger, longer-lasting immune response. Several immune adjuvants have been developed and used in the clinic, both intravenously and orally [9,10]. Among them, common adjuvants such as aluminum hydroxide, MF59, Matrix-M, and AS03 have been used in the development of COVID-19 vaccines and have achieved good effectiveness [[11], [12], [13], [14], [15]]. As their use becomes more intensive and widespread, potential safety concerns and limitations of action of these vaccine adjuvants are being raised. For example, the H1N1 vaccine using AS03 as an adjuvant has been reported to be associated with an increased incidence of episodic sleeping sickness in children [16,17]. The effects of MF59 are thought to be limited to the injection site and draining lymph nodes [18], and vaccines adjuvanted with MF59 are more likely to cause a local or systemic reaction than non-adjuvanted vaccines. Aluminum hydroxide is the first and most used vaccine adjuvant [9,19], which can activate innate immune reactions and promote Th2 responses, but it provides limited protection against diseases that rely on Th1- and major histocompatibility complex (MHC)-class-I-restricted cytotoxic T lymphocyte responses [20]. Despite the rarity of adverse events, concerns continue to grow due to the neurological toxicity of aluminum salts and the potential to trigger allergies [21,22]. Based on the aforementioned considerations, it is imperative to develop adjuvants that can systematically activate the immune response while ensuring safety and stability.

Active ingredients of traditional Chinese medicine (TCM) herb extracts may fulfill this requirement, and their feasibility as vaccine adjuvants is increasingly being explored and validated [[23], [24], [25]]. *Ganoderma lucidum (GL)* is a traditional medicinal mushroom with a high medicinal value, and its compounds are excellent at inducing active cellular and humoral immune responses. By promoting the maturation of dendritic cells, which in turn enhances mixed lymphocyte responses and selectively increases cytokine levels, GL components can serve as effective adjuvants for vaccines targeting tumors, tetanus, and other diseases [[26], [27], [28]]. Oral polysaccharides derived from GL represent a promising adjuvant choice, having been evaluated in chickens for their synergistic effects with the Newcastle disease vaccine ConA, where they promote lymphocyte proliferation and enhance serum antibody potency [28]. However, few reports exist on its usefulness as an adjuvant for human epidemiological vaccines. On the other hand, the active ingredients of GL are predominantly concentrated in its spores, and several studies have reported that the administration of GL spores (GLSP) can induce anti-tumor effects through immune modulation [29,30]. In addition, administration of GLSP improved estradiol benzoate (EB) −induced thymic atrophy and regulated T-cell development in mice [31]. Given these findings, it is reasonable to assume that GLSP could serve as an immune adjuvant and supplement, potentially enhancing the immune response of the body to vaccines against infectious diseases.

Single-cell multi-omics sequencing paves the way for a multidimensional understanding of disease progression and the mechanisms underlying drug interventions, playing a crucial role in elucidating immune responses to SARS-CoV-2 infection and vaccination. Aligning with the recent consensus to integrate modern technologies like artificial intelligence with traditional medicine [32], we employed a comprehensive approach to utilize single-cell multi-omics sequencing to elucidate the mechanism of action of GLSP. The single-cell RNA sequencing has enabled the identification of cell states and humoral immune responses associated with infection and vaccination [[33], [34], [35]]. By combining single-cell RNA sequencing with V(D)J sequencing, researchers have effectively tracked the dynamics of both specifically and non-specifically expanded T and B cells [[33], [34], [35]]. Furthermore, multi-omics integrated analysis at the single-cell level has greatly enhanced our understanding of epigenetic alterations and changes in surface protein levels through longitudinal and cross-sectional comparisons [[36], [37], [38], [39]]. In conclusion, single-cell multi-omics sequencing offers a comprehensive perspective of the immune system.

In this study, we aimed to demonstrate the potential of GLSP as a vaccine adjuvant for the SARS-CoV-2 booster vaccine. To this end, we employed a comprehensive approach, elucidating the mechanism of action of GLSP through single-cell transcriptomics, V(D)J sequencing, and assay for transposase-accessible chromatin (ATAC) sequencing. All 195 volunteers received a booster dose of the SARS-CoV-2 vaccine. Half of them received GLSP synchronously by oral administration, while the remainder served as a blank control group. The effects of booster shots and GLSP on antibody titers were tracked, and the effective population and the time point of significant onset of GLSP action were identified. Single-cell multi-omics sequencing identified the effects of GLSP on innate and adaptive immunity. GLSP promoted the overall expansion of myeloid cell subsets, as well as active naïve B cells and *CD8*^+^ T cells, and improved their interactions. The GLSP intervention group exhibited distinct gene usage and clonal preferences for B-cell and T-cell receptors compared with the control group. Moreover, GLSP significantly elevated serum interferon (IFN)-α levels and augmented the type I IFN response through its influence on transcription, V(D)J rearrangement, and epigenetic regulation. In conclusion, by enhancing IFN-α-mediated antiviral capacity, GLSP improves the efficacy of COVID-19 vaccine booster shots.

## Materials and methods

### Study design

Human peripheral blood samples used in this study were collected and provided by the Zhejiang Provincial Center for Disease Control and Prevention. The 195 volunteers, aged between 20 and 60, had received two doses of the COVID-19 vaccine at least six months prior and had no history of vaccine allergy or autoimmune disease. All volunteers were randomly divided into a control group and a GLSP intervention group, and both groups received a booster shot of the COVID-19 vaccine (*CoronaVac*), produced by Sinovac Biotech Co., Ltd. Among them, the control group used a blank control, and the GLSP intervention group took 2 g of GLSP twice a day for six months. GLSP used in this study was provided by Zhejiang ShouXianGu Pharmaceutical Co., Ltd, whose product name was sporoderm-removed *Ganoderma lucidum* spore powder (batch No. 2106221). The chemical compositions of GLSP were detailed in the product document. All volunteers signed an informed consent before the study commenced. The Ethics Review Committee of the Zhejiang Provincial Center for Disease Control and Prevention approved the study protocol and informed consent. The clinical registration number is ChiCTR2300078082, and the ethical approval number is 2021-033-01. The general information of all volunteers is presented in Supplementary Table S1.

### Neutralizing antibody (NAb) measurement

The neutralizing antibody titer was determined by the microneutralization assay. First, the serum was diluted in a series of steps from 1:2 to 1:256. Then, the virus was diluted to 200 TCID50/0.1 mL. The diluted virus was mixed with the diluted serum in equal volumes, resulting in a final serum dilution ranging from 1:4 to 1:512, while the virus titer was maintained at 100 TCID50/0.1 mL. This mixture was incubated at 37 °C for 2 h to allow for viral neutralization. Subsequently, the virus-serum mixture was inoculated onto a monolayer of E6 cells and cultured at 37 °C for 3–5 days. The cytopathic effect (CPE) was then assessed to determine the NAbs titer and the percentage of inhibition. The statistics on the percentage of inhibition under different conditions are presented in Supplementary Table S2.

### Detection of serum SARS-CoV-2 S protein-specific IgG level

A SARS-CoV-2 Spike Protein IgG Enzyme-linked Immunosorbent Assay (ELISA) Kit (Elabscience, Cat# E-EL-E602) was used to quantify the levels of SARS-CoV-2 S protein-specific IgG in pre-injection serum samples from all volunteers. Each serum sample was diluted five times before the assay. The optical density (OD) value at 450 nm wavelength was measured using a SpectraMax M5 Multi-Mode Microplate Reader.

### Collection of peripheral blood mononuclear cells (PBMC)

First, whole blood samples, phosphate-buffered saline (PBS, Servicebio, Cat# G4202-500 mL), and Ficoll density gradient medium (Ficoll® Paque Plus, MilliporeSigma, Cat# GE17-1440–02) were mixed in a 1:1:1 ratio. This mixture was then subjected to centrifugation at 400 g for 30 min at room temperature. The PBMC layer was collected, washed with Dulbecco’s PBS (DPBS, Servicebio, Cat# G4200-500 mL), and centrifuged at room temperature at 250 g for 10 min. Subsequently, 1–2 mL of red blood cell lysis buffer (Beyotime, Cat# C3702-120 mL) was added, and the contents were shaken for 10 min in a dark environment. After the completion of lysis, the sample should be washed twice with DPBS. The collected PBMCs were resuspended in foetal bovine serum (FBS, Gibco, Cat# 10099141C) containing 10 % dimethyl sulfoxide (DMSO, Sinopharm Chemical Reagent, Cat# 30072418) and then frozen at -80 °C for later use.

### The single-cell multi-omics library preparation and sequencing

The frozen PBMC samples prepared for the single-cell library were thawed at 37 °C. Subsequently, Dulbecco’s Modified Eagle Medium (DMEM, Gibco, Cat# C11995500CP) was added, and the suspension was centrifuged at 1000 rpm for 4 min at 4 °C. After removing the supernatant, the cells were suspended in PBS containing 0.4 % bovine serum albumin (BSA, Beyotime, Cat# ST023-200g) and stained with trypan blue for counting. For samples with less than 80 % live cells, dead cells were removed with a Dead Cell Removal Kit (Miltenyi Biotec, Cat# 130-090-101), then the samples were resuspended and a second counting was performed. Here, we selected PBMC samples from 12 individuals for single-cell transcriptomics and V(D)J sequencing, and then randomly assigned half of these samples for ATAC sequencing (Supplementary Table S3).

According to the operation instructions of Chromium Next GEM Single Cell 5′ Kit v2 of 10 × Genomics, the transcriptome and immune profile libraries were constructed. The previously prepared cell suspension was added to the chip of the Chromium Controller, a single-cell library automated sample preparation system, to generate a large number of water-in-oil droplets (GEMs). These droplets are then coated with single cells and a barcoded gel bead carrying cell barcode sequences. The complementary DNA (cDNA) was generated by reverse transcription in GEMs and subsequently purified and amplified. The constructed libraries were sequenced using the NovaSeq PE150 sequencing strategy.

According to the operation instructions of Chromium Next GEM Single Cell ATAC Reagent Kit v1.1 of 10 × Genomics, a single-cell ATAC library was constructed. Specifically, the cell suspension was first used to isolate nuclei according to the 10 × Genomics PBMC sample extraction protocol (CG000169 • Rev E). Then, the nuclear suspension was treated with the Tn5 enzyme so that the sequencing splice could be inserted into the open chromatin region. In the chip of the Chromium Controller, a single nucleus, the Tn5 enzyme, and a barcoded gel bead formed the GEMs. Finally, GEMs were collected, purified, and amplified. The resulting libraries were sequenced using the NovaSeq PE50 strategy.

### Analysis of single-cell transcriptome sequencing (scRNA-seq) data

Cell Ranger (7.1.0) was used to perform sequence alignment, quantification, and count matrix generation of the sequencing data, and the human genome GRCh38 was used as the reference genome. Genes expressed in fewer than 10 cells were excluded from the analysis, as well as cells with fewer than 200 or more than 4,000 reads. Moreover, cells where more than 10 percent of the reads corresponded to mitochondrial genes were also filtered out. Doublets identified using the DoubletFinder tool (version 2.0.3) [40] were removed from the dataset. The Seurat tool (4.3.0.1) [41] was used for dimension reduction, clustering, and downstream analysis. The *NormalizeData* function was employed for standardization, the *FindVariableFeatures* function was used for high-variability feature extraction, the *ScaleData* function was used to effect a linear transformation of the data, and the *RunPCA* function was applied for principal component analysis (PCA). Subsequently, the Harmony (0.1.1) tool [42] was used to correct batch effects between samples. The first 35 principal components, after correction, were used for subsequent K-nearest neighbor (KNN) graph construction, clustering, and uniform manifold approximation and projection (UMAP) dimension reduction. Then, we annotated the cell type of each cluster using known markers. The odds ratio (OR), calculated by Fisher’s exact test, measures the bias of the distribution of cell types or clusters in different groups.

### Enrichment analysis

We used an online platform (https://metascape.org), Metascape [43], and performed enrichment analyses using databases such as Gene Ontology (GO), Kyoto Encyclopedia of Genes and Genomes (KEGG), Reactome, and MSigDB.

### The gene set selection and scoring

The gene score was calculated using Seurat’s *AddModuleScore* function. The genes associated with IFN-α and IFN-γ responses were sourced from the HALLMARK entries “INTERFERON ALPHA RESPONSE” and “INTERFERON GAMMA RESPONSE” of the GSEA database [44]. Meanwhile, the genes related to the JAK-STAT6 signaling pathway were obtained from the “JAK-STAT6 signaling” entry in the KEGG database. The meta-viral signature (MVS) [45] was derived from conserved transcriptional signatures of patients with respiratory virus infections through comprehensive multi-cohort analysis, including genes with positive and negative effects. We classified the genes exhibiting both positive “Discovery Summary Effect Size” and positive “Validation Summary Effect Size” as positive MVS gene sets. Conversely, genes with both negative “Discovery Summary Effect Size” and negative “Validation Summary Effect Size” were categorized as negative MVS gene sets. The cytotoxicity gene set consists of the following genes: *PRF1, IFNG, GNLY, NKG7, GZMB, GZMA, GZMH, KLRK1, KLRB1, KLRD1, CTSW, and CST7*. Features of central memory T (Tcm) signatures include *CCR7, TCF7, SELL, LEF1, BCL2, CD27, and IL7R*. The exhaustion gene set comprises *LAG3, TIGIT, PDCD1, CTLA4, HAVCR2,* and *TOX*.

### Analysis of transcription factor (TF) regulatory network

The single-cell regulatory network inference and clustering (pySCENIC 0.12.1) tool [[46], [47]] was utilized to infer TF regulatory networks and assess the regulatory activity of TF-targets composition (regulon), based on scRNA-seq data. Before analysis, the count matrix was preprocessed to remove genes that were expressed in fewer than 1000 cells. The limma tool [48] was used to calculate the differential activity of regulons between the control and the GLSP intervention groups. Regulons with log fold change (logFC) more than 0.1 and adjusted *p*-value (adj.Pval) less than 0.05 were considered to have significant activity differences between groups.

### RNA velocity analysis

RNA velocity analysis was performed using velocyto (0.17.16) [49] and scVelo (0.3.1) [50]. We used filtered BAM files as input for velocyto to calculate spliced and unspliced reads for each sample. Afterward, RNA velocity was inferred and visualized using the scVelo dynamic model. Specifically, the *scv.pp.filter_and_normalize* and *scv.pp.moments* functions were used to pre-process and compute the first and second-order moments among nearest neighbors in Harmony space. The *scv.tl**.recover_dynamics* and *scv.tl**.velocity* (mode = “dynamical”) functions were used to estimate the velocity in the dynamic model. The *scv.tl**.velocity_graph* function was utilized to project the velocities into a lower-dimensional embedding. The *scv.tl**.latent_time* function was applied to recover the latent time underlying cellular processes.

### Cell-cell interaction (CCI) analysis

CCI analysis was performed using the CellChat (2.1.2) tool [51]. We inferred communication between sub-clusters of all cell types following the standard workflow of CellChat under different conditions, respectively. Furthermore, we used the comparison analysis strategy of the tool to identify the differences in cellular interactions between the pre- and post-injection and between the control and GLSP intervention groups.

### Detection of IFN-α level

We used the Human IFN-α (Interferon Alpha) ELISA Kit (Elabscience, Cat# E-EL-H6125) to detect the serum IFN-α concentrations of control and drug intervention groups 90 days post-injection. The serum samples within the same group were mixed, and six replicate measurements were performed. The cell supernatant samples were diluted 50 times before testing. According to the kit instructions, the OD value at 450 nm wavelength was read using the SpectraMax M5 Multi-Mode Microplate Reader.

### Analysis of single-cell V(D)J sequencing (scVDJ-seq) data

Cell Ranger (7.1.0) was used for sequence alignment and assembly of single-cell BCR and TCR data, and the reference genome was GRCh38. After that, the Dandelion (0.3.5) tool [52] was used for the following analysis. In the Singularity container, *dandelion-preprocess* was utilized to carry out the preprocessing step. The *filter_contigs* function was employed to link transcriptome and immune profile information within the same cell based on matching barcodes. The *tl.transfer* function was used to integrate the data. The *tl.find_clones* function was used for clonotype calling, and the default parameters were set to identify clones with identical V and J genes, with a maximum of 15 % mismatch allowed in the complementarity-determining region 3 (CDR3) sequences.

### The TCR-peptide binding prediction by MixTCRpred

MixTCRpred [53] is a machine-learning predictor of TCR-peptide binding. According to the requirements of MixTCRpred, the input data, including gene names and CDR3 sequences of TCR α and β chains, were sorted to predict the binding ability of 26 SARS-CoV-2-related peptide epitopes recorded in the MixTCRpred database. A strong TCR-peptide binding was defined by a *perc_rank* value of less than 0.5, following the recommendations provided by the authors of MixTCRpred.

### Analysis of single-cell ATAC sequencing (scATAC-seq) data

Cell Ranger-atac (2.1.0) was used for sequence alignment of scATAC-seq, and the reference genome was GRCh38. Follow-up analysis was performed using ArchR (1.0.3) [54]. The *createArrowFiles* function was applied to build the Arrow files and ArchR projects. Nuclei with a TSS enrichment score of less than 10 or with fewer than 1 × 10^3.5^ unique nuclear fragments were filtered out. In addition, double peaks were calculated using the *addDoubletScores* function, and nuclei with double peaks were filtered through the *filterDoublets* function. Then, dimensionality reduction of the ATAC data was performed with the *addIterativeLSI* function, followed by batch effect correction using the *addHarmony* function. Clustering was carried out with the *addClusters* function, and a 2D visualization was generated using *the addUMAP* function. The *addGeneScoreMatrix* function was employed to calculate gene scores by integrating accessibility across the entire gene bodies while minimizing the influence of unrelated regulatory elements. The *addImputeWeights* function was used to compute imputation weights by smoothing signals between nearby cells. The *addModuleScore* function was used to calculate the overall score for the “IFN-α response” term genes.

### Chemical profiling and identification

Fifty milligrams of GLSP were ultrasonically extracted with 20 mL of 90 % methanol for 30 min, then centrifuged at 4000 r/min for 10 min. The supernatant was collected as a sample solution.

The sample solution was analyzed using the ultra-performance liquid chromatography quadrupole time-of-flight mass spectrometry (UPLC-QTOF-MS). Here, ACQUITY UPLC I-Class Plus (Waters Corporation, USA) and SYNAPT XS high-resolution mass spectrometer (Waters Corporation, USA) were used. The chromatographic column was Waters ACQUITY UPLC HSS T3 (2.1 mm × 100 mm, 1.8 μm). Mobile phase A was a 0.1 % formic acid aqueous solution; mobile phase B was acetonitrile. The gradient elution conditions were as follows: 0 min, 20 % B; 0–2 min, 20 %-26.5 % B; 2–9 min, 26.5 % B; 9–19 min, 26.5 %-35 % B; 19–28 min, 35 %-60 % B; 28–32 min, 60 %-70 % B; 32-37 min, 70 %-90 % B; 37–40 min, 90 %-100 % B; 40–45 min, 100 % B. The parameters were set: flow rate of 0.45 mL/min, column temperature of 25 °C, and injection volume of 1 μL. The mass spectrometer was operated in negative electrospray ionization (ESI) mode. The parameters were set: the ion source temperature was 140 °C; the nebulizer pressure was 6.0 bar; the cone voltage was 35 V; the collision energy was 25–50 eV; the desolvation gas temperature was 450 °C; the desolvation gas flow rate was 1000 L/h; the capillary voltage was 2.5 kV; the acquisition mode was MSE with a scanning range of *m*/*z* from 50 to 1200.

Mass spectrometry data were analyzed using Progenesis QI software (v2.4). The data were uploaded to the Global Natural Products Social (GNPS) platform, and a mass spectrometry molecular network (MSMN) was established for identification. The mass error for precursor ion mass tolerance and fragment ion mass tolerance was set to 0.02 Da, the cosine fraction threshold was set to 0.7, the minimum matching fragment ion was 4, and default values were selected for the remaining parameters. The mass spectrometry molecular network was visualized by Cytoscape [55]. After that, the mass spectrometry data were imported into the UNIFI 1.7 scientific information system for peak extraction, peak matching analysis, and processing. The final identification of the compounds was achieved by matching and analyzing the extracted peaks with the Reaxys database (https://www.reaxys.com) and an in-house database of *Ganoderma* established in our previous study [56], which contained information on 960 metabolites that have been reported in *Ganoderma* species.

### Quantification of triterpenoids

*Ganoderma* triterpenoid standards were purchased from Chengdu Pusi Bio-technology Co., Ltd., including ganoderic acid C2 (GC2, Lot# PS000597, purity 99.28 %), ganoderic acid C6 (GC6, Lot# PS010883, purity 99.33 %), ganoderic acid D2 (GD2, Lot# PS011345, purity 99.18 %), ganoderic acid F (GF, Lot# PS240105-04, purity 99.11 %), ganoderic acid G (GG, Lot# PS010838, purity 96.76 %), ganoderic acid B (GB, Lot# PS010857, purity 99.41 %), ganoderic acid A (GA, Lot# PS010388, purity 99.89 %), ganoderic acid H (GH, Lot# PS010902, purity 97.14 %), ganoderic acid K (GK, Lot# PS231020-04, purity 99.51 %), ganoderenic acid D (GED, Lot# PS010884, purity 99.51 %), ganoderic acid C1 (GC1, Lot# PS010882, purity 99.84 %), and Lucidenic acid A (LA, lot# PS010891, purity 98.44 %). Twelve triterpenoid standards were accurately weighed and dissolved in methanol to prepare a 0.5 mg/mL stock solution, respectively. The working standards solutions were prepared by diluting the stock solution with methanol to 5, 10, 50, 100, 500, and 1000 ng/mL.

Fifty milligrams of GLSP were ultrasonically extracted with 50 mL of 80 % methanol for 30 min, then centrifuged at 13,000 r/min for 10 min. Use the supernatant as the sample solution. Then, the working standard solution and the sample solution were subjected to ultra-performance liquid chromatography (UPLC)-multiple reaction monitoring mass spectrometry (MRM-MS) analysis.

The UPLC-MRM-MS method for the quantitation was adopted from our established methodology [57] with slight modifications. An ACQUITY I-CLASS UPLC system coupled with an Xevo TQ-S mass spectrometer (Waters, USA) was used for the determination. Chromatographic separation was achieved using a Waters ACQUITY UPLC HSS T3 (1.8 μm, 2.1 × 100 mm; Waters) maintained at 40 °C. The mobile phase consisted of (A) 0.1 % formic acid in water and (B) 0.1 % formic acid in acetonitrile, delivered at 0.40 mL/min with the following gradient program: 0–0.5 min, 20 % B (isocratic); 0.5–3 min, 20–26.5 % B; 3–10 min, 26.5–48 % B; 10–11 min, 48–100 % B; 11–13 min, 100 % B (wash). The injection volume was 2 μL. The mass spectrometer was operated in negative electrospray ionization (ESI) mode. The parameters were set: the ion source temperature was 150 °C; the capillary voltage was 3.5 kV; the desolvation gas temperature was 500 °C; the desolvation gas flow rate was 1000 L/h; the collision gas flow was 0.15 mL/min; the acquisition mode was multiple reaction monitoring (MRM), the monitoring ion pairs, cone voltage (CV) and collision energy (CE) were presented in Supplementary Table S4.

### Molecular docking

The 2D chemical structures of triterpenoids were constructed using KingDraw (v1.0.4; https://www.kingdraw.cn/) and subsequently converted to 3D structures using openbabel (v3.1.1) [58]. The chemical structures of the well-known agonists R-848, CL097, and CL075 for Toll-like receptor (*TLR*) 7/8 [59,60], 2′,3′-cGAMP, Cyclic di-GMP, and MIK-1454 for stimulator of interferon genes (*STING*) [61,62], and RO8191 [63] for IFN-α/β receptor 2 (*IFNAR2*) were retrieved from PubChem (https://pubchem.ncbi.nlm.nih.gov/) [64]. The agonists of *STING* can only be downloaded in a 2D structure, and were converted to 3D by openbabel; the other molecules can be directly downloaded in a 3D structure. The structures of target proteins, including *TLR7* (PDB ID: 5GMH), *TLR8* (PDB ID: 3W3N), *STING* (PDB ID: 4KSY), and the binary complex of IFN-α2 and *IFNAR2* (PDB ID: 3S9D), were obtained from the Protein Data Bank (https://www.rcsb.org/pdb/) [65]. Notably, the species origin of *TLR7* is the macaque, which shares 96.8 % similarity with the human *TLR7* sequence [66]. The species origin of the remaining target proteins was human. In addition, we isolated the *IFNAR2* structure from the IFN-α2-*IFNAR2* complex to check the binding ability and sites of the agonist RO8191 and triterpenoids to *IFNAR2* individually.

The QuickVina (v2.1.0) [67] was used to perform molecular docking, which includes the blind docking tool QuickVina-W and the accurate docking tool QuickVina2. The center coordinates of grid boxes were acquired from the published complexes of agonists with their targets. Specifically, the grid box centers for molecular docking were defined as follows: (1) the center of mass of R-848 was used for *TLR7* [66] and *TLR8* [59] simulations, and (2) the center of mass of 2′,3′-cGAMP served as the reference point for *STING* simulations [61]. For *IFNAR2* agonist RO8191, we performed blind docking using QuickVina-W and obtained the center of mass coordinates from the optimal docking mode, since there are no known docking sites. The size of all the grid boxes is 20 × 20 × 20 Å. All the ligands and target proteins were preprocessed by the AutodockTools (v1.5.7) module [68] before being subjected to docking by QuickVina2. Finally, 20 binding modes of each docking were obtained, and the resulting optimal docking modes were visualized by Pymol (v3.1.0) [69]. The sources and the center coordinates of docking for all target proteins and their agonists are shown in Supplementary Table S5.

### Cell culture, stimulation, and administration

PBMCs (Meisen Cell, Cat# hPB010C and Cat# W-hPB010C) were thawed and seeded at a density of 1 × 10^6^ cells per well in a 24-well plate using RPMI 1640 medium (Gibco, Cat# C11875500BT) supplemented with 10 % FBS and 1 % penicillin–streptomycin (Gibco, Cat# 15140122). The seven ganoderic acids standards were purchased from Chengdu Pusi Bio-technology Co., Ltd, including GA (Lot# PS010388, purity 99.89 %), GB (Lot# PS011342, purity 99.31 %), GC1 (Lot# PS010882, purity 99.84 %), GC2 (Lot# PS010837, purity 99.30 %), GD2 (Lot# PS011345, purity 99.18 %), GF (Lot# PS013922, purity 95.81 %), and GH (Lot# PSD250718-521, purity 95.05 %). All the ganoderic acid standards were initially dissolved in DMSO and subsequently diluted in complete culture medium to yield final concentrations of either 40 μg/mL or 10 μg/mL upon administration. The control group and the group stimulated with recombinant 2019-nCoV S1 protein (NavoProtein, Cat# DRA35) alone received complete medium containing no more than 0.1 % DMSO. After 24-hour pretreatment with ganoderic acids, PBMCs in all experimental groups (except controls) were stimulated with 3 μg/mL 2019-nCoV S1 protein. After 12 h post-stimulation, supernatants were collected, and the total RNA was extracted for subsequent analysis. All participating PBMC collection centers obtained institutional ethical approval before sample acquisition, and the details of collection centers, donors, and the ethical approval information can be found in Supplementary Table S6.

### RNA extraction and real-time quantitative polymerase chain reaction (RT-qPCR)

The total cellular RNA was extracted from the cultured PBMC following the manufacturer’s instructions of the RNA extraction kit (AidLab, Cat# RN28). The isolated RNA was then reverse-transcribed into cDNA using the HiFiScript cDNA Synthesis Kit (CWBIO, Cat# CW2569M) following the provided protocol. Following this, the qRT-PCR was subsequently performed in a total reaction volume of 10 µL, using the Hieff UNICON® qPCR SYBR Green Master Mix Kit (Yeasen, Cat# 1184ES08) according to the manufacturer’s instructions. The 2^-ΔΔCt^ method is employed for the calculation of relative gene expression levels. The primer sequences of each gene are documented in Supplementary Table S7.

### Statistical analysis

Differences in the percentage of antibody neutralization inhibition and gene scores between groups were assessed using the Wilcoxon test. Serum IFN-α concentrations between the control and GLSP intervention groups post-vaccination were compared using an independent samples t-test. For the in vitro cellular experiments, comparisons of IFN-α concentrations and relative gene expression levels across multiple groups were conducted using the Kruskal-Wallis test, followed by Dunn’s post-hoc test. For all tests, *p* < 0.05 indicates a significant difference.

## Results

### GLSP increases antibody levels in individuals with moderate baseline immunity

**Fig. 1:**
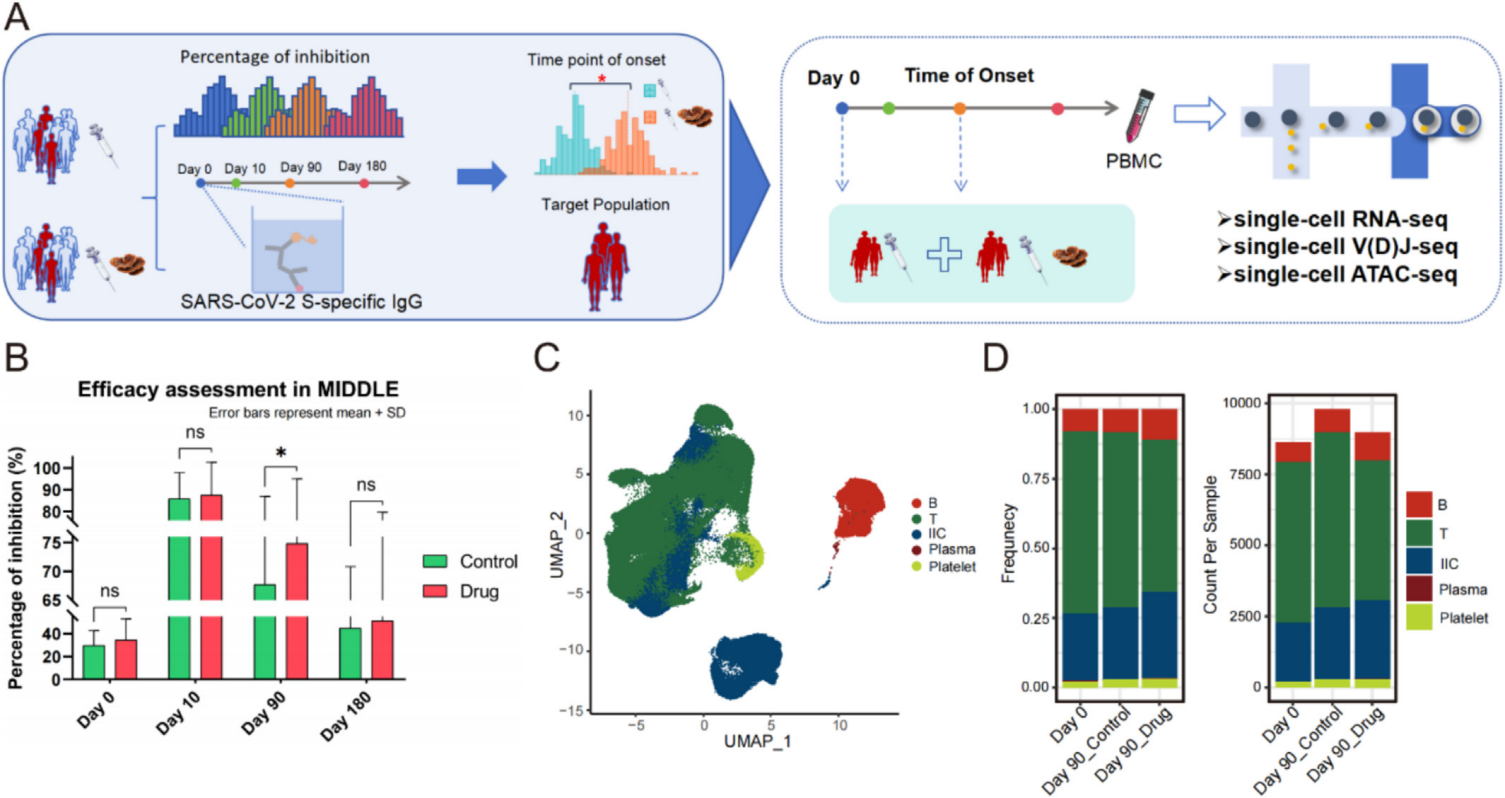
**Efficacy evaluation of GLSP and scRNA-seq profiling of PBMCs.** (A) Overall of the study design. (B) Effect of GLSP on the percentage of inhibition in the MIDDLE population at different time points. (C) UMAP representation of the scRNA-seq landscape of PBMCs, 5 major cell types were annotated with different colors. (D) The frequency and counts per sample of all cell types at different time points and groups. Significant differences in (B) were determined by the Wilcoxon test (*p < 0.05, ns *− p* > 0.05). The error bars represent the standard deviation (SD), and the horizontal bars show the mean values of the percentage of inhibition. Day 0: pre-injection; Day 90_Control: the control group 90 days post-injection; Day 90_Drug: the GLSP intervention group 90 days post-injection. MIDDLE: the population with moderate baseline immunity.

In this study, we evaluated the effect of GLSP as an adjuvant of the booster vaccine for COVID-19 and identified a significant time point at which the adjuvant effect was observed (Fig. 1A). All participants (195 individuals) were given a booster shot of the SARS-CoV-2 vaccine, in which the drug intervention group (98 individuals) received GLSP, while the rest used a blank control (97 individuals). Peripheral blood samples of all volunteers were collected before injection and at 10, 90, and 180 days after injection, and the NAbs percent inhibition in blood samples was detected. The general information of all volunteers is presented in Supplementary Table S1.

Specifically, we measured SARS-CoV-2 S-protein-specific IgG levels in the peripheral blood of all subjects before injection to determine their baseline levels of viral resistance. Three groups of populations were defined based on varying levels of basal immunity: the “high” population included individuals in the top 25 % of serum SARS-CoV-2 S protein-specific IgG concentrations, the “low” population consisted of those in the bottom 25 %, and the “middle” population encompassed individuals with concentrations falling between these two extremes. Then, we assessed the effect of GLSP on the percent inhibition. GLSP showed no significant influence on the high or low population (Supplementary Table S2, Supplementary Fig. S1A–B). However, in the middle population, GLSP markedly increased the percent inhibition of NAbs 90 days after vaccination (*p* < 0.05) (Fig. 1B). These results suggest that GLSP has a notable adjuvant effect on the immune system of individuals with moderate baseline immunity, with a significant onset of effect observed 90 days post-injection.

### GLSP amplifies the efficacy of vaccine booster effects on the immune cell proportion

Single-cell multi-omics sequencing was employed to elucidate the mechanism of action of GLSP. Before injection and at the time of onset, six subjects were selected for each group within the individuals with moderate baseline immunity, with an equal number of males and females. Then, we performed scRNA-seq, scV(D)J-seq, and scATAC-seq on extracted PBMC samples from those subjects.

The initial step was to construct a transcriptome landscape at the single-cell level. After quality filtering, we obtained a total of 215,993 cells with high quality and identified five major cell types according to known markers: B cells (*CD19*^+^), T cells (*CD3*^+^), innate immune cells (*PTPRC*^+^*CD19*^-^*CD3E*^-^), plasma cells (*CD19*^+^*MZB1*^+^), and platelets (*PF4*^+^) (Fig. 1C, Supplementary Fig. S1C, Table S8). The administration of vaccine booster injections was observed to affect the proportion of immune cell composition, with an increase in the proportion of B cells and innate immune cells, alongside a decrease in the proportion of T cells. Notably, following the administration of GLSP, the vaccine induced a similar trend but with a greater magnitude of change in these cell proportions (Fig. 1D).

### Increased proportion and anti-virus response of classical monocytes after GLSP intervention

**Fig. 2:**
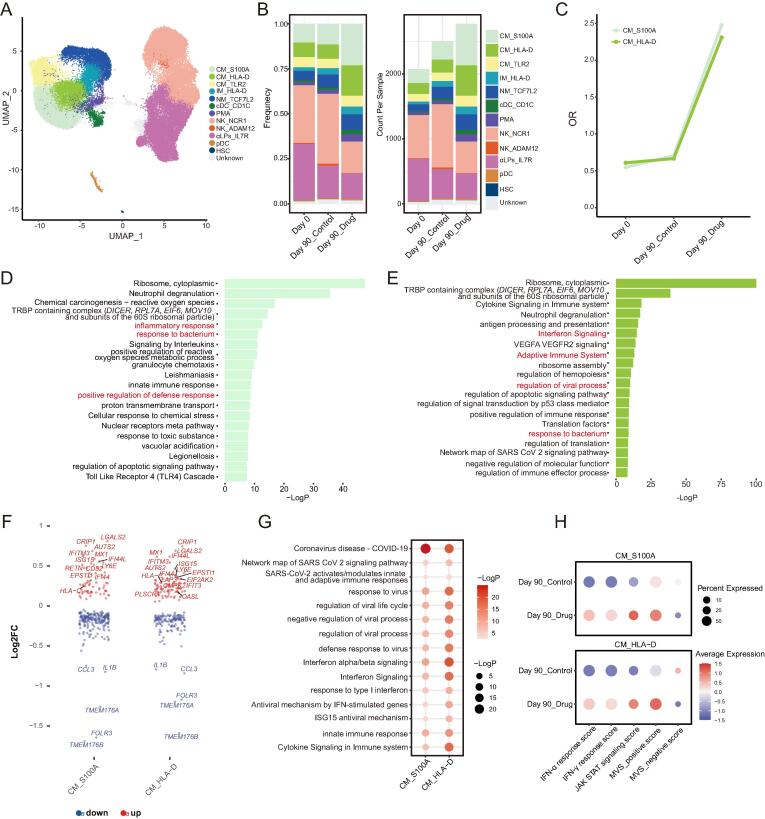
**GLSP elevated the myeloid proportions and up-regulated IFN response in classical monocytes.** (A) UMAP representation of innate IICs, 13 sub-clusters were annotated with different colors. (B) The frequency and counts per sample of different sub-clusters. (C) The OR changes of CM_S100A and CM_HLA-D at different time points and groups. (D–E) The enriched summary terms of up-regulated genes of CM_S100A (D) and CM_HLA-D (E) among myeloid cells. (F) The DEGs of the GLSP-intervention group versus the control group 90 days post-vaccination of CM_S100A and CM_HLA-D. The Wilcoxon test was applied in the DEGs analysis, and the *p*-values were adjusted by the Bonferroni method. (G) Terms related to the response to virus and interferon signaling were enriched by GLSP upregulated genes. (H) Scores of IFN-α response, IFN-γ response, positive and negative MVS in different groups post-vaccination of CM_S100A and CM_HLA-D. Day 0: pre-injection; Day 90_Control: the control group 90 days post-injection; Day 90_Drug: the GLSP intervention group 90 days post-injection.

GL ingredients can assist in enhancing vaccine effectiveness by activating innate immune cells, especially antigen-presenting cells (APCs), and further activating helper T (Th) cells and B cells [[26], [70], [71]]. According to well-known markers and distinct expression patterns of cells, we divided innate immune cells (IICs) into 13 sub-clusters, including natural killer cells (NK, *NCR*1^+^), α-lymphoid progenitor cells (αLPs, *IL7R*^+^), a precursor cell of the innate lymphoid cell (ILC), plasmacytoid dendritic cells (pDC, *IL3RA*^+^), hematopoietic stem cells (HSC, *CD34*^+^), and multiple myeloid cells (*CST3*^+^*LYZ*^+^) (Fig. 2A, Supplementary Fig. S2A, Table S8). Myeloid cell subsets included various sub-types of monocytes, including classical monocytes (*CD14*^+^*FCGR3A*^-^, CM), intermediate monocytes (IM, *CD14*^+^*FCGR3A*^+^), non-classical monocytes (*CD14*^-^*FCGR3A*^+^, NM), and platelet-monocyte aggregates (*PF4*^+^, PMA), as well as classical dendritic cells (*CD1C*^+^, cDC). The proportion of myeloid cell clusters exhibited only a marginal increase after vaccination alone following the post-injection booster, while a substantial elevation was observed after the administration of the GLSP (control group − Day 90: 36.69 %; GLSP intervention group − Day 90: 61.34 %) (Fig. 2B). In particular, the proportions of the two CM sub-clusters, CM_S100A and CM_HLA-D, doubled in comparison with injection alone (Fig. 2B), with a clear bias in the GLSP intervention group (Fig. 2C, Supplementary Fig. S2B). In contrast, the proportion of αLPs showed a decreasing trend after the vaccination, with an even more pronounced decrease observed after the GLSP intervention. Additionally, NK cell percentage was increased with the vaccine booster injection alone but decreased after the GLSP intervention, indicating an inhibitory effect on the innate cell-killing function of GLSP.

CM tends to be predominant in monocytes and has prominent functions in pro-inflammatory responses, pathogen phagocytosis, and antigen presentation [[72], [73], [74], [75]]. We performed the enrichment analysis of the differentially expressed genes (DEGs), and the enriched terms showed that both CM_S100A and CM_HLA-D are active in defending against viruses (Fig. 2D–E). Additionally, ribosomal protein genes were enriched, implying that the two cell sub-clusters have active ribosome synthesis and protein translation [76]. CM_S100A is characterized by a high level of S100 calcium-binding proteins (Supplementary Fig. S2A), which are associated with a robust inflammatory response [[77], [78]] (Fig. 2D). In contrast, CM_HLA-D excels in MHC-II-mediated antigen processing and presentation, with strong IFN signaling (Fig. 2E).

We then found that GLSP had a consistent effect on transcript changes in both CM_S100A and CM_HLA-D. Numerous genes related to IFN-α stimulation were upregulated by GLSP in comparison to vaccination alone. Conversely, GLSP downregulated the expression of inflammation-related genes such as *IL1B* and *CCL3* in CMs (Fig. 2F). Therefore, GLSP potentially increases innate antiviral resistance but negatively modulates the inflammatory response. This is beneficial for safer immunization. Indeed, upregulated genes were enriched for multiple antiviral responses and type I IFN signaling-related entries (Fig. 2G, Supplementary Fig. 2C–D). Moreover, gene set scoring demonstrated the significant enhancement of both IFN-α and IFN-γ responses on the CM_S100A and CM_HLA-D consistently (Fig. 2H, Supplementary Fig. S2E). Activation of downstream antiviral responses following the interaction of IFN binding to its receptor is mediated through the *JAK-STAT* pathway [79]. In this study, GLSP also consistently induced a more active *JAK-STAT* signaling than vaccination alone (Fig. 2H, Supplementary Fig. S2E). MVS represents a set of conserved signature genes that emerge following respiratory virus infection [45]. These genes can be divided into two distinct categories: the positive MVS genes are typically upregulated in a multitude of respiratory infections, whereas the negative MVS genes are generally downregulated. After GLSP intervention, positive MVS scores were elevated while negative MVS scores were reduced in both monocyte populations, suggesting an overall increase in immune response to the vaccine (Fig. 2H, Supplementary Fig. S2E). In summary, GLSP enhances IFN-mediated innate antiviral capacity through action on CM clusters.

### GLSP modulates the activity of transcription factors and chromatin accessibility for genes involved in the IFN-α response

**Fig. 3:**
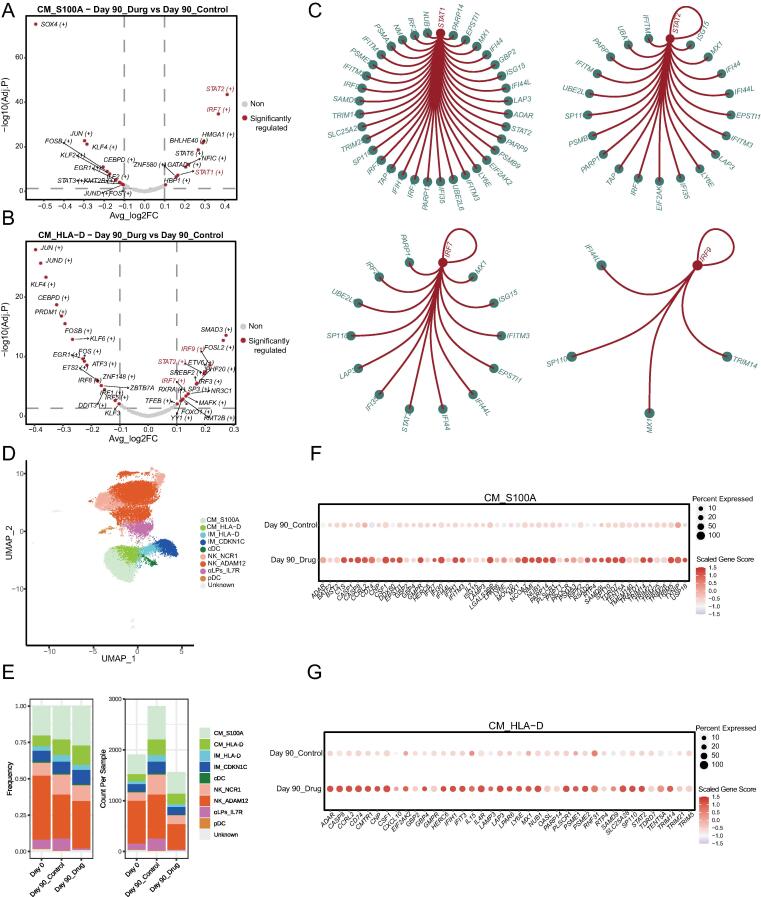
**GLSP affected the upstream regulation in classical monocytes to enhance IFN-α response.** (A–B) Differences in TF regulon activity between the GLSP intervention and control after vaccination of CM_S100A (A) and CM_HLA-D (B). The *P*-values were adjusted by the BH methods. (C) TF-targets networks of STAT1, STAT2, IRF7, and IRF9. (D) UMAP representation of scATAC-seq landscape of innate immune cells, 10 sub-clusters were annotated with different colors. (E) The frequency and counts per sample of different sub-clusters. (F–G) Genes related to IFN-α-response are associated with higher gene scores after GLSP intervention CM_S100A (F) and CM_HLA-D (G). Day 0: pre-injection; Day 90_Control: the control group 90 days post-injection; Day 90_Drug: the GLSP intervention group 90 days post-injection.

Recent studies have demonstrated that immune adjuvants can exert a sustained influence on innate immunity through epigenetic modifications, which in turn alter transcription factor activity and produce a lasting impact on innate immunity [80]. Accordingly, we proceeded to investigate whether GLSP could facilitate the promotion of innate immunity by influencing upstream regulation in the context of vaccination. We utilized the SCENIC method to infer active TFs and their target genes, delineating the regulons associated with CM_S100A and CM_HLA-D. Moreover, we identified regulons exhibiting differential activity between the GLSP intervention group and the control group. SCENIC analysis revealed a total of 81 and 103 regulons for CM_S100A and CM_HLA-D, respectively, with 43 and 48 regulons exhibiting significant modulation by GLSP (Fig. 3A–B). Among them, GLSP significantly upregulated several regulons with the TFs involved in IFN-α response signaling, including *STAT1, STAT2, IRF7*, and *IRF9* [79]. Multiple target genes associated with IFN-α response were identified within these inferred regulons (Fig. 3C). In contrast, the activity of AP-1-associated regulons, such as *JUN*, *JUND*, and *FOS*, was markedly diminished after GLSP intervention in comparison to the vaccination alone. Interestingly, it has been reported that the BNT162b2 mRNA vaccine induces a significant increase in the proportion of myeloid cells enriched for IFN-responsive TFs and reduced AP-1 TFs [81]. These findings indicate that the effect of GLSP on transcriptional regulation is beneficial in enhancing vaccine-induced innate immune responses.

The single-cell scATAC-seq landscape provides insight into the epigenetic impact of GLSP. The ArchR tool was employed to complete quality control and downscaling, and clustering of cells. The annotation of IICs and their sub-clusters was conducted (Fig. 3D, Supplementary Fig. S3A, Table S8). Consistently, in the scATAC-seq profile, administration of GLSP also resulted in a more pronounced increase in the proportion of myeloid cells, particularly the two CM sub-clusters, compared with vaccination alone (Fig. 3E). ArchR calculates gene scores by assessing overall chromatin accessibility within the gene body regions. We compared the difference in gene score within the INTERFERON ALPHA RESPONSE term in the HALLMARK dataset between groups. We observed that more than 40 % (CM_S100A: 53/97, CM_HLA-D: 39/97) of IFN-α-related genes exhibited enhanced scores after the administration of GLSP, in comparison to the booster injection alone (Fig. 3F–G). Interestingly, although changes in chromatin accessibility of genes and changes in their expression shared insufficient consistency (Supplementary Fig. S3B–D), our study still demonstrated an overall positive effect of the GLSP intervention on the IFN-α response in CM sub-clusters in both contexts of chromatin accessibility (Supplementary Fig. S3E) and gene expression (Fig. 2H, Supplementary Fig. S2E). The enhancement of the chromatin accessibility of the interferon-responsive factors (IRFs) was one of the key functions of the reported vaccine adjuvant [82]. Taken together, GLSP has the potential to act as an epigenetic adjuvant that can enhance the strength of the IFN-α response by modulating upstream regulatory mechanisms.

### GLSP induces expansion of naïve B cells and improves antiviral capacity

**Fig. 4:**
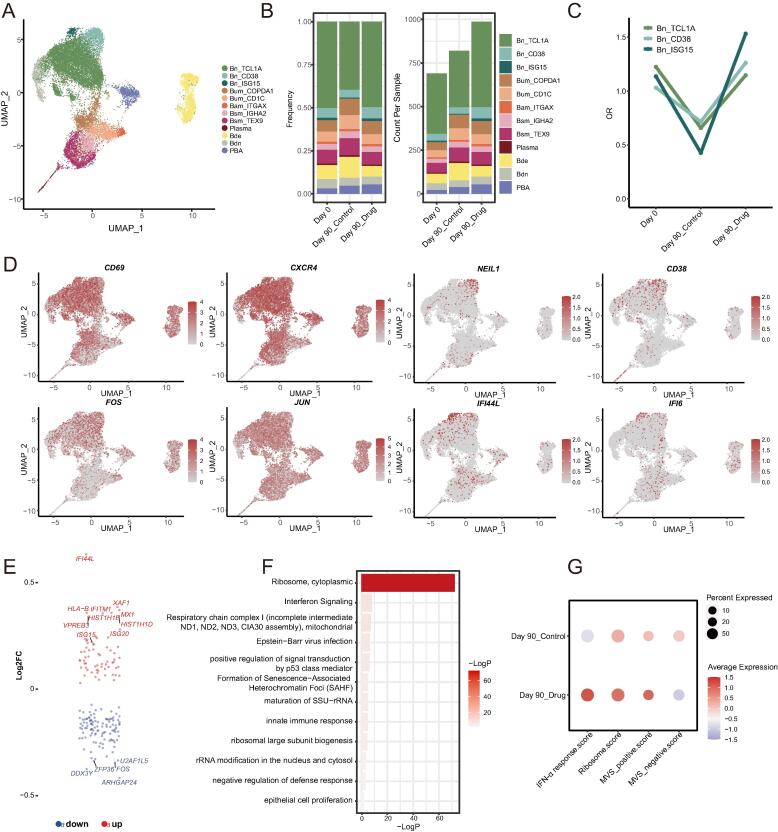
**GLSP increased activenaïve B cells and enhanced antiviral response.** (A) UMAP representation of B and plasma cells, 12 sub-clusters were annotated with different colors. (B) The frequency and counts per sample of different sub-clusters. (C) The OR changes of three naïve B clusters at different time points and groups. (D) The highly expressed active signatures, germinal center markers, and IFN response markers of naïve B clusters. (E) The DEGs of the GLSP-intervention group versus the control group 90 days post-vaccination of naïve B cells. The Wilcoxon test was applied in DEGs analysis, and the *p*-values were adjusted by the Bonferroni method. (F) The summary terms enriched by GLSP upregulated genes. (G) Scores of IFN-α response, ribosome, positive and negative MVS in different groups post-vaccination of naïve B cells. Day 0: pre-injection; Day 90_Control: the control group 90 days post-injection; Day 90_Drug: the GLSP intervention group 90 days post-injection.

B cells and plasma cells are important for antibody production and durable humoral immunity [83]. We subdivided B cells into sub-clusters and placed them in the same UMAP coordinates as plasma cells. We identified seven sub-types of B cells according to known markers (Supplementary Table S8): naïve B cells (Bn, *CD19*^+^*IGHD*^+^*IGHM*^+^*CD24*^+^), immunoglobulin (Ig)-class unswitched memory B cells (Busm, *CD19*^+^*IGHD*^+^*IGHM*^+^*CD27*^+^), atypical memory B cells (Bam, *CD19*^+^*IGHD*^+^*CD27*^-^*ITGAX*^+^), Ig-class switched memory B cells (Bsm, *CD19*^+^*IGHD*^-^*IGHM*^-^*CD27*^+^), *CD3*^+^*CD19*^+^ dual-expressing cells (Bde, *CD19*^+^*CD3E*^+^), *IgD*^-^*CD27*^-^ double-negative B cells (Bdn, *IGHD*^-^*CD27*^-^), Platelet-B aggregates (PBA, *CD19*^+^*PF4*^+^). Then, we further subdivided B cells based on their distinct expression profiles and obtained 12 sub-clusters (Fig. 4A, Supplementary Fig. S4A). We then examined the changes in B cell composition in response to vaccination and GLSP intervention. Following the booster injection alone, a reduction in the proportion of naïve B cells and an increase in the proportion of memory B cells were observed. These findings have been reported in a previous study [84]. In particular, there was a significant elevation in the proportion of Bde cells (Fig. 4B), which are capable of performing both B-cell and T-cell immune functions [85]. Nevertheless, in comparison to the pre-vaccination phase, the cell composition of the GLSP intervention group remained nearly unchanged post-injection, except for a slight increase in the proportion of PBA. In terms of the mean number of cells per sample, GLSP had a similar number of memory sub-clusters as vaccination alone; however, the number of naïve B cells increased in parallel (Fig. 4B). We used OR to measure the distributional bias of sub-clusters across the different conditions. The OR of all Bn clusters decreased after injection alone but markedly increased in the GLSP intervention group, especially the Bn_ISG15 cluster (Fig. 4C, Supplementary Fig. S4B).

*CD69* and AP-1 family genes (*FOS*, *JUN*, *JUND*, etc.) are essential for B cell activation and differentiation [[86], [87]]. High levels of those signatures in naïve B cells (Fig. 4D) indicate early activation states of those clusters. Bn_TCL1A is predominant among naïve cells, with a robust capacity to present antigens (Supplementary Fig. S4C). The DEGs of Bn_CD38 are associated with cell activation and BCR signaling, and this cluster is characterized by the germinal center (GC) markers *CD38* and *NEIL1* (Fig. 4D, Supplementary Fig. S4D). GC is the site for affinity maturation of B cells, and an elevated proportion of GC B cells is usually beneficial for memory formation and high-affinity antibody responses [[88], [89]]. Additionally, Bn_ISG15 specifically overexpresses interferon-stimulated genes with an active antiviral response (Fig. 4D, Supplementary Fig. S4E). The induction of this subpopulation may enhance the IFN response of B cells, as a result of which GLSP could exert a beneficial effect.

The present study continues to focus on the effect of GLSP on gene expression in naïve B cells. The results demonstrate that GLSP upregulated the expression of several interferon-stimulated genes (ISGs), such as *IFI44L*, *IFITM1*, *ISG15*, and *ISG20*, in comparison to the booster injection alone (Fig. 4E). Indeed, the upregulated genes were found to be associated with IFN signaling (Fig. 4F). Furthermore, the top pathway that was enriched was “Ribosome, cytoplasmic”, which suggests that GLSP similarly upregulates ribosomal gene expression and therefore can induce more active protein translation (Fig. 4F). Similarly, between-group differences in gene scores demonstrated that GLSP could further enhance the response to IFN-α, as well as ribosome production following booster injections. Additionally, GLSP induced a stronger positive MVS and a lower negative MVS, indicating that it can also enhance the conserved antiviral immune response of B cells (Fig. 4G, Supplementary Fig. S4F). In conclusion, GLSP induced the expansion of naïve B cells and enhanced their antiviral response in comparison to vaccination alone.

### GLSP expanded activated *CD8*^+^ T cells with more intense IFN response

**Fig. 5:**
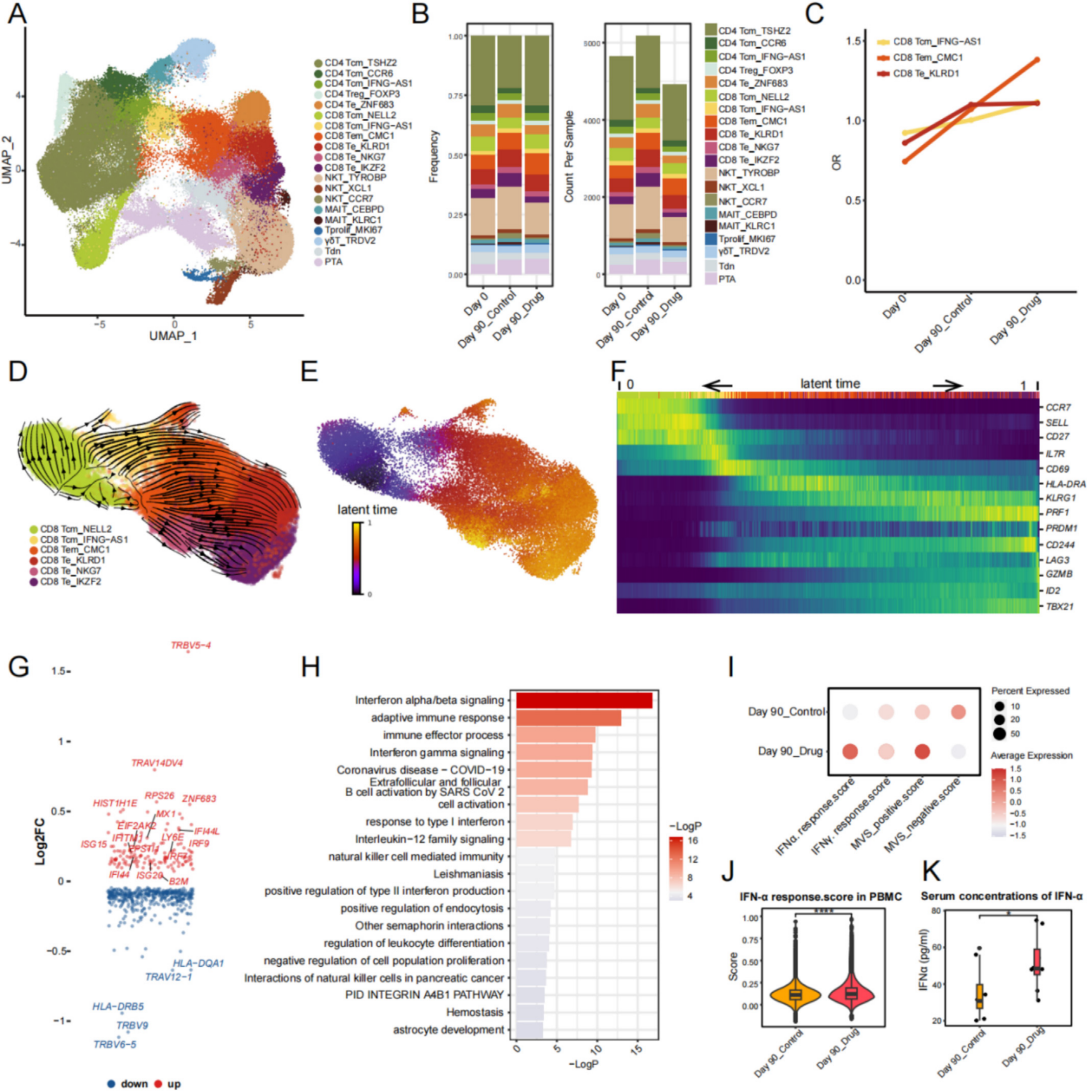
**The effect of GLSP on T cells and global IFN-α production and response. (A).** UMAP representation of T cells, 20 sub-clusters were annotated with different colors. (B) The frequency and counts per sample of different sub-clusters. (C) The OR changes of CD8 Tcm_IFNG-AS1, CD8 Tem_CMC1, and CD8 Te_KLRD1 at different time points and groups. (D) RNA velocity stream plot of CD8 clusters. (E) The latent time of CD8 clusters. (F) CD8 differentiation signatures expression on the latent time axis. (G) The DEGs of the GLSP-intervention group versus the control group 90 days post-vaccination of CD8 Tcm_IFNG-AS1, CD8 Tem_CMC1, and CD8 Te_KLRD1. The Wilcoxon test was applied in DEGs analysis, and the *p*-values were adjusted by the Bonferroni method. (H) The summary terms enriched by GLSP upregulated genes. (I) Scores of IFN-α response, IFN-γ response, and positive and negative MVS in different groups post-vaccination. (J) IFN-α response score of all PBMCs in different groups post-vaccination. (K) Serum IFN-α levels in different groups post-vaccination. Significant differences in (I) were determined by the Wilcoxon test (*p < 0.05, **p < 0.01, ***p < 0.001, ****p < 0.0001, ns *− p* > 0.05). Significant differences in (J) were determined by *t*-test (*p < 0.05, **p < 0.01, ***p < 0.001, ****p < 0.0001, ns *− p* > 0.05). Day 0: pre-injection; Day 90_Control: the control group 90 days post-injection; Day 90_Drug: the GLSP intervention group 90 days post-injection.

T-cell-dominated cellular immunity represents a pivotal mechanism underlying the long-term resistance of the body to viral pathogens [90]. We classified T cells into 20 sub-clusters based on known markers and expression profiles (Fig. 5A, Supplementary Fig. S5A, Table S8), including 11 major sub-types: *CD4*^+^ central memory T cells (CD4 Tcm, *CD4*^+^*LEF1*^+^*CD27*^+^), *CD4*^+^ regulatory T cells (CD4 Treg, *CD4*^+^*FOXP3*^+^), *CD4*^+^ effector T cells (CD4 Te, *CD4*^+^*LEF1*^-^*CD27*^-^*GZMA*^+^), *CD8*^+^ central memory T cells (CD8 Tcm, *CD8A*^+^*LEF1*^+^*CD27*^+^), *CD8*^+^ effector memory T cells (CD8 Tem, *CD8A*^+^*LEF1*^-^CD27^+^*GZMA*^+^), *CD8*^+^ effector T cells (CD8 Te, *CD8A*^+^*LEF1*^-^*CD27*^-^*GZMA*^+^), natural killer T cells (NKT, *CD4*^-^*CD8A*^-^*NCR1*^+^), mucosal-associated invariant T cells (MAIT, *SLC4A10*^+^*TRAV1*-*2*^+^), proliferating T cells (Tprolif, *MKI67*^+^), γδ T cells (γδ T, *CD4*^-^*CD8A*^-^*TRDV2*^+^), and platelet-T aggregates (PTA, *PF4*^+^). Generally, vaccination alone leads to a decrease in Tcm cells and an increase in Te, Tem, and NKT cells. Nevertheless, the cell compositions remain largely consistent after GLSP intervention compared with the pre-vaccination group (Fig. 5B). We concluded that GLSP induced an OR enhancement in Tcm and an OR reduction in cytotoxic cells compared with booster vaccination alone, thereby restoring the cellular composition to a level approximating the pre-injection level (Supplementary Fig. S5B–D),  as well as alleviating cellular toxicity and exhaustion (Supplementary Fig. S5E-F). Consistently, in IICs, GLSP similarly reduced the proportion of NK cells with innate cytotoxicity (Fig. 2B, Supplementary Fig. S2B).

However, there are still some exceptions. Upon activation, *CD8^+^* T cells typically exhibit cytotoxic phenotypes, enabling them to directly destroy infected cells [91]. Among CD8^+^ sub-clusters, the OR of CD8 Tcm_IFNG−AS1, CD8 Tem_CMC1, and CD8 Te_KLRD1 increased after injection, and GLSP intervention resulted in a more rapid increase or maintained the same level compared to vaccine alone (Fig. 5C, Supplementary Fig. S5B). For the other sub-clusters, GLSP increased or reduced the OR to the same or lower levels as the pre-injection. Among them, GLSP significantly reduced the OR of the CD8 Te_IKZF2 (Supplementary Fig. S5B). Based on the RNA velocity, we inferred the direction of CD8 T cell differentiation and then calculated the latent time of the cell state (Fig. 5D–E). CD8 Tcm_NELL2 represents the initial point in the latent phase of the T cell differentiation process, characterized by high expression of *CCR7, SELL*, and *CD27* (Fig. 5E–F). In contrast, CD8 Te_NKG7 and CD8 Te_IKZF2 predominantly localized at the end of the latent timeline, with high levels of terminal effector T cell signatures, such as *PRDM1, TBX21*, and *ID2* [92], as well as exhaustion signatures such as *CD244* and *LAG3* (Fig. 5E–F). However, CD8 Tcm_IFNG−AS1, CD8 Tem_CMC1, and CD8 Te_KLRD1 were primarily situated in the middle phase of the latent timeline and highly expressed activated signatures *CD69* and *HLA-DR,* indicating their active differential states. Additionally, their DEGs were enriched in cell differentiation and activation (Supplementary Fig. S5G-I). In summary, GLSP preferentially promotes the expansion of *CD8^+^* T sub-clusters in the differentiated and active states.

Subsequently, we proceeded to investigate the impact of GLSP on these active cell states in gene expression. The expression levels of some TCR V region genes, such as *TRBV5-4*, *TRAV14DV4*, *TRBV6-5*, and *TRBV9*, were significantly altered by GLSP compared with the booster injection alone, suggesting the role of GLSP on TCR profiling reconstruction (Fig. 5G). Unsurprisingly, GLSP similarly upregulated several IFN-α-stimulated genes in T cells, such as *IFI44L*, *IFITM1*, and *ISG15*. The enrichment analysis of the up-regulated genes revealed that GLSP not only synchronously enhanced type I and type II interferon signaling pathways but also enhanced the overall adaptive immune response (Fig. 5H).

Further, GLSP significantly enhanced the response to IFN stimulation following vaccine injection, particularly the IFN-α response (Fig. 5I, Supplementary Fig. S5J). Similarly, GLSP enhanced the conserved immune response of these active *CD8*^+^ T sub-clusters, as evidenced by increased positive MVS scores and decreased negative MVS scores (Fig. 5I, Supplementary Fig. S5J).

### GLSP promotes IFN-α induction and signaling response

The preceding results demonstrate that GLSP enhances the IFN-α response in both innate and adaptive immune cell sub-clusters (Fig. 2, Fig. 4, Fig. 5). Subsequently, we demonstrated that GLSP facilitates the vaccine booster effect in PBMCs, significantly enhancing the IFN-α response of these cells (Fig. 5J). This enhancement may be attributed to elevated levels of IFN-α in the peripheral blood. To investigate this further, we measured IFN-α concentrations in grouped serum samples collected 90 days post-vaccination. These serum samples were from sequenced subjects. It was observed that GLSP resulted in a significant increase in serum IFN-α concentrations in comparison to booster injections alone (Fig. 5K). In summary, GLSP effectively stimulates the production of IFN-α and enhances the cellular response to this cytokine.

### GLSP enhanced interaction between myeloid cells and lymphocytes

**Fig. 6:**
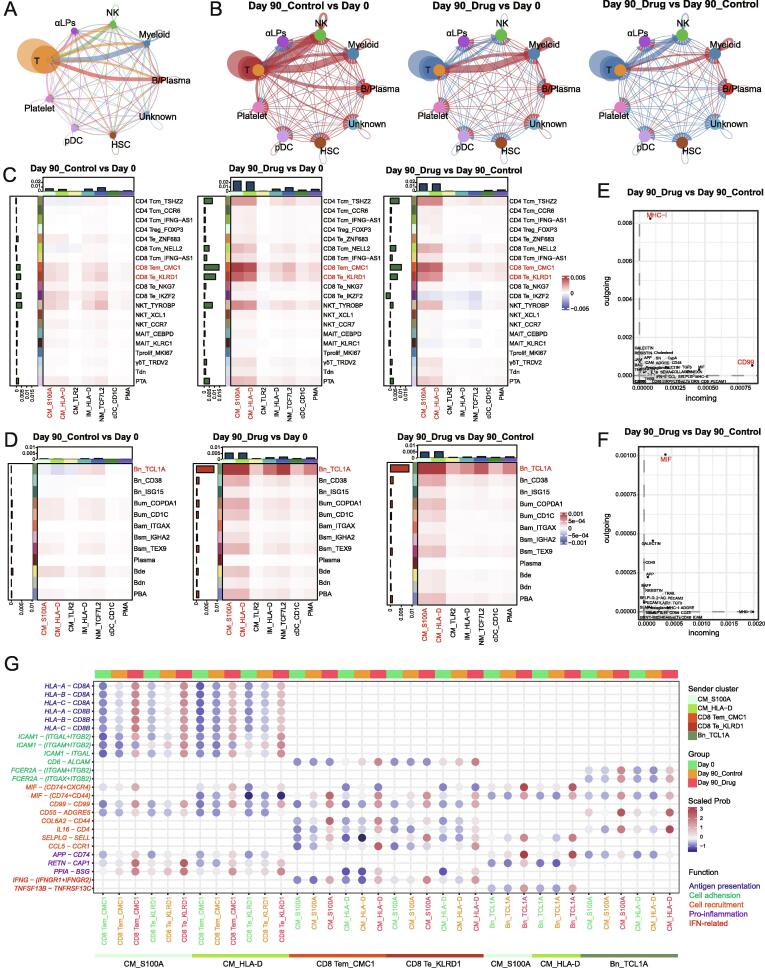
**GLSP enhanced the cell interactions between myeloid and adaptive immune cells.** (A) Interaction network of major cell types, the width of edges indicating the interaction weight strength. (B) The differential interaction weight strength among conditions. Red represents the increasing interaction, while blue represents the decreasing interaction. (C) Heatmap of interaction strength changes of myeloid clusters and T clusters among conditions. (D) Heatmap of differential interaction strength of myeloid clusters and B clusters among conditions. (E) The differential interaction strength of the GLSP-intervention group versus the control group 90 days post-vaccination of signaling pathways. CM_S100A and CM_HLA-D were set as senders while CD8 Tem_CMC1 and CD8 Te_KLRD1 were set as receptors. (F) The differential interaction strength of the GLSP-intervention group versus the control group 90 days post-vaccination of signaling pathways. CM_S100A and CM_HLA-D were set as senders while Bn_TCL1A was set as the receptor. (G) GLSP enhanced the interactions of LR pairs involved in different functions. Day 0: pre-injection; Day 90_Control: the control group 90 days post-injection; Day 90_Drug: the GLSP intervention group 90 days post-injection. (For interpretation of the references to colour in this figure legend, the reader is referred to the web version of this article.)

Furthermore, the impact of GLSP on cell–cell interactions was investigated. Among the major cell types, the interactions between myeloid cells and B/plasma cells, T cells, and NK cells were most predominant (Fig. 6A). The strength of these cell–cell interactions is influenced by both immunization and GLSP interventions. In the absence of GLSP, the booster dose of the SARS-CoV-2 vaccine was observed to enhance interactions among almost all cell types, in comparison to the state observed pre-vaccination. In contrast, the impact of vaccination on cell interactions was complex following the introduction of the GLSP intervention. In comparison to the period preceding the injection, the strength of communication between myeloid cells and all cells except HSC was enhanced, while interactions between T cells and themselves or NK cells were attenuated. Furthermore, both minor enhancements and reductions in communication were observed between other cell types. Notably, compared with vaccine booster injection alone, most interactions exhibited a reduction in strength. The most pronounced enhancement was observed in the interactions between myeloid cells and B cells, T cells, and other cell types (Fig. 6B). Furthermore, we investigated which specific sub-clusters of GLSP are predominantly involved in myeloid cell interactions with T and B cells. Injections of the vaccine booster alone induced only minor alterations in the interactions between sub-clusters compared to the pre-vaccination period. In contrast, the administration of GLSP induced more pronounced alterations in communication strength. Notably, interactions involving CM_S100A and CM_HLA-D in myeloid cells with CD8 Tem_CMC1 and CD8 Te_KLRD1 in T cells, as well as Bn_TCL1A in B cells, were significantly enhanced when compared to either the pre-vaccination or the vaccine booster injection alone (Fig. 6C-D). Taken together, GLSP simultaneously enhanced both the proportions of the aforementioned sub-clusters and the strength of their interactions, in comparison to vaccination without intervention (Fig. 2, Fig. 4, Fig. 5).

Afterward, we focused on specific communication signaling pathways and ligand-receptor (LR) pairs that differed in interaction strength among conditions. It was found that the interaction of CM_S100A and CM_HLA-D with CD8 Tem_CMC1 and CD8 Te_KLRD1 on most signaling pathways was enhanced after vaccination (Supplementary Fig. S6A). Furthermore, the administration of GLSP was observed to substantially enhance the strength of communication of these signaling pathways (Fig. 6E, Supplementary Fig. S6B). Compared with the vaccine alone, MHC-I was the top enhanced outgoing pathway of CM_S100A and CM_HLA-D to CD8 Tem_CMC1 and CD8 Te_KLRD1, indicating the ability of GLSP to promote antigen processing and presentation; among the incoming signalings, the *CD99* was mostly increased, implying that GLSP might be beneficial for activation of TCR signaling [93] and T cell migration [[94], [95], [96]] (Fig. 6E). The interaction of CM_S100A and CM_HLA-D with Bn_TCL1A resulted in an attenuation of the majority of pathway interactions when compared to the pre-injection state (Supplementary Fig. S6C). In contrast, GLSP resulted in the majority of the signaling pathways being amplified in comparison to the pre-injection state or vaccine alone (Supplementary Fig. S6D). The enhancement of communication of the *MIF* pathway was most pronounced in the GLSP in comparison to the vaccine alone (Fig. 6F). *MIF* (macrophage migration inhibitory factor) can interact with *CD74* and *CXCR4*, thereby exerting influence over the recruitment of immune cells, modulation of the inflammatory response, and cell proliferation [[97], [98]]. Thus, by enhancing the interaction between monocytes and naïve B cells, GLSP can promote B cell recruitment, proliferation, and inflammatory responses.

Specifically, GLSP reinforces interactions between LR pairs involved in multiple functions. As mentioned earlier, GLSP intervention elevated antigen presentation mediated by a variety of MHC-I molecules and concomitantly elevated *ICAM*, *ALCAM*, *SELPG*, and *FCER2A*-mediated cellular adhesion to enhance stability during cell junctions, in comparison with pre-vaccination and booster vaccination alone. Meanwhile, GLSP elevated the interaction of *MIF*-(*CD74/CXCR4/CD44*), *CD55-ADGRE5*, *COL6A2-CD44*, *IL16-CD4*, *SELPLG*-*SELL*, and *CCL5-CCR1*, to promote cell migration and recruitment. The promoted strength of *APP-CD74*, *RETN-CAP1*, and *PPIA-BSG* could indicate a pro-inflammatory response. It is noteworthy that the *IFNG-IFNGR* effect was significantly higher in comparison to the pre-injection and control conditions post-injection, indicating that the stimulation of monocytes by *CD8^+^* T cells by IFN-γ was enhanced following the administration of GLSP. This may be a contributing factor to the elevated IFN-γ response scores that were previously observed (Fig. 6G). *TNFSF13B* (BAFF) has an important role in the survival and activation of B cells, which is induced to increase by IFN-α and IFN-γ [[99], [100]]. It can be inferred that the enhanced interaction between *TNFSF13B-TNFRSF13C* may be due to enhanced IFN stimulation by GLSP.

### Modifications in BCR repertoire by GLSP associated with enhanced antiviral resistance

**Fig. 7:**
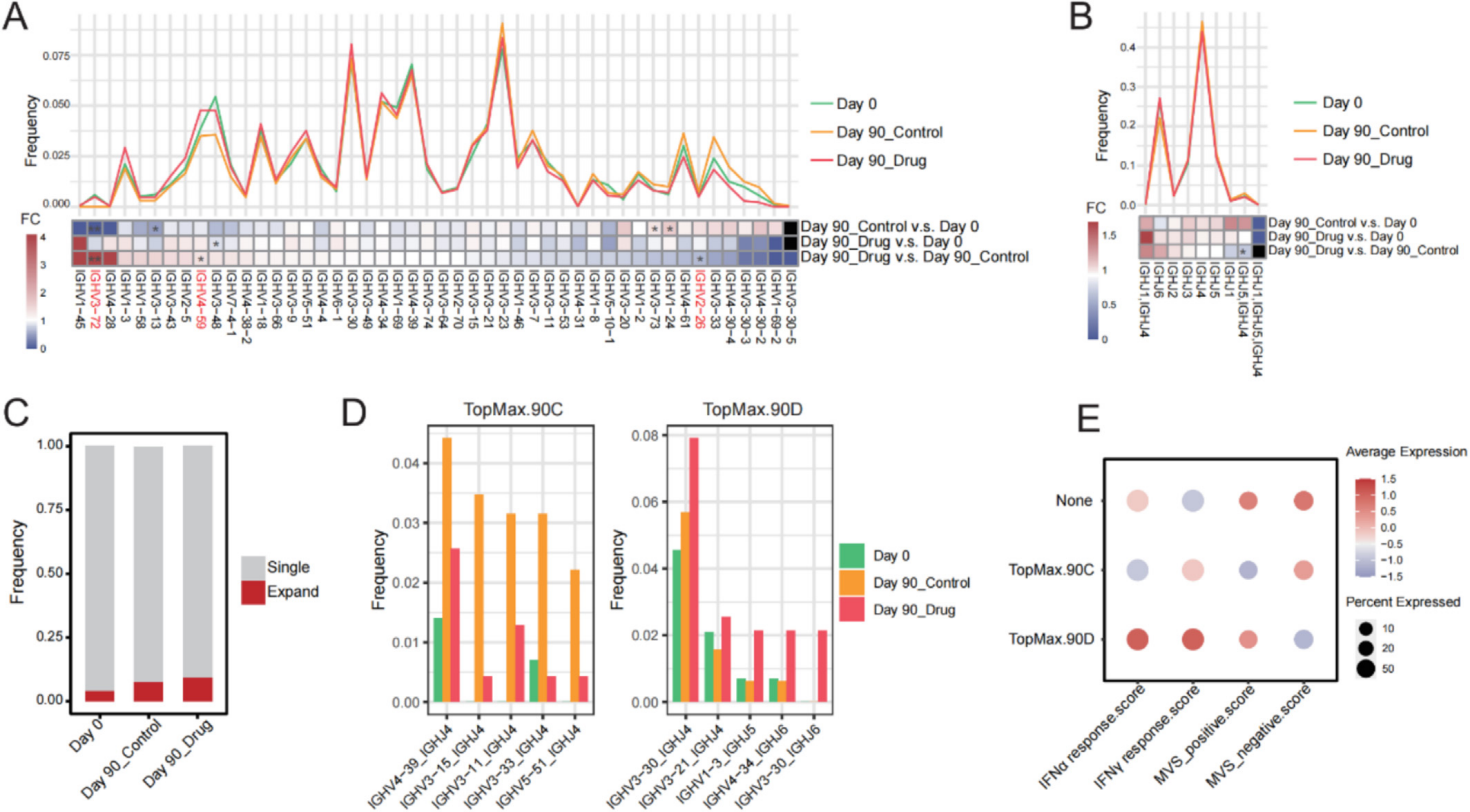
**GLSP changed the BCR gene and clone dominance to affect the antiviral ability** (A) Usage frequency and difference across conditions of IGHV genes. (B) Usage frequency and difference across conditions of IGHJ genes. (C) The percentage of single and expanded clones at different time points and groups. (D) The IGHV-J pair of TopMax.90C and TopMax.90D. (E) Scores of IFN-α response, IFN-γ response, positive and negative MVS in TopMax.90C, TopMax.90D, and other IGHV-J pairs. Significant differences in (A--B) were determined by the Wilcoxon test (*p < 0.05, **p < 0.01, ***p < 0.001, ****p < 0.0001, ns *− p* > 0.05). CDR3: complementarity-determining region 3. Day 0: pre-injection; Day 90_Control: the control group 90 days post-injection; Day 90_Drug: the GLSP intervention group 90 days post-injection.

Next, we utilized Dandelion for quality control and downstream analysis of scV(D)J-seq data, to trace the changes in BCR repertoire in response to the COVID-19 vaccine booster and GLSP. Overall, after vaccination, the control and drug intervention groups showed very different trends in changes in gene usage (Fig. 7A–B, Supplementary Fig. S7A–B). In comparison to the vaccine booster injection alone, the GLSP resulted in a bias towards *IGHV3-72* and *IGHV4-59*, as well as a notable reduction in the frequency of *IGHV2-26*. Interestingly, the reported BCR repertoire demonstrated a notable inclination towards *IGHV3-72* following BBIBP-CorV, while SARS-CoV-2 infection led to a reduction in the usage of *IGHV4-59* and an enhancement in the usage of *IGHV2-26* [101]. These findings suggest that the GLSP-modified BCR repertoire may establish a distinct B-cell immune profile that targets vaccine antigens rather than responding directly to viral invasion. Subsequently, we examined alterations in the length of amino acids in the CDR3. In the heavy chain, CDR3 was noticeably shortened after injection without GLSP administration, while GLSP extended CDR3 length to the pre-injection level (Supplementary Fig. S7C); in the light chain, the CDR3 length was reduced after vaccination, especially with GLSP intervention (Supplementary Fig. S7D).

Further, vaccination and GLSP administration also affected the clonal dominance of BCR. GLSP further increases the proportion of expanded clones based on vaccination (Fig. 7C). Within the expanded clones, gene pairing of *IGHV3-23* or *IGHV3-30* with *IGHJ4* was observed to be prevalent both before and after the injections (Supplementary Fig. S7E). Of note, this enrichment was most pronounced for the injection group following GLSP intervention. However, there is still some bias in the enrichment of gene pairs in different groups. To capture this bias, we defined two sets of gene pairs enriched in control and drug intervention after injection, named “TopMax.90C” and “TopMax.90D” separately. The included gene pairs had to fulfill two criteria: 1) the frequency of the gene pair was the highest within the group in comparison to the pre-injection and other groups; 2) the frequency of this pair was among the top five within the group. In TopMax.90D, two pairs were found to contain the *IGHV3-30* (Fig. 7D). The frequency of this gene was significantly increased after one dose of BBIBP-CorV in recovered patients [101], suggesting a possible important role of the BCR of this V gene for early vaccine response.

We then explored the difference in the antiviral ability of the TopMax.90C and TopMax.90D. B cells with V-J pairs in TopMax.90D exhibited augmented IFN-α and IFN-γ responses relative to those in TopMax.90C and other pairs (Fig. 7E). In addition, TopMax.90D had higher positive MVS and lower negative MVS scores than TopMax.90C, suggesting that these GLSP-induced enriched gene pairings are associated with conserved respiratory viral responses (Fig. 7E). In conclusion, the GLSP treatment resulted in a shift in the BCR genes and clonal advantages following injection, which may be associated with higher antiviral resistance.

### Glsp-specific expanded TCR clones with stronger IFN-mediated antiviral response and enhanced affinity for virus

**Fig. 8:**
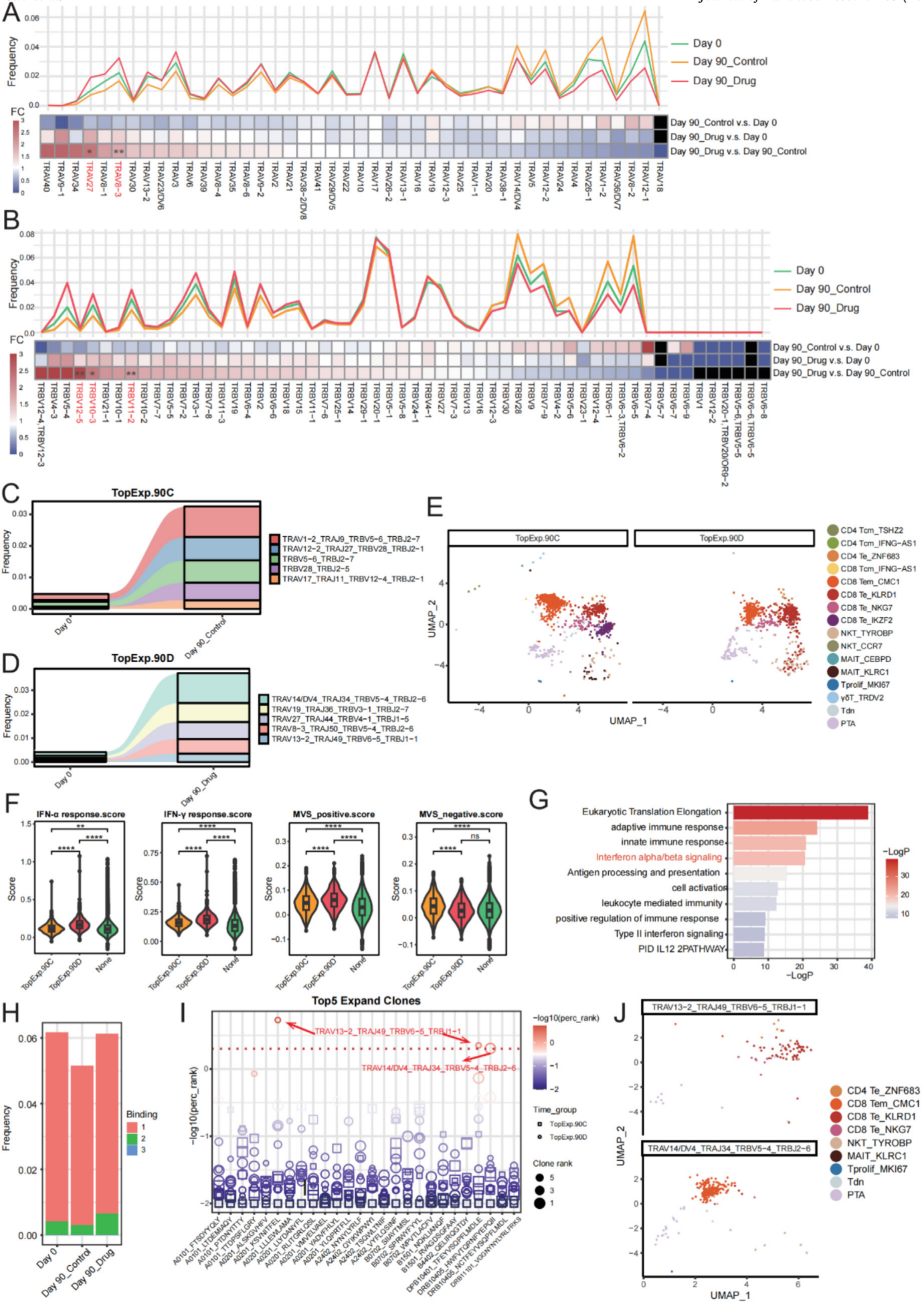
**GLSP induced an enhanced IFN response and virus affinity by selectively expanding TCR clones** (A) Usage frequency and difference across conditions of TRAV genes. (B) Usage frequency and difference across conditions of TRBV genes. (C-D) The V-J pairs of TopExp.90C (C) and TopExp.90D (D). (E) The cell distribution of TopExp.90C and TopExp.90D. (F) Scores of IFN-α response, IFN-γ response, positive and negative MVS in TopMax.90C, TopMax.90D, and other IGHV-J pairs. (G) The enriched terms of up-regulated genes in TopMax.90D versus TopMax.90C. (H) The frequency of TCRs predicted to bind to SARS-CoV-2. (I) The percentage of binding score rank of TopExp.90C and TopExp.90D with all SARS-CoV-2 epitopes in MixTCRpred. The red dashed line annotates the top 0.5 % rank threshold. The V-J pairs of two clones predicted to be able to bind to SARS-CoV-2 were noted. Their cell distribution was shown in (J). Significant differences in (A), (B), and (F) were determined by the Wilcoxon test (*p < 0.05, **p < 0.01, ***p < 0.001, ****p < 0.0001, ns *− p* > 0.05). CDR3: complementarity-determining region 3. Day 0: pre-injection; Day 90_Control: the control group 90 days post-injection; Day 90_Drug: the GLSP intervention group 90 days post-injection. (For interpretation of the references to colour in this figure legend, the reader is referred to the web version of this article.)

The following section examines the alterations in the TCR repertoire. Similar to its effect on the use of BCR genes, GLSP altered the trend in the use of TCR genes compared to vaccination alone (Fig. 8A–B, Supplementary Fig. S8A–B). Totally seven genes showed a statistically significant change in usage in response to GLSP. In particular, the frequency of *TRBV11*-*2* was markedly increased after drug intervention, which is correlated with inflammation and T-cell activation in multisystem inflammatory syndrome in children (MIS-C) following SARS-CoV-2 exposure [102]. This indicated that GLSP plays a role in enhancing the immune response by shaping the TCR repertoire. Further, CDR3 length was significantly increased after GLSP intervention, both in the TCR α chain and β chain, in comparison with pre-injection and vaccine injection alone (Supplementary Fig. S8C–D).

Then, to investigate the effect of GLSP on clone expansion, we selected the clones with the highest fold change in frequency compared to the pre-injection period and with a significant number of clones (>50) for the control and drug intervention groups. We named them “TopExp.90C” and “TopExp.90D”. The two sets of clonotypes had distinctive α-β chain genes, implying that GLSP intervention induces differential selection for expansion (Fig. 8C-D). The phenotype of TopExp.90C and TopExp.90D was mostly *CD8*^+^ Te or Tem cells, suggesting the important role of *CD8^+^* T cells in vaccine-induced specific T-cell memory formation (Fig. 8E) [103]. However, the CD8 Te_IKZF2 had a limited distribution in TopExp.90D, which is consistent with the observed inhibition of GLSP to this sub-cluster (Supplementary Fig. S5B). We further linked the clonal dominance to its functionality. Compared to the other clones, both TopExp.90C and TopExp.90D have higher IFN response and positive MVS, and lower negative MVS, especially TopExp.90D (Fig. 8F). We also identified the DEGs between those two expanded clone sets. Predictably, TopExp.90D was associated with improved type I and type II IFN signaling, along with stronger innate and adaptive immune responses (Fig. 8G).

Afterward, we utilized MixTCRpred to measure the affinity of TCRs to SARS-CoV-2. Following the criteria provided by the tool, we predicted the clones that could efficiently bind the viral epitopes. Three months after injection, fewer TCRs were predicted to bind the antigen in the vaccine alone, whereas GLSP intervention maintained this proportion at pre-injection levels and increased the proportion of TCRs capable of binding multiple epitopes (Fig. 8H). Moreover, among the top expanded clones, two clones from TopExp.90D showed a strong affinity for the virus peptides (Fig. 8I). The two clones were mostly distributed in CD8 Te_KLRD1 and CD8 Tem_CMC1 (Fig. 8J), further emphasizing the important role of these two sub-clusters in GLSP adjuvant vaccine immunity. These findings indicate that the selection of TCR-dominant clones by GLSP may promote superior binding to antigens and more robust antiviral immune responses when compared to vaccine booster injections alone.

### Identification and functional characterization of GLSP triterpenoids as activators of IFN-α signaling

**Fig. 9:**
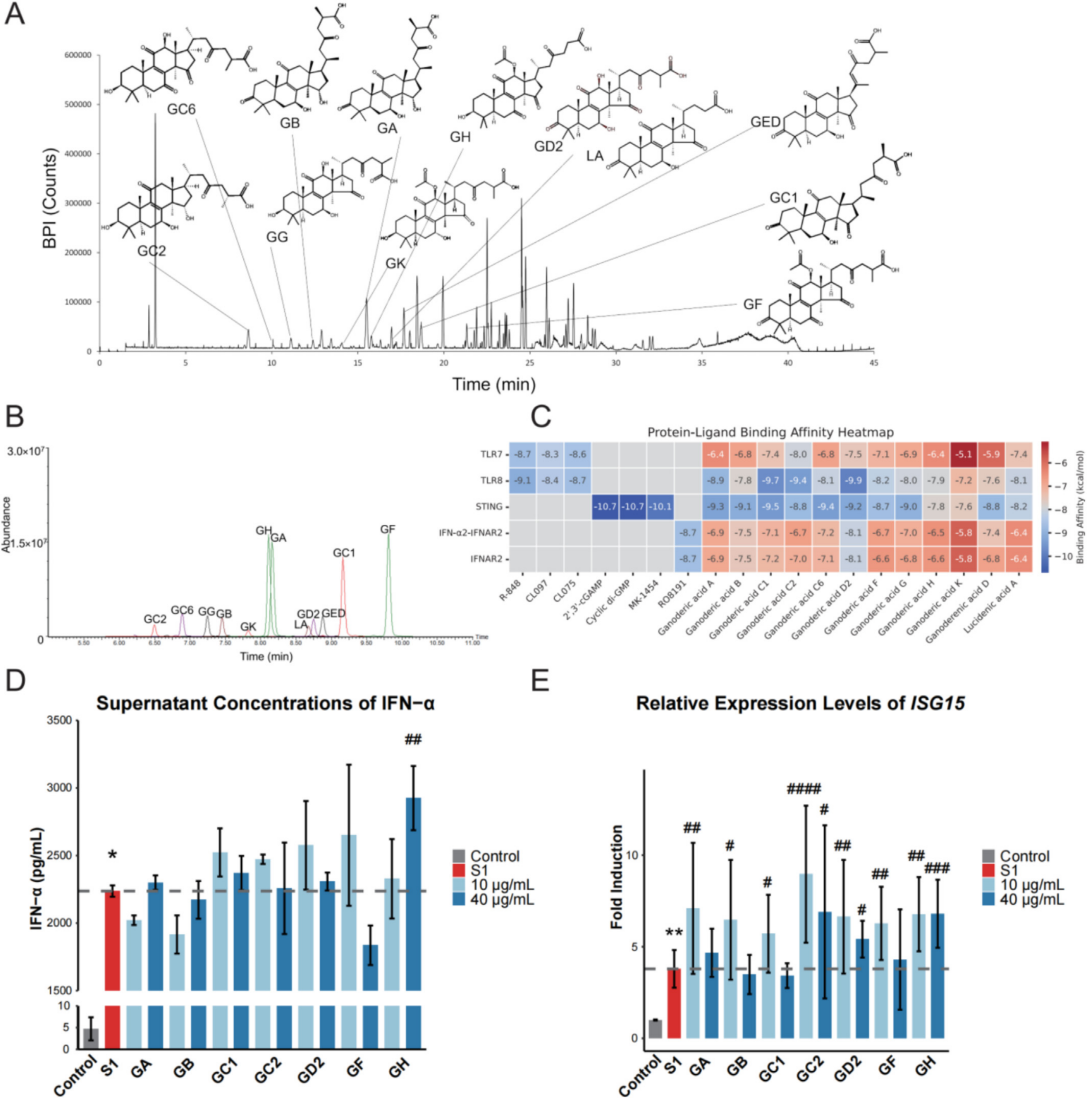
**Triterpenoids in GLSP activate key proteins in IFN-α induction and signaling. (A)** In the base peak ion (BPI) chromatogram of UPLC-QTOF-MS, 12 triterpenoids are labeled. (B) The MRM chromatogram of the triterpenoids. (C) The binding affinity of agonist and triterpenoid molecules to target proteins. (D) The concentration of IFN-α in the cell supernatant of each group. (E) The relative expression levels of *ISG15* in each group. The significant differences in (D-E) were determined by the Kruskal-Wallis test, followed by Dunn’s post-hoc test. The significance relative to the control is denoted by asterisks (*), while significance relative to the S1-stimulated alone group is denoted by hash symbols (#). The significance levels are defined as follows: * or #, *p* < 0.05; ** or ##, *p* < 0.01; *** or ###, *p* < 0.001; **** or ####, *p* < 0.0001. The error bars represent the mean values ± standard deviation (SD). S1: the recombinant 2019-nCoV S1 protein. GA: ganoderic acid A. GB: ganoderic acid B. GC1: ganoderic acid C1. GC2: ganoderic acid C2. GD2: ganoderic acid D2. GF: ganoderic acid F. GH: ganoderic acid H.

In order to identify the specific active ingredients, we performed UPLC-QTOF-MS analysis to characterize the compound composition of GLSP. As a result, a total of 63 components with a predominance of GL triterpenoids were identified (Fig. 9A, Supplementary Table S9). Among these, 12 triterpenoids were considered as major components that had higher bioavailability according to our previous pharmacokinetic studies, referred to as the blood-entering triterpenoids [57,104]. Using UPLC-MRM-MS, we further quantified the contents of the 12 triterpenoids in GLSP (Fig. 9B, Supplementary Fig. S9, and Table S10).

Afterward, we performed molecular docking to simulate the 12 reported blood-entering triterpenoids against key targets involved in IFN-α production and signaling activation, aiming to identify the components that play key roles. Among these targets, *TLR7*, *TLR8*, and *STING* recognize viral genetic material and, through signaling, stimulate downstream IFN-α secretion [105,106]. In addition, we targeted *IFNAR2* and its complex with IFN-α2 to observe the effect of triterpenoids on IFN-α response. To test whether triterpenoids act as activators with the structures of the target, we also performed the docking of known agonists with the corresponding target proteins under the same parameter settings. All agonists were successfully docked, achieving good binding scores of less than -8 kcal/mol (Fig. 9C). Most triterpenoid molecules naturally bind proteins associated with IFN-α secretion (binding energy less than -7 kcal/mol) (Fig. 9C). Affinity for *TLR8* and *STING* is particularly strong, and some molecules have binding capacities that even exceed those of known agonists. For example, the binding energies of GC1, GC2, and GD2 to *TLR8* are lower than all the agonists shown. A significant fraction of triterpenoids can also efficiently bind *IFNAR2* and the IFN-α2-*IFNAR2* complex (*IFN-α2-IFNAR2* complex: 6/12, *IFNAR2*: 5/12). Moreover, for all target proteins, there are triterpenoids capable of binding to the same residues as the agonist (Supplementary Figs. S10–S11). It suggests that they have the potential to act as novel agonists of the IFN-α signaling pathway.

To further investigate the active components responsible for IFN-α pathway induction, we assessed the effects of seven candidate triterpenoids (GA, GB, GC1, GC2, GD2, GF, and GH) on human PBMCs stimulated with the 2019-nCoV S1 protein (abbreviated as S1 in the following text). Indeed, S1 stimulation rapidly induced an acute IFN-α response, consistent with host antiviral immune activation [107]. Furthermore, administration of several triterpenoids could enhance IFN-α secretion beyond the S1 stimulation alone, potentially contributing to more effective antiviral immunity [108,109]. Specifically, a concentration of 40 μg/mL of GH induced a significant increase in IFN-α secretion compared to S1 stimulation alone. Although not statistically significant, a consistent upward trend in IFN-α production was observed with certain triterpenoids, including GC1 and GC2 at 10 μg/mL (Fig. 9D).

Furthermore, at the transcriptional level, we quantified the expression of a panel of ISGs—including *MX1*, *OAS1*, *IFI44L*, and *ISG15*—which are all critical downstream effectors in the type I IFN antiviral response [110,111]. In comparison with S1 stimulation alone, the triterpenoids generally upregulated the expression of these ISGs; however, the extent of this upregulation varies (Supplementary Fig. S12). The induction of *MX1* and *OAS1* was modest, with only GB (40 μg/mL) significantly upregulating *MX1*. In contrast, the response was markedly more pronounced for *IFI44L* and *ISG15*. While GD2 and GH at 10 μg/mL showed a strong trend of *IFI44L* induction, the most robust and significant effects were centered on *ISG15*, a core ubiquitin-like effector in the interferon response [112]. Remarkably, all seven triterpenoids significantly upregulated *ISG15* expression at the lower concentration (10 μg/mL), with GC2 exhibiting the most potent effect. The significant up-regulation was sustained at the higher concentration (40 μg/mL) only for GD2 and GH (Fig. 9E). This revealed a non-linear dose–response relationship for most compounds, where the lower dose was more effective, indicating their complex pharmacological profiles. Importantly, GH exhibited a uniquely stable ability to enhance *ISG15* expression at both concentrations. Collectively, GH emerges as the most active triterpenoid for promoting IFN-α production and downstream type I IFN signaling. In addition, GC1 and GC2 are also components with considerable potential in stimulating the IFN-α pathway, warranting further pharmacological investigation.

## Discussion

One of the main issues with the current vaccine is its declining effectiveness. Peak antibody titer levels are typically reached within a month after the COVID-19 vaccination; however, efficacy can diminish by more than 50 % within six months [113]. The decline in effectiveness occurs even more rapidly with the influenza vaccine, which may lose its efficacy within a month [[114], [115]]. Additionally, for vaccines that have previously demonstrated long-term effectiveness, such as those for rotavirus, pertussis, and mumps, the decline in efficacy has also become a significant concern [116]. Adjuvants can enhance the magnitude and duration of immune responses to vaccines. At present, the range of available vaccine adjuvants is restricted, and there is considerable uncertainty regarding their efficacy and safety [16,18]. It is postulated that natural product extracts may serve to compensate for this advantage as immune adjuvants, with GL products being a promising application in this area. In particular, extracts from sporoderm-broken GL spores are excellent at immune modulation. For instance, GL spore extracts could reduce iNOS expression and inhibit inflammation [[117], [118]], thereby potentially helping with many diseases, such as neuroinflammation and damage [[119], [120]], tumorigenesis [121], and infections [122]. Additionally, GL spore extracts are effective in preventing infections [123], thereby having the potential to be developed as immune supplements and vaccine adjuvants. Therefore, in the present study, we applied GLSP as an adjuvant to the SARS-CoV-2 vaccine booster shots to demonstrate its ability to enhance IFN-α-mediated antiviral capacity and improve vaccine efficacy.

IFN-α has been demonstrated to activate both the innate and adaptive immune systems, enhance antigen presentation, pro-inflammatory pathways, and cytokine production, and play an important role in the protection against acute viral infections [[79], [124]]. In addition, recent studies have indicated that IFN-α may have the potential to serve as a vaccine adjuvant [[125], [126], [127]]. In our study, GLSP significantly increases the serum IFN-α concentrations of individuals receiving booster shots. Then, GLSP influences the response to IFN-α through a multifaceted, multi-omics approach. In innate immunity, GLSP expanded the proportion of myeloid cells, particularly doubled the proportion of two *CD14*^+^*CD16*^-^ CM sub-clusters compared to vaccination alone, and demonstrated up-regulation of the genes related to the IFN-α response of those CM clusters. The reshaping of the myeloid cell proportion and function can lead to the regulation of immune microenvironments. For instance, the polarity shift between M1 and M2 macrophages could inhibit inflammation in wound healing [[128], [129]], and the elevation of the *CD14*^+^ monocytes and DCs proportion could promote cytokine production and immune stimulation [130]. In terms of upstream regulation, on the one hand, GLSP markedly elevated the activity of TFs that play a crucial role in regulating the IFN-α response pathway; on the other hand, signatures related to IFN-α response exhibited higher chromatin accessibility scores after drug intervention. Both infection and vaccination could induce epigenetic changes in ISGs in myeloid cells to promote long-lasting immune memory and subsequent stronger antiviral immunity [[82], [131]]. That suggests that GLSP could influence the trained immunity of monocytes and therefore enhance the resistance to the virus. In terms of transcriptomics, GLSP intervention resulted in the upregulation of IFN-stimulated genes in CMs, while simultaneously enhancing the conserved positive antiviral response signatures. Notably, the BNT162b2 vaccine, one of the most extensively deployed vaccines for SARS-CoV-2 globally, has been observed to induce the expansion of a mixed *CD14*^+^ monocyte-dendritic cell state, which is characterized by a high expression level of interferon-stimulated genes [81]. Therefore, it can be reasonably deduced that the elevation of the cell proportion of these two CM sub-clusters and the enhancement of their IFN response may be the key to GLSP assisting in the enhancement of organismal immunity.

GL extracts have been shown to promote the proliferation and activation of lymphocytes for immunomodulation, particularly by inducing *CD69*^+^ lymphocytes [[132], [133], [134]]. This was also demonstrated in our study. In comparison with the vaccine alone, GLSP tended to maintain the memory B cells and induce the naïve B clusters with high levels of *CD69* and AP-1, especially the IFN-stimulated sub-type. Interestingly, IFN-I during early infection can activate B cells and upregulate their expression of *CD69* and *CD86*, which in turn leads to a series of signaling cascades that ultimately affect B cell maturation and antibody response [[135], [136], [137], [138]]. Therefore, compared to a vaccine booster alone, GLSP can stimulate the maturation of naïve B cells, preparing them for further maturation and BCR signaling cascades through an enhanced response to IFN-α. Indeed, V-J pairs enriched in expanded clones of the GLSP intervention group were associated with a stronger IFN response. In terms of T cells, GLSP increased the proportion of activated *CD8^+^* T clusters with differentiation potential based on the vaccine booster. Those sub-clusters can play a crucial role in the cellular response to both COVID-19 infection and vaccine immunization [[139], [140]]. Among them, CD8 Tem_CMC1 and CD8 Te_KLRD1 are enriched with highly expanded TCR clones with a strong affinity to the SARS-CoV-2 antigen epitopes. Furthermore, those highly expanded TCRs were characterized by up-regulated type I IFN signaling and positive MVS. It can be concluded that GLSP is capable of enhancing the response of B cells and T cells to IFN-α stimulation, specifically through its effect on the rearrangement of the V(D)J region and the selective expansion of clones.

In GL extracts, triterpenoids have important immunoregulatory and antiviral activities [[141], [142], [143], [144]]. This study mainly focused on the 12 blood-entering triterpenoids reported in our previous pharmacokinetic studies [57,104]. Through molecular docking, we first identified several triterpenoids, such as GC1, GC2, and GH, exhibiting high affinity for key target proteins in the IFN-α pathway and sharing the same binding sites as known agonists. Crucially, our subsequent in vitro experiments confirmed that these compounds could indeed promote IFN-α secretion or upregulate the expression of IFN-α response genes (such as *ISG15*), especially the GH. Despite the identification of the potential active triterpenoids within GLSP, further investigation is required into the synergistic effects of the lead compounds within GLSP through the implementation of in vivo studies.

Another interesting finding was that GLSP induced an increased proportion of platelet-leukocyte aggregates (PLA) and their sub-types (Supplementary Fig. S13). The complex role of PLA in immune regulation has been noted. In the context of an infection, the aggregation of platelets and leukocytes has the potential to stimulate the secretion of cytokines and to facilitate the recruitment and infiltration of leukocytes into target tissues, thereby enabling the clearance of pathogens [145,146]. However, poor prognosis has also been reported with platelet-leukocyte interactions [147,148]. Moreover, an increase in PLA has been observed in recipients of the SARS-CoV-2 vaccine, which has been linked to a swift antigenic response [149]. Importantly, this phenomenon did not result in excessive immunothrombosis, as seen during infection [150]. Thus, the expansion of PLA in vaccines may indicate safe immunoprotection. This could be another way in which GLSP improves vaccine efficacy. However, the exact mechanism by which this occurs requires further exploration.

In summary, our study utilized GLSP to enhance the efficacy of SARS-CoV-2 vaccine boosters, demonstrating its potential for development as a novel vaccine adjuvant. This is a direct response to the call to embrace and rigorously validate the clinical value in traditional Chinese medicine development [151]. This provides a solution to the waning effectiveness of existing vaccines and complements the limitations on the safety and efficacy of existing immune adjuvants. Additionally, though the benefits of GL extracts in stimulating response to vaccine antigens have been discussed in published studies [28,29,70], there is still a lack of application and in-depth pharmacological mechanistic investigation of GL products in large cohorts. However, this study applied GLSP to an expanded clinical cohort and provided a detailed landscape of its influence on innate and adaptive systems in terms of transcriptional regulation, chromatin accessibility, and BCR/TCR clonal selection.

Nevertheless, there are a few issues with this study that require further refinement. It is acknowledged that several factors inherent to an individual may influence the extent of the response to a vaccine, such as the strength of innate immune signaling [[152], [153], [154], [155], [156]]. These factors may similarly limit the effects of GLSP on individuals with either low or high baseline immunity. However, the underlying mechanisms require further investigation. Additionally, the efficacy and safety of GLSP as an adjuvant for other vaccines, including those for influenza, HBV, and human tumors, still need to be demonstrated.

## Conclusion

In this study, a natural medicine product, GLSP, was administered to recipients of the SARS-CoV-2 vaccine, resulting in an enhancement of vaccine efficacy. Specifically, the effect of GLSP on vaccine immunity has been observed from single-cell multi-omics. First, GLSP drives a systemic type I IFN response, significantly increasing serum IFN-α levels in vaccine recipients, which is indicative of its regulatory role in IFN-α pathways. Second, GLSP reprograms the innate immune system at a fundamental level by increasing the proportion of myeloid cells, particularly classical monocytes. This is also achieved by boosting the activity of key transcription factors and increasing the accessibility of IFN-α response elements in the chromatin of these cells. Third, GLSP shapes a more effective adaptive immune response by altering the clonal selection of B and T cell receptors, favoring repertoires with improved IFN signaling and antiviral capacity. Fourth, through molecular docking and subsequent in vitro validation, we identified the triterpenoid GH as a key active component within GLSP responsible for activating the IFN-α induction and signaling. Taken together, GLSP potentiated IFN-α-mediated antiviral defenses through multimodal mechanisms, highlighting its potential as a novel vaccine adjuvant and antiviral agent.

## Declaration of competing interest

The authors declare that they have no known competing financial interests or personal relationships that could have appeared to influence the work reported in this paper.
