# Supplementary material for: Ganoderma lucidum spore powder enhances IFN-α-mediated antiviral capacity of COVID-19 vaccine boosters revealed by single-cell multi-omics sequencing
Source: J Adv Res. 2025 Oct 12;85:737–59. doi: 10.1016/j.jare.2025.10.014 (PMC13316359; doi:10.1016/j.jare.2025.10.014)
Supplement: Supplementary Data 1 [file mmc1.docx]

**Supplemental Information**

***Ganoderma Lucidum* Spore Powder Enhances IFN-α-Mediated Antiviral Capacity of COVID-19 Vaccine Boosters Revealed by Single-Cell Multi-Omics Sequencing**


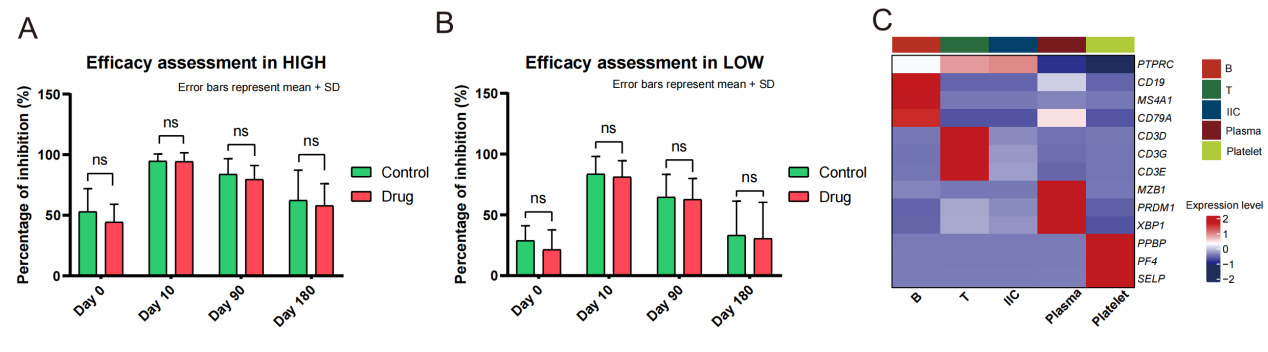


**Figure S 1 Efficacy Evaluation of *GLSP* and Markers of Cell Types**

(A-B) Effect of GLSP on the percentage of inhibition in HIGH and LOW populations at different time points. (C) Heatmap of markers in different cell types. Significant differences in (A-B) were determined by the Wilcoxon test (ns - *p* > 0.05). The error bars represent the standard deviation (SD), and the horizontal bars show the mean values of the percentage of inhibition. HIGH/LOW: the population with high/low baseline immunity. HIGH: the population with high baseline immunity. LOW: the population with low baseline immunity.


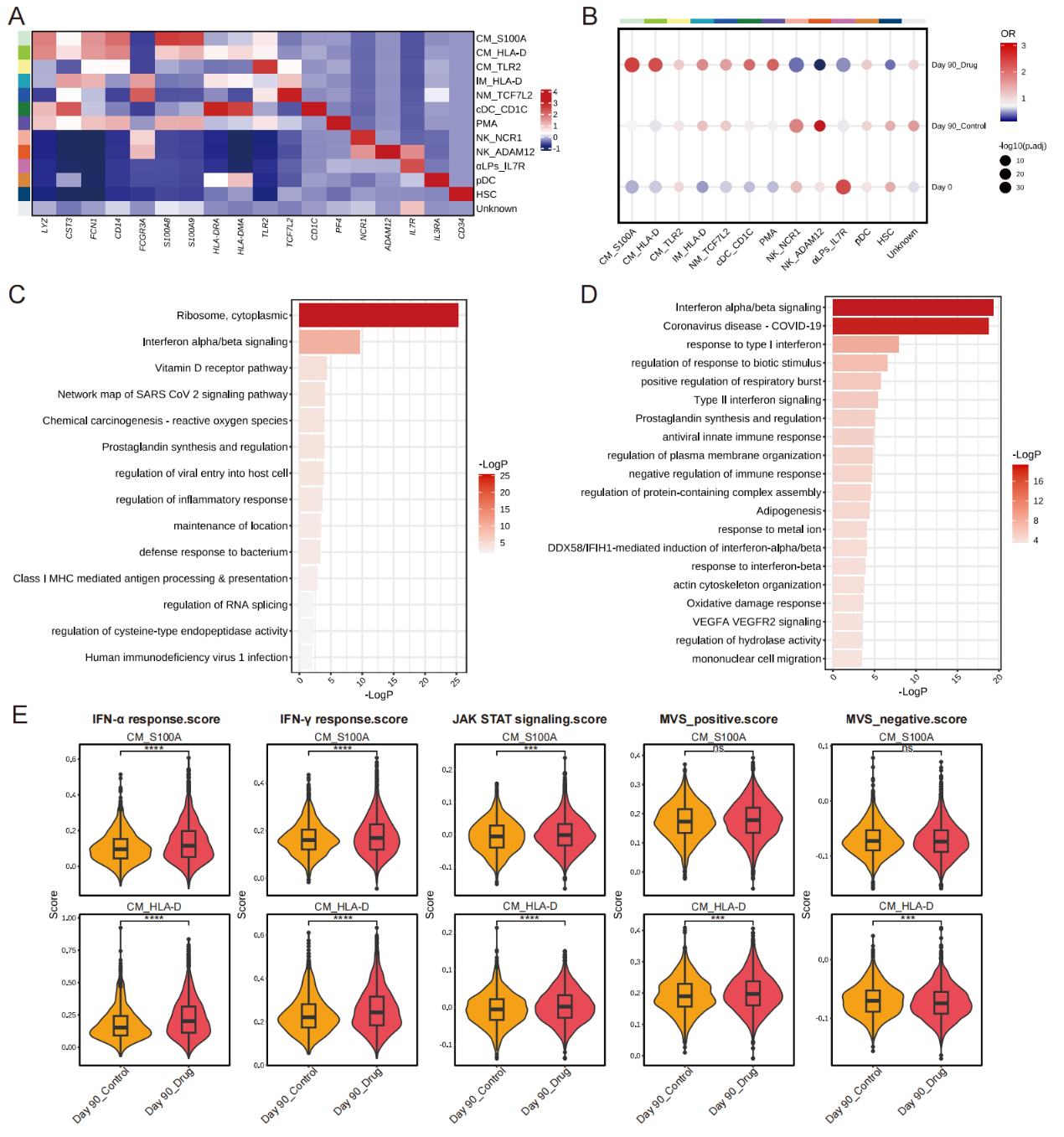


**Figure S 2 Characteristics of IIC Clusters and Comparison Among Conditions**

(A) Heatmap of markers in different sub-clusters. (B) OR of all sub-clusters in different time points and groups. (C-D) The enriched summary terms of the GLSP-intervention group versus the control group 90 days post-vaccination of CM_S100A (C) and CM_HLA-D (D). (E) Scores of IFN-α response, IFN-γ response, JAK-STAT signaling, positive and negative MVS across conditions. Significant differences in (E) were determined by the Wilcoxon test (**p* < 0.05, ***p* < 0.01, ****p* < 0.001, *****p* < 0.0001, ns - *p* > 0.05). Day 0: pre-injection; Day 90_Control: the control group 90 days post-injection; Day 90_Drug: the GLSP intervention group 90 days post-injection.


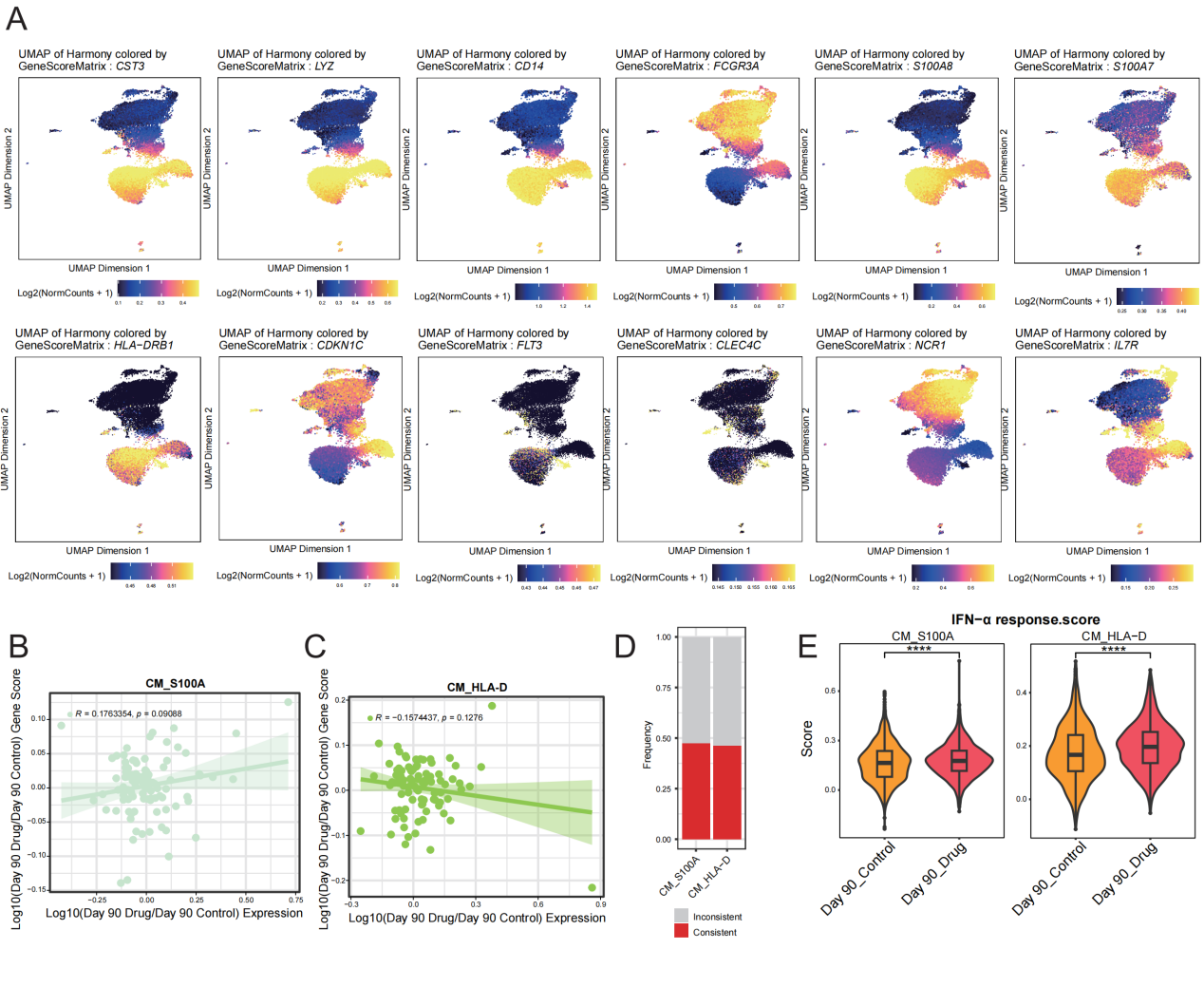


**Figure S 3 Markers of IIC Sub-clusters in scATAC-seq Profile and Epigenetic Regulation of the IFN-α Response**

(A) The markers of IIC sub-clusters in scATAC-seq data. (B-C) The correlation of the chromatin accessibility changes and the expression changes of the IFN-α response-related genes of CM_S100A (B) and CM_HLA-D (C). (D) The proportion of genes that share the same changing trend of chromatin accessibility and expression in all IFN-α response-related genes. (E) Scores of IFN-α response based on gene scores in the control group and the GLSP intervention group. The Wilcoxon test determined significant differences in (E) (*****p* < 0.0001). Day 90_Control: the control group 90 days post-injection; Day 90_Drug: the GLSP intervention group 90 days post-injection.


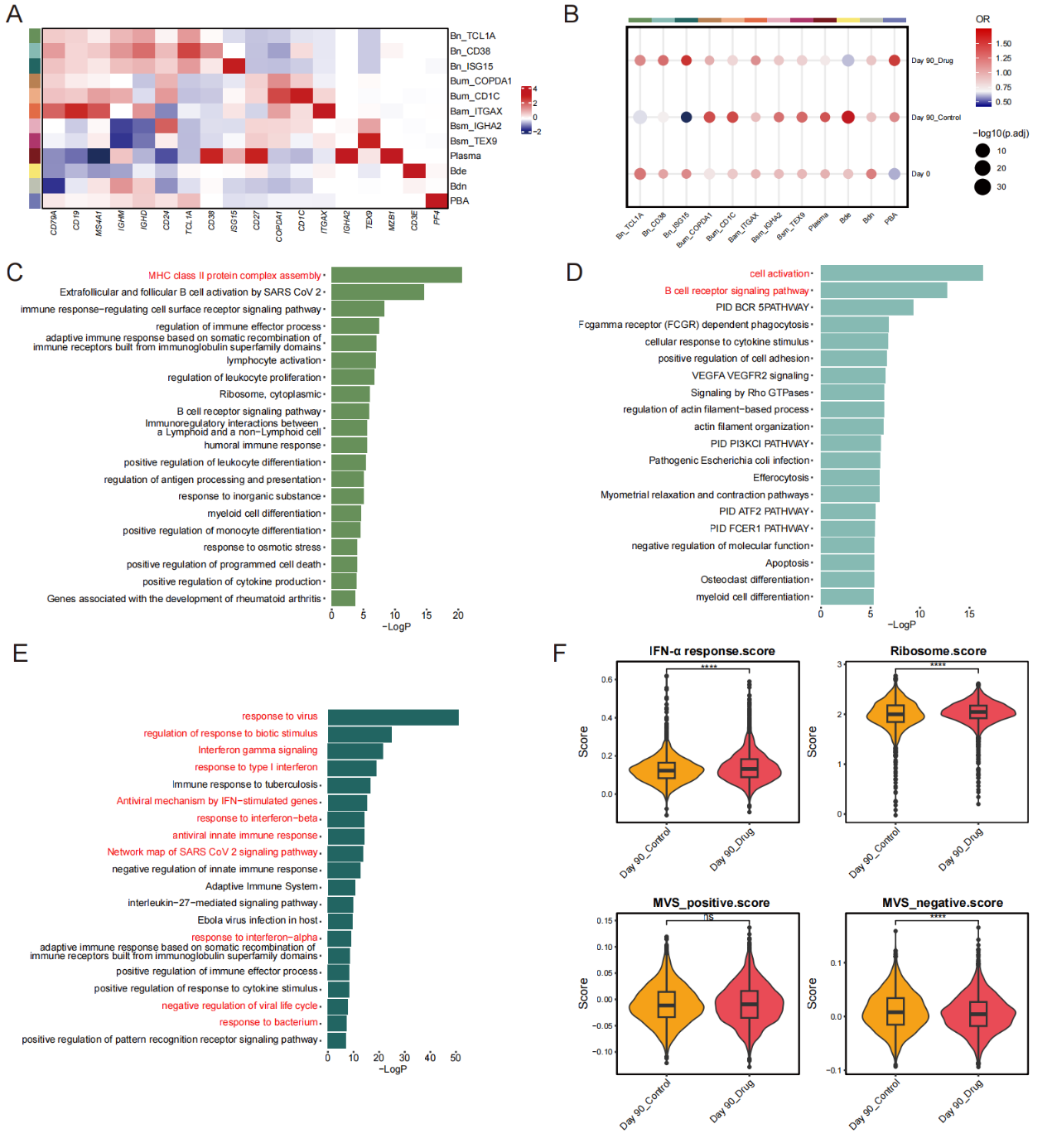


**Figure S 4 Characteristics of B and Plasma Cell Clusters and Comparison Among Conditions**

(A) Heatmap of markers in different sub-clusters. (B) OR of all sub-clusters in different time points and groups. (C-E) The enriched summary terms of up-regulated genes of Bn_TCL1A (C), Bn_CD38 (D), and Bn_ISG15 (E) in B and plasma cells. (F) Scores of IFN-α response, ribosome, positive and negative MVS across conditions. Significant differences in (E) were determined by the Wilcoxon test (**p* < 0.05, ***p* < 0.01, ****p* < 0.001, *****p* < 0.0001, ns - *p* > 0.05). Day 0: pre-injection; Day 90_Control: the control group 90 days post-injection; Day 90_Drug: the GLSP intervention group 90 days post-injection.


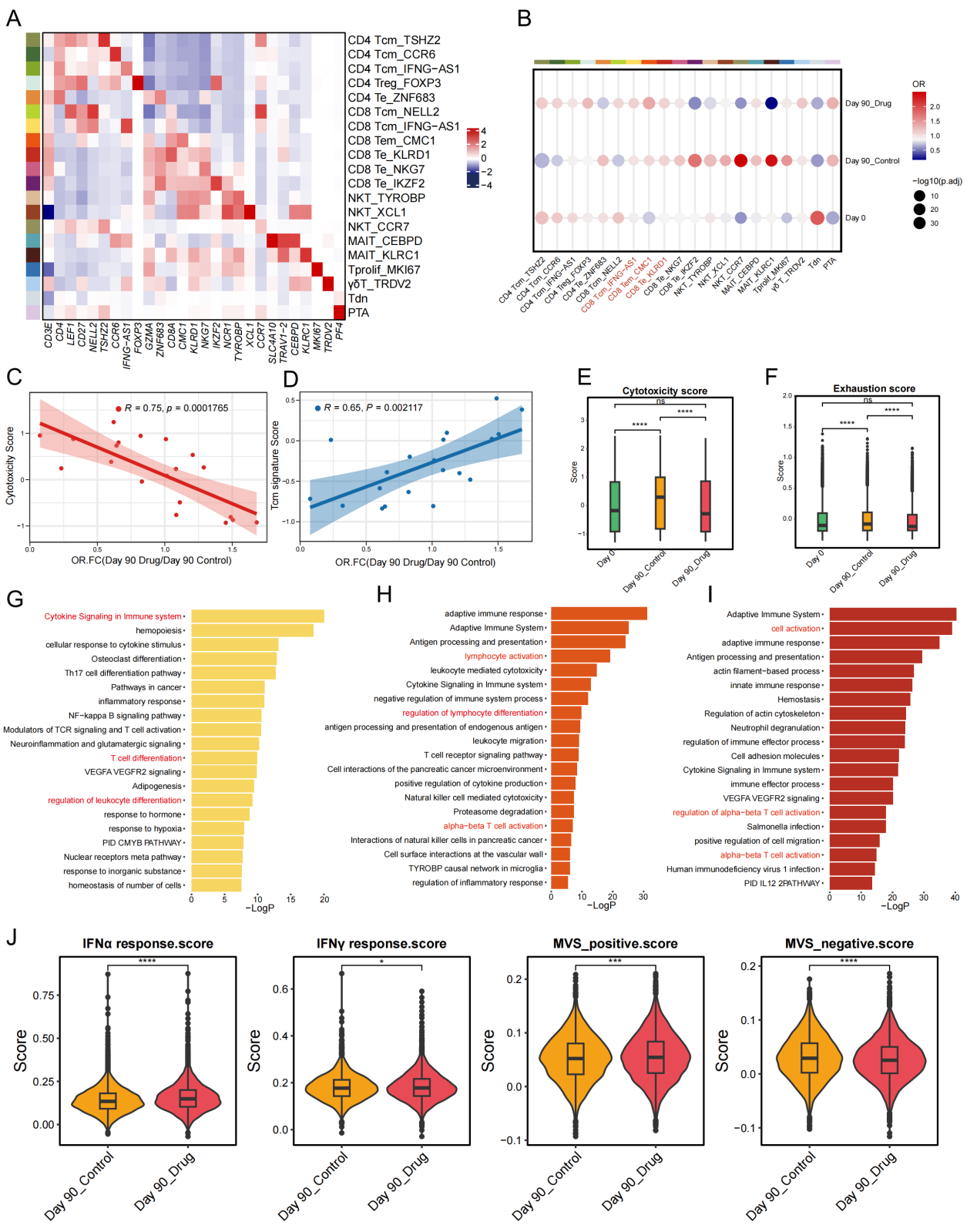


**Figure S 5 Characteristics of T Cell Clusters and Comparison Among Conditions**

(A) Heatmap of markers in different sub-clusters. (B) OR of all sub-clusters in different time points and groups. (C-D) The correlation between Cytotoxicity scores (C) and Tcm signature scores (D) with OR fold changes of the GLSP-intervention group versus the control group 90 days post-vaccination. (E-F) Cytotoxicity score (F) and Exhaustion score (G) across conditions. (H-J) The enriched summary terms of up-regulated genes of CD8 Tcm_IFNG-AS1 (H), CD8 Tem_CMC1 (I), and CD8 Te_KLRD1 (J) in T cells. (K) Scores of IFN-α response, IFN-γ response, positive and negative MVS in the GLSP-intervention group and the control group 90 days post-vaccination. Significant differences in (E-F) and (K) were determined by the Wilcoxon test (**p* < 0.05, ***p* < 0.01, ****p* < 0.001, *****p* < 0.0001, ns - *p* > 0.05). Day 0: pre-injection; Day 90_Control: the control group 90 days post-injection; Day 90_Drug: the GLSP intervention group 90 days post-injection.


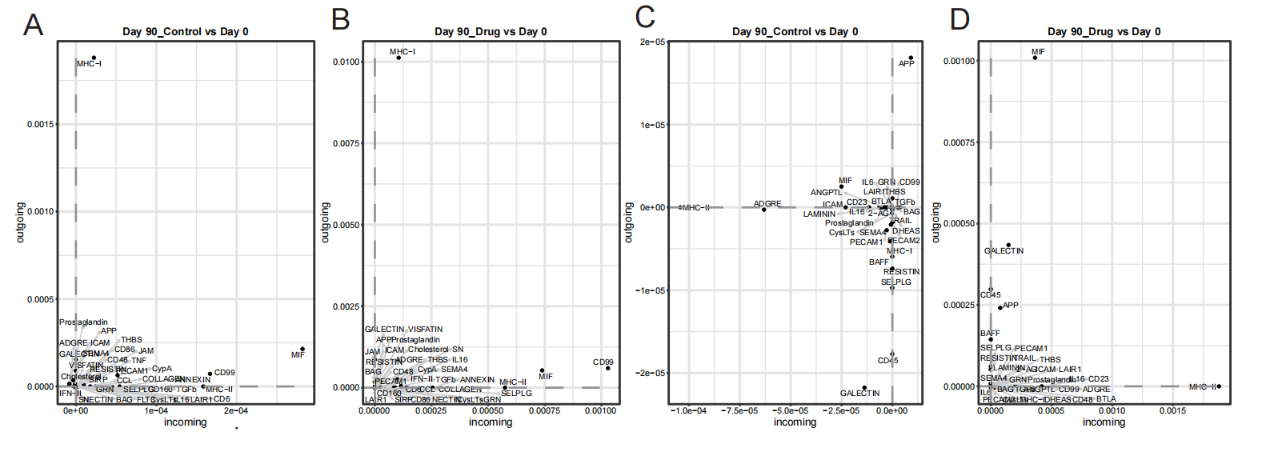


**Figure S 6 Differential Strength of Signaling Pathways between Different Groups Post-vaccination versus Pre-vaccination**

(A-B) The differential interaction strength in the control group (A) or the GLSP-intervention group (B) post-vaccination versus pre-vaccination. CM_S100A and CM_HLA-D were set as senders while CD8 Tem_CMC1 and CD8 Te_KLRD1 were set as receptors. (C-D) The differential interaction strength in the control group (C) or the GLSP-intervention group (D) post-vaccination versus pre-vaccination. CM_S100A and CM_HLA-D were set as senders while Bn_TCL1A was set as receptors. Day 0: pre-injection; Day 90_Control: the control group 90 days post-injection; Day 90_Drug: the GLSP intervention group 90 days post-injection.


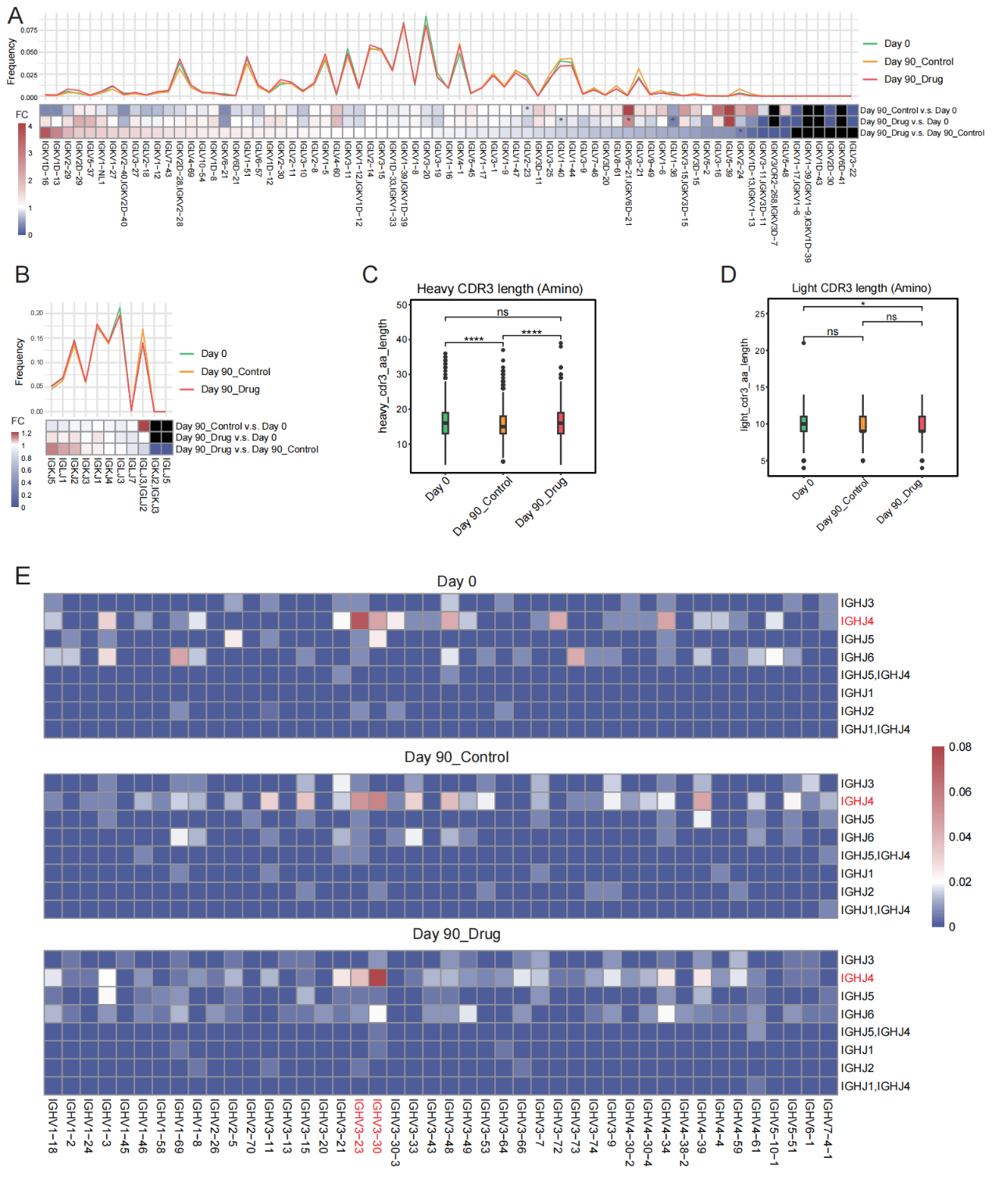
**Figure S 7 The BCR V-J Usages and CDR3 Length**

(A) Usage frequency and difference across conditions of V genes of light chain. (B) Usage frequency and difference across conditions of J genes of light chain. (C) CDR3 amino length of heavy chain across conditions. (D) CDR3 amino length of light chain across conditions. (E) The frequency of all the V-J pairs in expanded clones in all conditions. Significant differences in (A-D) were determined by the Wilcoxon test (**p* < 0.05, ***p* < 0.01, ****p* < 0.001, *****p* < 0.0001, ns - *p* > 0.05). Day 0: pre-injection; Day 90_Control: the control group 90 days post-injection; Day 90_Drug: the GLSP intervention group 90 days post-injection.


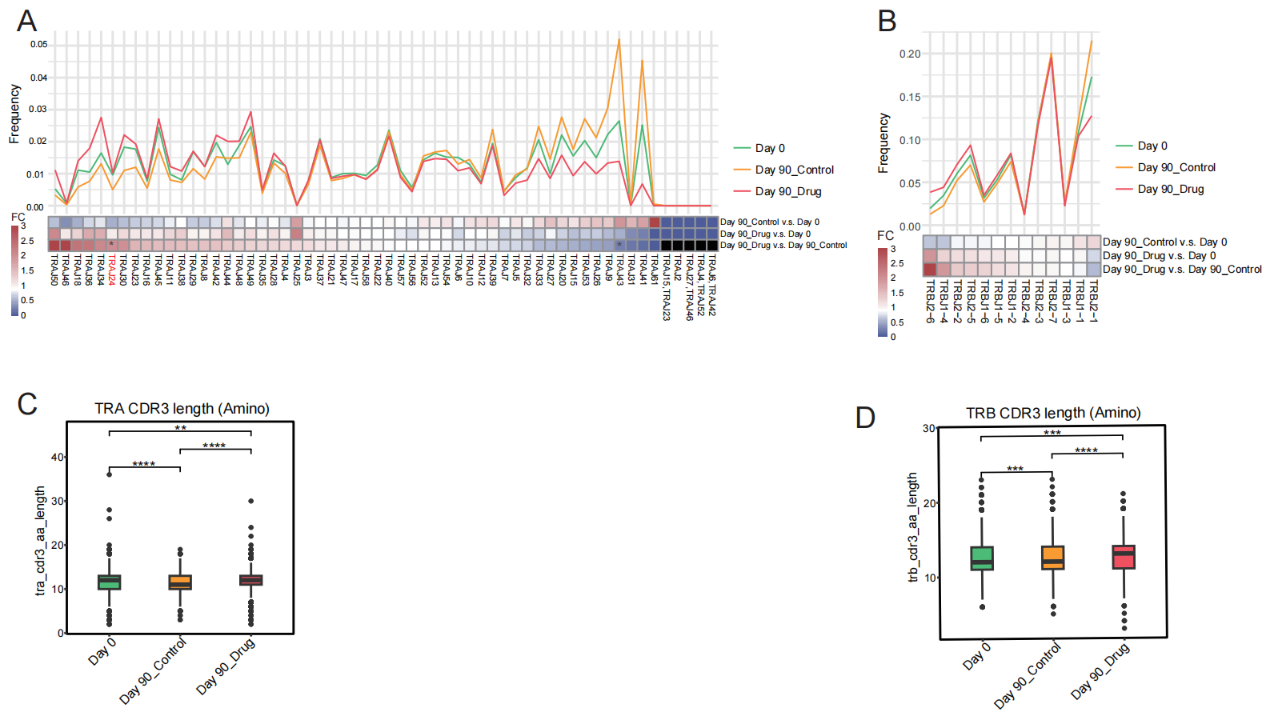


**Figure S 8 The TCR J Gene Usage and CDR3 Length**

(A) Usage frequency and difference across conditions of TRAJ genes. (B) Usage frequency and difference across conditions of TRBJ genes. (C) CDR3 amino length of α chain across conditions. (D) CDR3 amino length of β chain across conditions. Significant differences were determined by the Wilcoxon test (**p* < 0.05, ***p* < 0.01, ****p* < 0.001, *****p* < 0.0001, ns - *p* > 0.05). Day 0: pre-injection; Day 90_Control: the control group 90 days post-injection; Day 90_Drug: the GLSP intervention group 90 days post-injection.


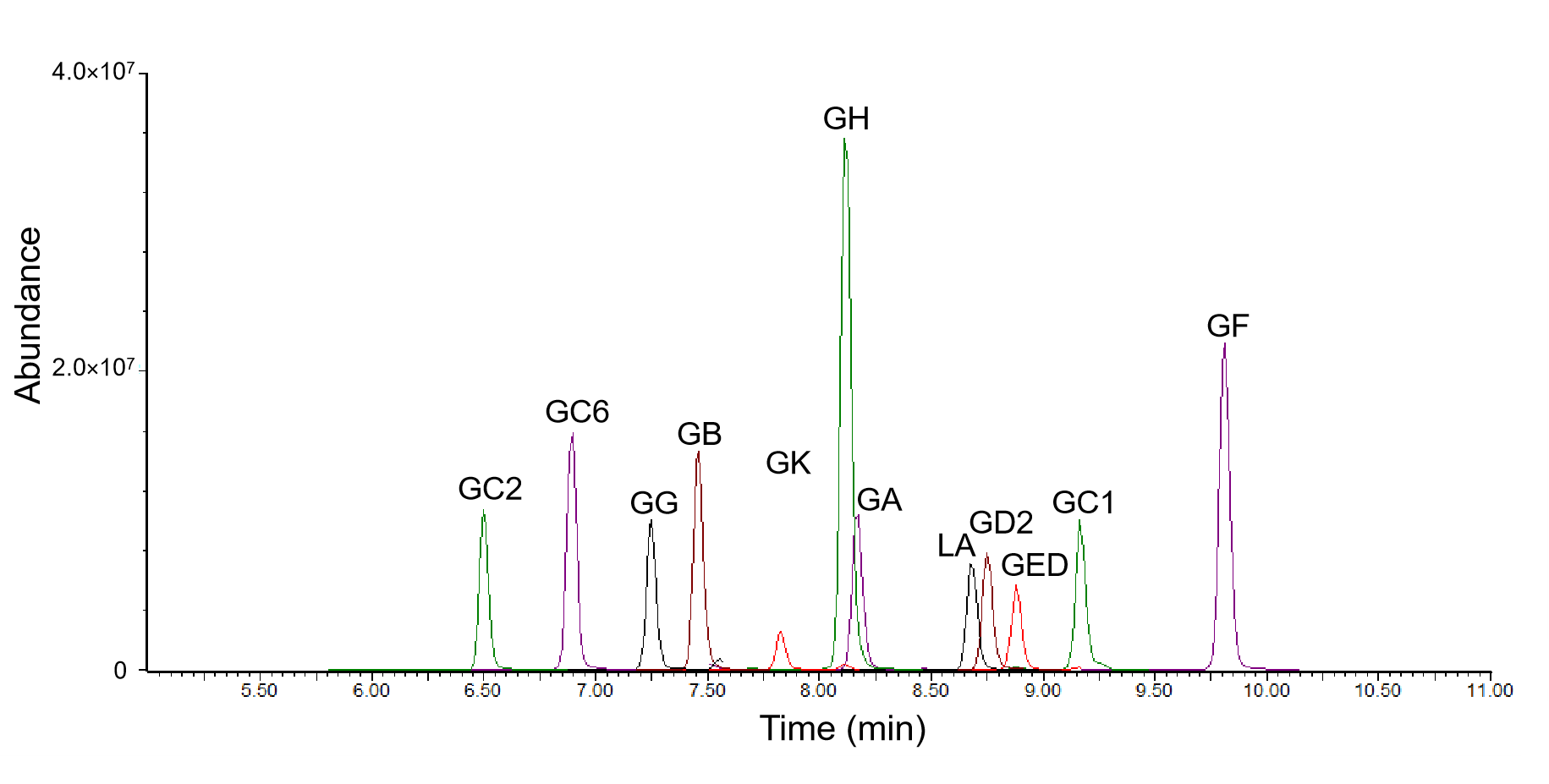


**Figure S 9 The Standard MRM Chromatogram of the Triterpenoids**

GA: ganoderic acid A. GB: ganoderic acid B. GC1: ganoderic acid C1. GC2: ganoderic acid C2. GC6: ganoderic acid C6. GD2: ganoderic acid D2. GF: ganoderic acid F. GG: ganoderic acid G. GH: ganoderic acid H. GK: ganoderic acid K. GED: ganoderenic acid D. LA: Lucidenic acid A.


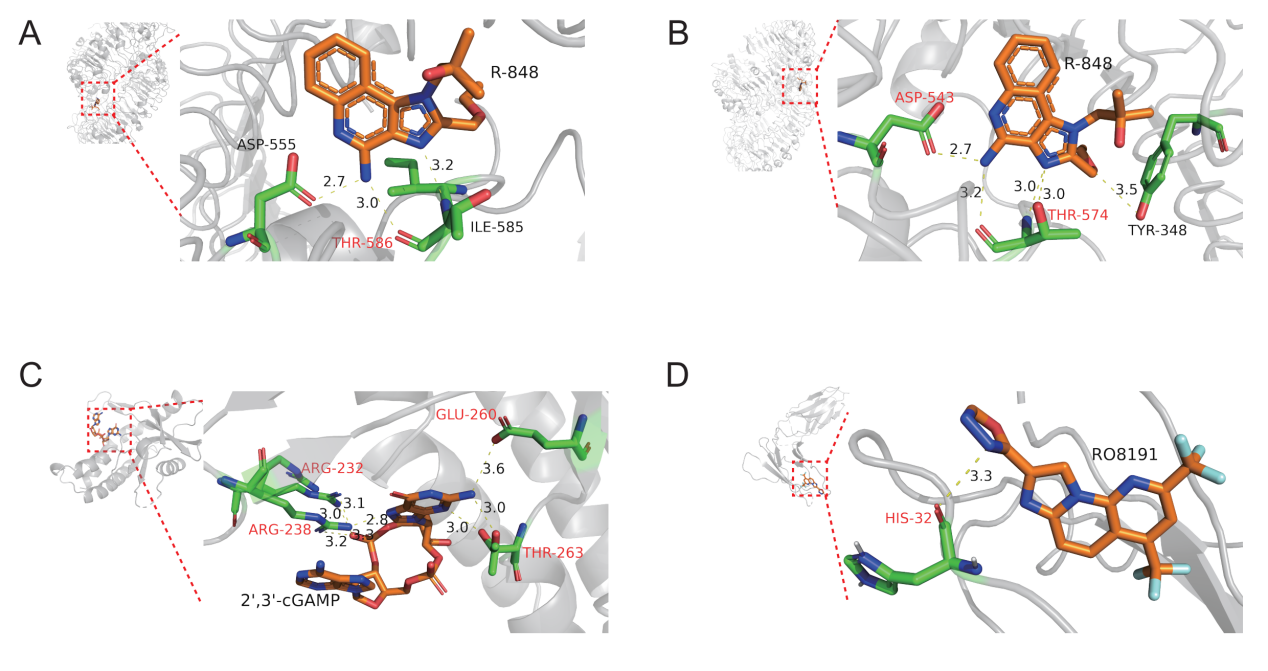


**Figure S 10 The Binding Sites of Known Agonists**

The binding sites for the target proteins TLR7 (A), TLR8 (B), STING (C), and IFNAR2 (D) with their known agonists R-848, 2’, 3’-cGAMP and RO8191. *TLR7*: Toll-like receptor 8. *TLR8*: Toll-like receptor 8. *STING*: stimulator of interferon genes. *IFNAR2*: IFN-α/β receptor 2.

**
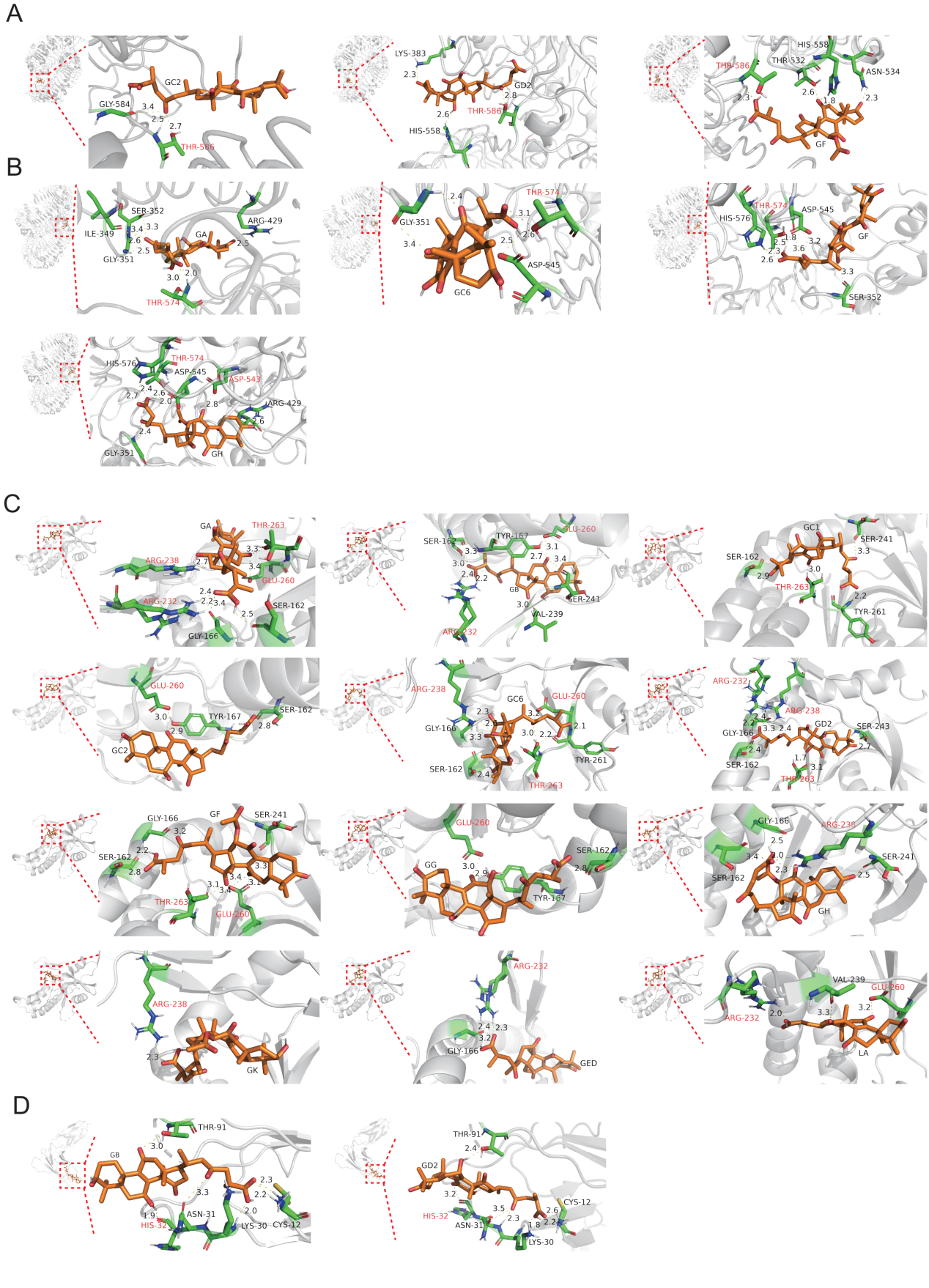
Figure S 11 Triterpenoids That Bind The Same Residues as Known Agonists at the Corresponding Targets**

The binding sites for the target proteins TLR7 (A), TLR8 (B), STING (C), and IFNAR2 (D) with triterpenoids that bind the same sites to the corresponding known agonists. GA: ganoderic acid A. GB: ganoderic acid B. GC1: ganoderic acid C1. GC2: ganoderic acid C2. GC6: ganoderic acid C6. GD2: ganoderic acid D2. GF: ganoderic acid F. GG: ganoderic acid G. GH: ganoderic acid H. GK: ganoderic acid K. GED: ganoderenic acid D. LA: Lucidenic acid A. *TLR7*: Toll-like receptor 8. *TLR8*: Toll-like receptor 8. *STING*: stimulator of interferon genes. *IFNAR2*: IFN-α/β receptor 2.


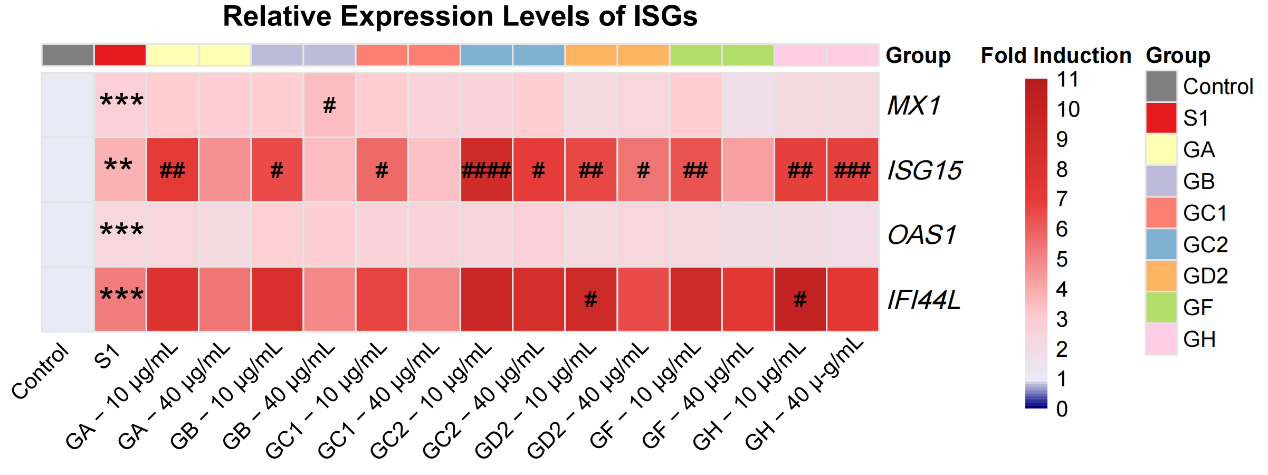


**Figure S 12 The Relative Expression Levels of Key Genes for IFN-α Induction and Signaling**

The significant differences were determined by the Kruskal-Wallis test, followed by the Dunn's post-hoc test. The significance relative to the control is denoted by asterisks (*), while significance relative to the S1-stimulated group is denoted by hash symbols (#). The significance levels are defined as follows: * or #, *p* < 0.05; ** or ##, *p* < 0.01; *** or ###, *p* < 0.001; **** or ####, *p* < 0.0001. S1: the recombinant 2019-nCoV S1 protein. GA: ganoderic acid A. GB: ganoderic acid B. GC1: ganoderic acid C1. GC2: ganoderic acid C2. GD2: ganoderic acid D2. GF: ganoderic acid F. GH: ganoderic acid H.


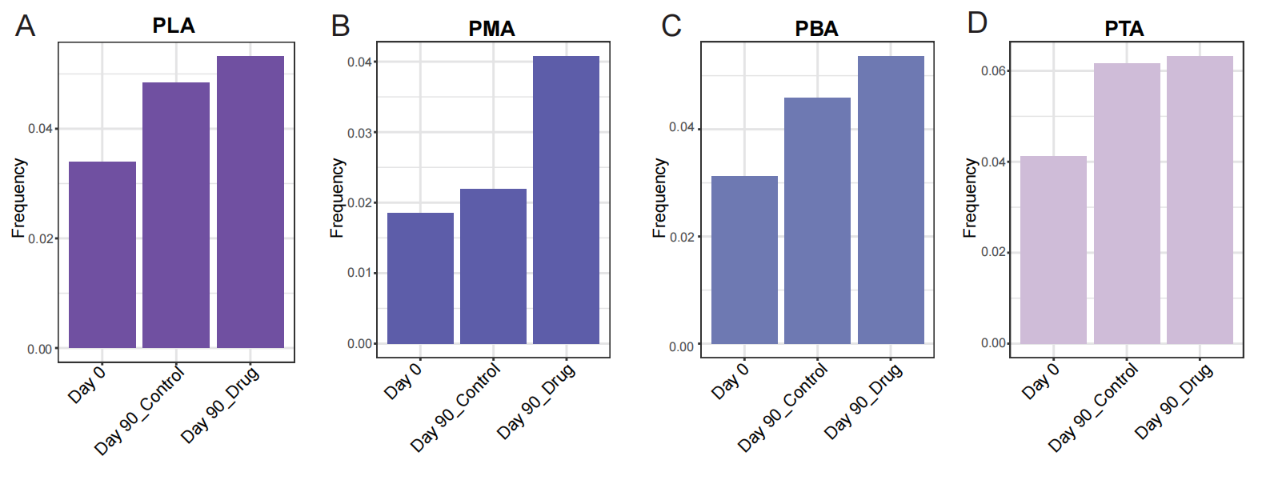


**Figure S 13 The Frequency of PLA Clusters across Conditions**

(A) The frequency of all PLA cells in PBMCs at different time points and groups. (B) The frequency of PMAs in innate immune cells at different time points and groups. (C) The frequency of PBAs in B and plasma cells at different time points and groups. (D) The frequency of PTAs in T cells at different time points and groups. PLA: platelet-leukocyte aggregates; PMA: platelet-monocyte aggregates; PBA: platelet-B aggregates; PTA: platelet-T aggregates. Day 0: pre-injection; Day 90_Control: the control group 90 days post-injection; Day 90_Drug: the GLSP intervention group 90 days post-injection.

**Table S1 The Information of 195 Volunteers**

| Number | Group^1^ | Gender | Age | Hight (cm) | Weight (kg) | Percentage of inhibition | | | | SARS-CoV-2 S-specific IgG (OD) | Population of baseline immunity^2^ |
| --- | --- | --- | --- | --- | --- | --- | --- | --- | --- | --- | --- |
|  |  |  |  |  |  | Day 0 | Day 10 | Day 90 | Day 180 |  |  |
| WY102 | Control | F | 55 | 163 | 52 | 51.61 | 95.08 | 80.08 | 65.58 | 3.65 | HIGH |
| WY193 | Drug | F | 43 | 156 | 50 | 82.66 | 99.10 | 96.81 | 92.98 | 3.57 | HIGH |
| WY088 | Control | F | 51 | 162 | 55 | 79.59 | 98.64 | 95.85 | 88.11 | 3.56 | HIGH |
| WY188 | Control | F | 43 | 160 | 50 | 77.14 | 98.52 | 78.22 | 73.05 | 3.40 | HIGH |
| WY185 | Control | F | 48 | 162 | 50 | 76.95 | 98.20 | 85.69 | 72.37 | 3.37 | HIGH |
| WY123 | Drug | F | 46 | 164 | 59 | 75.40 | 80.53 | 69.31 | 30.18 | 3.36 | HIGH |
| WY176 | Control | F | 47 | 163 | 75 | 87.10 | 94.61 | 96.71 | 93.97 | 3.36 | HIGH |
| WY100 | Control | F | 47 | 154 | 55 | 72.23 | 99.46 | 84.38 | 64.99 | 3.35 | HIGH |
| WY073 | Control | F | 54 | 150 | 48 | 61.88 | 99.64 | 97.20 | 85.53 | 3.25 | HIGH |
| WY127 | Control | F | 59 | 150 | 64 | 31.88 | 99.04 | 83.00 | 62.60 | 3.24 | HIGH |
| WY005 | Control | F | 53 | 163 | 71 | 33.75 | 99.54 | 84.74 | 41.63 | 3.12 | HIGH |
| WY192 | Drug | F | 41 | 165 | 59 | 47.58 | 65.67 | 51.90 | 61.29 | 3.05 | HIGH |
| WY037 | Drug | F | 43 | 157 | 60 | 73.72 | 99.15 | 82.26 | 38.66 | 3.03 | HIGH |
| WY157 | Drug | M | 47 | 169 | 63 | 47.45 | 96.24 | 87.50 | 56.63 | 2.75 | HIGH |
| WY167 | Control | F | 54 | 155 | 63 | 37.74 | 99.11 | 84.36 | 57.59 | 2.72 | HIGH |
| WY050 | Control | F | 46 | 156 | 57 | 72.59 | 92.96 | 91.27 | 79.24 | 2.64 | HIGH |
| WY092 | Drug | F | 49 | 150 | 48 | 44.77 | 96.90 | 74.24 | 59.56 | 2.60 | HIGH |
| WY034 | Drug | M | 58 | 168 | 67 | 43.21 | 85.17 | 95.95 | 71.84 | 2.60 | HIGH |
| WY153 | Drug | F | 46 | 160 | 73 | 40.32 | 98.83 | 95.57 | 78.51 | 2.58 | HIGH |
| WY172 | Control | F | 55 | 150 | 60 | 55.17 | 98.60 | 83.67 | 51.68 | 2.53 | HIGH |
| WY054 | Control | F | 57 | 161 | 51 | 65.62 | 98.74 | 66.55 | 31.99 | 2.52 | HIGH |
| WY191 | Drug | F | 45 | 160 | 55 | 36.16 | 99.32 | 68.06 | 57.53 | 2.52 | HIGH |
| WY134 | Control | F | 51 | 160 | 57 | 39.23 | 82.73 | 85.94 | 70.48 | 2.51 | HIGH |
| WY033 | Control | F | 44 | 160 | 55 | 45.84 | 97.64 | 81.49 | 49.76 | 2.49 | HIGH |
| WY075 | Drug | F | 57 | 160 | 75 | 38.45 | 94.79 | 70.38 | 52.55 | 2.49 | HIGH |
| WY174 | Control | F | 47 | 155 | 50 | 40.25 | 99.19 | 98.36 | 97.51 | 2.46 | HIGH |
| WY084 | Drug | F | 53 | 158 | 60 | 41.04 | 97.80 | 77.77 | 57.14 | 2.46 | HIGH |
| WY096 | Drug | F | 48 | 158 | 56 | 44.10 | 94.28 | 60.24 | 36.12 | 2.46 | HIGH |
| WY022 | Control | F | 48 | 162 | 57 | 45.76 | 94.54 | 84.70 | 40.23 | 2.41 | HIGH |
| WY001 | Control | M | 41 | 168 | 67 | 38.65 | 93.69 | 90.89 | 45.24 | 2.35 | HIGH |
| WY081 | Control | F | 50 | 158 | 54 | 37.61 | 89.88 | 63.78 | 43.97 | 2.34 | HIGH |
| WY025 | Drug | M | 42 | 170 | 70 | 37.11 | 95.85 | 96.95 | 71.30 | 2.29 | HIGH |
| WY194 | Drug | F | 50 | 165 | 56 | 62.66 | 87.35 | 69.78 | 73.10 | 2.27 | HIGH |
| WY125 | Control | F | 43 | 157 | 60 | 25.05 | 97.93 | 70.38 | 52.94 | 2.25 | HIGH |
| WY143 | Control | M | 48 | 170 | 71 | 34.85 | 91.75 | 75.78 | 38.40 | 2.23 | HIGH |
| WY053 | Drug | F | 59 | 151 | 52 | 69.21 | 95.20 | 88.00 | 75.99 | 2.14 | HIGH |
| WY186 | Control | M | 44 | 168 | 70 | 52.91 | 98.36 | 88.25 | 82.91 | 2.13 | HIGH |
| WY094 | Control | F | 57 | 157 | 62 | 20.22 | 99.05 | 94.91 | 87.90 | 2.11 | HIGH |
| WY020 | Control | M | 55 | 170 | 74 | 29.30 | 90.71 | 91.19 | 56.53 | 2.06 | HIGH |
| WY036 | Drug | F | 45 | 160 | 65 | 45.37 | 94.93 | 77.14 | 26.21 | 2.04 | HIGH |
| WY038 | Control | M | 53 | 169 | 90 | 30.05 | 99.30 | 76.84 | 27.47 | 2.04 | HIGH |
| WY184 | Drug | F | 52 | 154.5 | 52.5 | 32.81 | 88.69 | 71.38 | 57.08 | 1.98 | HIGH |
| WY151 | Drug | F | 43 | 158 | 58 | 44.61 | 99.09 | 94.25 | 79.30 | 1.97 | HIGH |
| WY043 | Drug | F | 47 | 158 | 55 | 45.77 | 91.66 | 81.71 | 64.02 | 1.88 | HIGH |
| WY041 | Drug | F | 44 | 160 | 54 | 22.97 | 88.60 | 65.89 | 9.06 | 1.85 | HIGH |
| WY182 | Control | M | 40 | 164 | 64 | 43.33 | 80.48 | 51.61 | 44.11 | 1.75 | HIGH |
| WY040 | Drug | M | 45 | 165.5 | 78 | 41.02 | 92.25 | 71.13 | 27.83 | 1.74 | HIGH |
| WY049 | Control | M | 49 | 172 | 67 | 60.35 | 96.69 | 95.91 | 89.40 | 1.71 | HIGH |
| WY047 | Drug | F | 55 | 150 | 60 | 34.70 | 98.34 | 71.92 | 30.51 | 1.70 | HIGH |
| WY067 | Drug | F | 46 | 150 | 49 | 24.12 | 99.37 | 99.77 | 98.79 | 1.69 | HIGH |
| WY189 | Control | M | 53 | 170 | 63 | 62.17 | 78.20 | 78.43 | 61.45 | 1.67 | MIDDLE |
| WY196 | Drug | F | 50 | 166 | 56 | 41.33 | 94.70 | 62.51 | 42.70 | 1.66 | MIDDLE |
| WY056 | Control | M | 59 | 166 | 78 | 16.95 | 91.54 | 62.18 | 20.74 | 1.65 | MIDDLE |
| WY080 | Drug | F | 56 | 163 | 59 | 36.22 | 95.15 | 84.61 | 77.02 | 1.61 | MIDDLE |
| WY004 | Control | F | 51 | 164 | 65 | 95.39 | 90.76 | 89.07 | 64.58 | 1.60 | MIDDLE |
| WY152 | Drug | M | 42 | 174 | 70 | 62.27 | 87.30 | 71.26 | 30.09 | 1.57 | MIDDLE |
| WY158 | Control | F | 42 | 163 | 58 | 42.31 | 98.83 | 96.69 | 71.15 | 1.56 | MIDDLE |
| WY065 | Control | F | 59 | 156 | 60 | 49.56 | 80.88 | 62.76 | 42.93 | 1.56 | MIDDLE |
| WY112 | Control | F | 42 | 153 | 53 | 92.09 | 90.47 | 64.90 | 29.70 | 1.56 | MIDDLE |
| WY061 | Drug | M | 46 | 172 | 75 | 24.11 | 98.28 | 91.06 | 71.64 | 1.55 | MIDDLE |
| WY074 | Control | F | 56 | 163 | 58 | 12.04 | 92.10 | 60.35 | 32.01 | 1.52 | MIDDLE |
| WY024 | Control | F | 46 | 160 | 55 | 49.56 | 98.22 | 98.31 | 94.42 | 1.51 | MIDDLE |
| WY137 | Control | F | 57 | 168 | 67 | 32.90 | 72.50 | 50.80 | -9.70 | 1.51 | MIDDLE |
| WY042 | Drug | F | 46 | 159 | 51.5 | 25.18 | 98.89 | 97.21 | 94.58 | 1.50 | MIDDLE |
| WY197 | Drug | M | 59 | 167 | 75 | 35.75 | 97.06 | 89.11 | 77.00 | 1.46 | MIDDLE |
| WY060 | Control | F | 43 | 155 | 50 | 44.42 | 90.88 | 66.55 | 47.75 | 1.35 | MIDDLE |
| WY142 | Control | F | 54 | 163 | 56 | 38.90 | 98.50 | 89.97 | 78.67 | 1.32 | MIDDLE |
| WY159 | Drug | F | 52 | 166 | 63 | 40.32 | 97.80 | 92.65 | 78.70 | 1.28 | MIDDLE |
| WY015 | Control | M | 58 | 172 | 82 | 29.30 | 66.83 | 53.97 | 21.60 | 1.23 | MIDDLE |
| WY177 | Control | F | 48 | 162 | 59 | 35.48 | 84.38 | 64.23 | 36.01 | 1.22 | MIDDLE |
| WY145 | Control | F | 53 | 169 | 58 | 35.74 | 98.95 | 89.08 | 70.55 | 1.21 | MIDDLE |
| WY070 | Drug | M | 54 | 164 | 69 | 39.26 | 81.85 | 72.03 | 61.82 | 1.20 | MIDDLE |
| WY063 | Control | F | 51 | 160 | 56 | 19.44 | 97.13 | 87.59 | 71.47 | 1.20 | MIDDLE |
| WY147 | Drug | F | 53 | 162 | 59 | 11.90 | 71.49 | 55.02 | 2.64 | 1.14 | MIDDLE |
| WY204 | Control | F | 58 | 153 | 63 | 34.58 | 93.19 | 81.46 | 80.41 | 1.13 | MIDDLE |
| WY011 | Control | M | 50 | 169 | 65 | 26.01 | 61.55 | 50.72 | 28.51 | 1.02 | MIDDLE |
| WY139 | Control | M | 55 | 170 | 65 | 45.42 | 51.76 | 48.73 | -19.24 | 1.01 | MIDDLE |
| WY055 | Control | M | 41 | 176 | 85 | 26.95 | 88.26 | 72.36 | 35.18 | 1.00 | MIDDLE |
| WY175 | Drug | M | 58 | 166 | 65 | 11.92 | 95.96 | 77.80 | 48.53 | 0.99 | MIDDLE |
| WY198 | Drug | F | 51 | 155 | 54 | 37.76 | 98.43 | 70.26 | 53.46 | 0.90 | MIDDLE |
| WY044 | Drug | F | 59 | 165 | 66 | 34.70 | 99.17 | 83.32 | 39.86 | 0.88 | MIDDLE |
| WY129 | Drug | M | 40 | 171 | 80 | 28.63 | 94.79 | 75.06 | 68.07 | 0.85 | MIDDLE |
| WY121 | Control | F | 55 | 163 | 60 | 18.30 | 89.74 | 74.28 | 30.55 | 0.85 | MIDDLE |
| WY155 | Drug | F | 43 | 155 | 43 | 37.01 | 99.48 | 97.06 | 98.64 | 0.84 | MIDDLE |
| WY046 | Drug | F | 56 | 158 | 65 | 43.11 | 99.17 | 72.40 | 10.04 | 0.83 | MIDDLE |
| WY082 | Drug | F | 58 | 160 | 51 | 8.89 | 30.29 | 11.26 | -19.45 | 0.82 | MIDDLE |
| WY008 | Drug | M | 50 | 170 | 82 | 40.81 | 93.33 | 55.14 | 31.76 | 0.82 | MIDDLE |
| WY095 | Drug | M | 58 | 168 | 74 | 1.49 | 83.48 | 78.14 | 66.30 | 0.77 | MIDDLE |
| WY089 | Drug | F | 44 | 165 | 60 | 45.77 | 99.81 | 94.98 | 84.19 | 0.72 | MIDDLE |
| WY039 | Drug | F | 45 | 158 | 50 | 18.05 | 98.59 | 97.59 | 84.48 | 0.71 | MIDDLE |
| WY166 | Drug | F | 42 | 158 | 54 | 35.68 | 86.64 | 53.78 | 26.98 | 0.69 | MIDDLE |
| WY057 | Drug | M | 54 | 178 | 80 | 20.29 | 70.71 | 62.91 | 19.90 | 0.66 | MIDDLE |
| WY104 | Drug | F | 45 | 1.6 | 50 | 34.97 | 78.82 | 55.75 | 32.38 | 0.66 | MIDDLE |
| WY031 | Control | M | 54 | 169 | 85 | 20.31 | 63.55 | 58.64 | 25.59 | 0.62 | MIDDLE |
| WY007 | Drug | F | 47 | 168 | 70 | 20.00 | 48.68 | 66.06 | 23.31 | 0.62 | MIDDLE |
| WY021 | Drug | F | 44 | 159 | 54 | 32.37 | 89.07 | 90.57 | 60.15 | 0.61 | MIDDLE |
| WY030 | Control | M | 55 | 165 | 67 | 34.97 | 95.57 | 79.18 | 55.85 | 0.58 | MIDDLE |
| WY124 | Control | M | 45 | 177 | 72 | 35.74 | 97.93 | 98.71 | 92.72 | 0.58 | MIDDLE |
| WY090 | Control | M | 42 | 171 | 73 | 3.30 | 94.51 | 38.16 | 60.42 | 0.52 | MIDDLE |
| WY052 | Control | F | 54 | 154 | 60 | 42.44 | 95.58 | 77.37 | 35.25 | 0.52 | MIDDLE |
| WY064 | Drug | M | 50 | 165 | 68 | 26.44 | 80.31 | 21.88 | 16.04 | 0.51 | MIDDLE |
| WY069 | Drug | M | 50 | 165 | 60 | 3.69 | 95.56 | 94.73 | 83.54 | 0.51 | MIDDLE |
| WY187 | Drug | M | 53 | 160 | 59 | 43.05 | 90.00 | 71.38 | 57.85 | 0.50 | MIDDLE |
| WY199 | Control | F | 59 | 152 | 67 | 12.55 | 93.33 | 30.20 | 40.55 | 0.50 | MIDDLE |
| WY170 | Control | F | 47 | 156 | 55.5 | 38.49 | 99.36 | 96.58 | 81.86 | 0.48 | MIDDLE |
| WY133 | Control | F | 43 | 157 | 49 | 28.14 | 93.32 | 78.23 | 50.52 | 0.48 | MIDDLE |
| WY156 | Control | M | 46 | 160 | 55 | 42.19 | 98.57 | 95.59 | 75.61 | 0.47 | MIDDLE |
| WY116 | Drug | M | 47 | 172 | 63 | 34.12 | 96.82 | 75.19 | 37.84 | 0.47 | MIDDLE |
| WY091 | Control | F | 50 | 158 | 53 | 23.51 | 92.84 | 87.12 | 80.70 | 0.46 | MIDDLE |
| WY178 | Drug | F | 40 | 158 | 53 | 27.67 | 46.42 | 27.11 | -4.30 | 0.45 | MIDDLE |
| WY059 | Control | F | 43 | 166 | 56 | 10.58 | 83.82 | 56.73 | 30.62 | 0.45 | MIDDLE |
| WY149 | Control | M | 52 | 169 | 75 | 53.79 | 98.45 | 95.76 | 80.68 | 0.43 | MIDDLE |
| WY109 | Drug | F | 54 | 157 | 60 | 27.33 | 93.90 | 70.01 | 47.05 | 0.42 | MIDDLE |
| WY150 | Drug | F | 54 | 160 | 72 | 38.04 | 78.62 | 36.16 | 0.19 | 0.42 | MIDDLE |
| WY195 | Control | M | 40 | 164 | 61 | 31.26 | 71.09 | 58.48 | 42.03 | 0.40 | MIDDLE |
| WY160 | Drug | F | 49 | 165 | 55 | 18.96 | 98.73 | 85.29 | 52.34 | 0.40 | MIDDLE |
| WY169 | Drug | M | 41 | 164 | 72 | 16.58 | 82.06 | 69.99 | 54.33 | 0.38 | MIDDLE |
| WY111 | Control | F | 43 | 157 | 55 | 23.89 | 89.77 | 77.00 | 76.65 | 0.38 | MIDDLE |
| WY009 | Control | F | 51 | 153 | 47 | 17.08 | 96.12 | 86.42 | 53.52 | 0.37 | MIDDLE |
| WY203 | Control | F | 52 | 157 | 61 | 44.49 | 87.51 | 60.02 | 37.07 | 0.36 | MIDDLE |
| WY165 | Control | F | 43 | 155 | 60 | 46.93 | 96.82 | 82.22 | 51.32 | 0.36 | MIDDLE |
| WY114 | Control | M | 55 | 165 | 85.5 | 28.05 | 96.12 | 74.58 | 38.81 | 0.35 | MIDDLE |
| WY179 | Control | M | 56 | 180 | 100 | 34.26 | 80.26 | 75.70 | 48.95 | 0.33 | MIDDLE |
| WY163 | Drug | F | 49 | 158 | 57.5 | 27.74 | 78.88 | 55.56 | 25.96 | 0.32 | MIDDLE |
| WY032 | Control | M | 20 | 174 | 81 | 36.90 | 84.62 | 93.35 | 72.22 | 0.32 | MIDDLE |
| WY190 | Control | M | 54 | 169 | 64 | 30.65 | 64.54 | 77.15 | 68.64 | 0.31 | MIDDLE |
| WY115 | Drug | F | 41 | 154 | 67 | 18.84 | 93.65 | 69.98 | 55.74 | 0.30 | MIDDLE |
| WY168 | Drug | M | 42 | 172 | 67 | 29.25 | 73.66 | 97.34 | 76.35 | 0.29 | MIDDLE |
| WY132 | Drug | F | 58 | 153 | 52 | 23.80 | 89.74 | 54.20 | 2.70 | 0.28 | MIDDLE |
| WY085 | Control | F | 43 | 165 | 51 | 42.94 | 95.60 | 82.37 | 68.93 | 0.28 | MIDDLE |
| WY026 | Drug | F | 52 | 164 | 48 | 22.11 | 60.16 | 48.85 | 17.67 | 0.28 | MIDDLE |
| WY117 | Control | M | 51 | 179 | 72 | 50.39 | 66.92 | 71.48 | 33.93 | 0.27 | MIDDLE |
| WY062 | Drug | F | 49 | 153 | 58 | 5.90 | 87.19 | 71.20 | 61.97 | 0.26 | MIDDLE |
| WY086 | Drug | M | 52 | 172 | 92 | 32.67 | 97.36 | 90.61 | 74.33 | 0.26 | MIDDLE |
| WY140 | Control | F | 50 | 160 | 66 | 31.20 | 87.86 | 86.91 | 49.54 | 0.25 | MIDDLE |
| WY118 | Control | M | 45 | 171 | 74 | 47.44 | 78.95 | 47.17 | 24.61 | 0.25 | MIDDLE |
| WY083 | Control | F | 46 | 159 | 75 | 41.99 | 60.20 | 15.37 | 37.39 | 0.24 | MIDDLE |
| WY144 | Control | F | 55 | 160 | 61.5 | 61.83 | 98.50 | 86.85 | 63.23 | 0.24 | MIDDLE |
| WY130 | Control | M | 49 | 173 | 68 | 19.68 | 96.42 | 95.01 | 84.62 | 0.24 | MIDDLE |
| WY028 | Drug | M | 69 | 165 | 62 | 36.31 | 86.25 | 58.30 | 13.57 | 0.23 | MIDDLE |
| WY048 | Control | F | 54 | 161 | 55 | 41.71 | 95.03 | 87.18 | 68.64 | 0.23 | MIDDLE |
| WY181 | Control | M | 56 | 165 | 62 | 27.46 | 83.32 | 44.05 | 10.11 | 0.22 | MIDDLE |
| WY006 | Drug | F | 47 | 169 | 71 | 18.95 | 88.14 | 91.57 | 70.54 | 0.22 | MIDDLE |
| WY135 | Drug | M | 42 | 168 | 70 | 32.73 | 84.43 | 42.63 | 3.95 | 0.22 | MIDDLE |
| WY068 | Control | F | 58 | 160 | 46 | -0.47 | 92.40 | 60.80 | 32.95 | 0.21 | MIDDLE |
| WY087 | Drug | F | 45 | 160 | 52 | -1.86 | 98.06 | 98.47 | 89.60 | 0.21 | LOW |
| WY035 | Drug | F | 47 | 165 | 60 | 25.92 | 92.25 | 60.60 | 5.82 | 0.20 | LOW |
| WY017 | Drug | F | 45 | 166 | 56 | 8.07 | 98.75 | 91.28 | 59.04 | 0.20 | LOW |
| WY110 | Control | M | 57 | 162 | 61 | 27.74 | 75.26 | 55.17 | 28.83 | 0.20 | LOW |
| WY097 | Control | F | 46 | 165 | 75 | 13.98 | 77.76 | 71.36 | 36.08 | 0.19 | LOW |
| WY023 | Control | M | 53 | 169 | 71 | 17.00 | 76.01 | 47.21 | -2.34 | 0.18 | LOW |
| WY029 | Drug | M | 44 | 172 | 75 | 17.27 | 61.21 | 56.62 | 15.51 | 0.18 | LOW |
| WY202 | Drug | F | 56 | 155 | 55 | 18.28 | 80.05 | 34.26 | 9.19 | 0.18 | LOW |
| WY018 | Control | F | 52 | 160 | 57 | 6.63 | 66.30 | 66.08 | 35.68 | 0.17 | LOW |
| WY180 | Control | M | 58 | 165 | 58 | 19.22 | 41.65 | 37.93 | 4.97 | 0.17 | LOW |
| WY058 | Drug | F | 52 | 157 | 70 | 41.18 | 92.55 | 74.91 | 39.88 | 0.16 | LOW |
| WY071 | Control | M | 51 | 170 | 70 | 5.20 | 69.74 | 25.04 | -7.05 | 0.16 | LOW |
| WY013 | Control | M | 58 | 168 | 75 | 10.14 | 68.08 | 54.94 | 26.40 | 0.16 | LOW |
| WY164 | Drug | F | 59 | 165 | 68 | 35.64 | 97.33 | 94.98 | 76.68 | 0.15 | LOW |
| WY099 | Drug | M | 55 | 167 | 68 | -25.11 | 57.71 | 58.68 | 34.14 | 0.13 | LOW |
| WY105 | Control | F | 58 | 165 | 64 | 42.09 | 93.22 | 73.51 | 66.41 | 0.12 | LOW |
| WY128 | Control | F | 47 | 155 | 60 | 15.82 | 80.85 | 53.90 | 29.97 | 0.12 | LOW |
| WY146 | Drug | F | 43 | 155 | 60 | 43.16 | 89.96 | 58.13 | 19.46 | 0.12 | LOW |
| WY148 | Drug | M | 44 | 170 | 70 | 40.46 | 93.00 | 93.65 | 79.76 | 0.12 | LOW |
| WY201 | Control | F | 55 | 164 | 65 | 37.98 | 91.95 | 65.86 | 36.50 | 0.12 | LOW |
| WY012 | Drug | M | 48 | 170 | 60 | 39.38 | 88.99 | 74.36 | 39.56 | 0.12 | LOW |
| WY010 | Control | F | 51 | 158 | 48 | 29.93 | 89.77 | 61.55 | 26.56 | 0.11 | LOW |
| WY016 | Control | M | 47 | 171 | 76 | 19.05 | 97.33 | 94.43 | 66.72 | 0.11 | LOW |
| WY045 | Drug | F | 45 | 155 | 52 | 10.79 | 93.19 | 64.65 | 44.55 | 0.10 | LOW |
| WY101 | Drug | F | 43 | 170 | 60 | 29.20 | 80.69 | 67.30 | 55.32 | 0.10 | LOW |
| WY003 | Drug | F | 52 | 160 | 56 | 21.44 | 99.38 | 74.03 | 34.99 | 0.10 | LOW |
| WY131 | Control | F | 52 | 162 | 60 | 17.19 | 76.54 | 63.04 | 35.55 | 0.10 | LOW |
| WY077 | Control | F | 45 | 156 | 62 | 46.63 | 97.49 | 95.10 | 87.70 | 0.09 | LOW |
| WY093 | Drug | F | 52 | 165 | 67 | 23.20 | 91.26 | 72.49 | 55.73 | 0.08 | LOW |
| WY002 | Drug | F | 46 | 156 | 44 | 24.03 | 91.84 | 78.59 | 38.49 | 0.08 | LOW |
| WY103 | Control | M | 54 | 167 | 58 | 23.20 | 50.27 | 53.88 | -37.45 | 0.07 | LOW |
| WY138 | Control | M | 44 | 172 | 77 | 47.08 | 82.21 | 57.82 | -1.46 | 0.07 | LOW |
| WY072 | Drug | M | 59 | 174 | 76 | 30.18 | 98.73 | 77.77 | 48.21 | 0.07 | LOW |
| WY051 | Drug | M | 42 | 175 | 75 | 46.46 | 74.86 | 51.63 | -0.39 | 0.07 | LOW |
| WY200 | Control | M | 45 | 170 | 65 | 39.10 | 87.63 | 86.81 | 86.43 | 0.07 | LOW |
| WY122 | Control | F | 46 | 150 | 45 | 20.84 | 97.88 | 86.91 | 77.34 | 0.06 | LOW |
| WY027 | Drug | M | 51 | 171 | 98 | 37.43 | 89.93 | 53.30 | -1.68 | 0.06 | LOW |
| WY126 | Drug | M | 59 | 168 | 65 | 16.20 | 57.24 | 32.78 | 15.97 | 0.06 | LOW |
| WY098 | Drug | F | 48 | 159 | 55 | 12.08 | 90.79 | 79.09 | 42.72 | 0.06 | LOW |
| WY162 | Drug | F | 57 | 155 | 66.5 | 29.74 | 94.66 | 57.33 | 1.79 | 0.05 | LOW |
| WY066 | Control | M | 47 | 174 | 55 | 15.47 | 91.40 | 56.30 | -3.34 | 0.05 | LOW |
| WY107 | Drug | M | 59 | 175 | 75 | 33.11 | 89.33 | 74.09 | 59.57 | 0.05 | LOW |
| WY183 | Control | M | 46 | 165 | 70 | 24.99 | 73.54 | 56.51 | 40.64 | 0.05 | LOW |
| WY078 | Drug | M | 58 | 170 | 70 | 18.55 | 75.93 | 30.96 | -0.64 | 0.05 | LOW |
| WY113 | Control | M | 42 | 169 | 78 | 45.45 | 79.18 | 49.17 | 17.01 | 0.05 | LOW |
| WY171 | Control | M | 51 | 168 | 71 | 34.85 | 89.57 | 51.00 | 4.18 | 0.04 | LOW |
| WY106 | Drug | F | 47 | 162 | 60 | 44.15 | 83.23 | 49.05 | 23.63 | 0.04 | LOW |
| WY119 | Drug | M | 52 | 170 | 71 | 33.33 | 58.60 | 34.54 | 9.38 | 0.04 | LOW |
| WY120 | Drug | M | 41 | 170 | 75 | 28.01 | 81.25 | 69.61 | 31.29 | 0.04 | LOW |
| WY014 | Control | M | 53 | 170 | 77 | 38.57 | 72.18 | 58.09 | 21.28 | 0.04 | LOW |

Note: ^1^Control: vaccination alone; Drug: vaccination with GLSP intervention.

^2^HIGH/MIDDLE/LOW: the populations with high/moderate/low levels of baseline immunity.

**Table S2 The Statisticians of Percentage of Inhibition in Different Conditions**

| Group^1^ | Population of baseline immunity^2^ | Time point | Percentage of inhibition | | |
| --- | --- | --- | --- | --- | --- |
|  |  |  | Median | Mean | SD |
| Control | HIGH | Day 0 | 12.01 | 12.71 | 3.19 |
| Control | HIGH | Day 10 | 97.22 | 94.66 | 5.84 |
| Control | HIGH | Day 90 | 86.60 | 83.86 | 12.86 |
| Control | HIGH | Day 180 | 71.57 | 62.27 | 24.93 |
| Drug | HIGH | Day 0 | 12.45 | 12.61 | 2.44 |
| Drug | HIGH | Day 10 | 96.80 | 94.28 | 7.25 |
| Drug | HIGH | Day 90 | 80.79 | 79.50 | 11.57 |
| Drug | HIGH | Day 180 | 57.11 | 57.96 | 18.09 |
| Control | MIDDLE | Day 0 | 2.52 | 3.34 | 2.19 |
| Control | MIDDLE | Day 10 | 88.14 | 85.79 | 12.04 |
| **Control** | **MIDDLE** | **Day 90** | **69.98** | **67.67** | **19.26** |
| Control | MIDDLE | Day 180 | 40.55 | 44.71 | 26.11 |
| Drug | MIDDLE | Day 0 | 2.99 | 4.04 | 2.45 |
| Drug | MIDDLE | Day 10 | 94.01 | 87.48 | 15.18 |
| **Drug** | **MIDDLE** | **Day 90** | **77.97** | **74.82** | **20.17** |
| Drug | MIDDLE | Day 180 | 54.69 | 51.26 | 28.33 |
| Control | LOW | Day 0 | 0.51 | 0.53 | 0.26 |
| Control | LOW | Day 10 | 89.63 | 83.55 | 14.50 |
| Control | LOW | Day 90 | 65.26 | 64.51 | 18.85 |
| Control | LOW | Day 180 | 35.54 | 33.23 | 28.11 |
| Drug | LOW | Day 0 | 0.52 | 0.54 | 0.27 |
| Drug | LOW | Day 10 | 80.45 | 81.24 | 13.30 |
| Drug | LOW | Day 90 | 58.39 | 62.66 | 17.32 |
| Drug | LOW | Day 180 | 29.40 | 30.62 | 29.75 |

Note: ^1^Control: vaccination alone; Drug: vaccination with GLSP intervention.

^2^HIGH/MIDDLE/LOW: the populations with high/moderate/low levels of baseline immunity.

**Table S3 The Sample Information for Single-Cell Multi-Omics Sequencing**

| Number | Group* | Time points | scRNA-seq | scBCR-seq | scTCR-seq | scATAC-seq |
| --- | --- | --- | --- | --- | --- | --- |
|  |  |  |  |  |  |  |
| WY065 | Control | Day 0 | √ | √ | √ | √ |
| WY074 | Control | Day 0 | √ | √ | √ | √ |
| WY090 | Control | Day 0 | √ | √ | √ |  |
| WY177 | Control | Day 0 | √ | √ | √ | √ |
| WY179 | Control | Day 0 | √ | √ | √ |  |
| WY181 | Control | Day 0 | √ | √ | √ |  |
| WY065 | Control | Day 90 | √ | √ | √ |  |
| WY074 | Control | Day 90 | √ | √ | √ | √ |
| WY090 | Control | Day 90 | √ | √ | √ |  |
| WY177 | Control | Day 90 | √ | √ | √ | √ |
| WY179 | Control | Day 90 | √ | √ | √ | √ |
| WY181 | Control | Day 90 | √ | √ | √ |  |
| WY062 | Drug | Day 0 | √ | √ | √ |  |
| WY070 | Drug | Day 0 | √ | √ | √ |  |
| WY080 | Drug | Day 0 | √ | √ | √ |  |
| WY095 | Drug | Day 0 | √ |  | √ | √ |
| WY175 | Drug | Day 0 | √ | √ | √ | √ |
| WY198 | Drug | Day 0 | √ | √ | √ | √ |
| WY062 | Drug | Day 90 | √ | √ | √ | √ |
| WY070 | Drug | Day 90 | √ | √ | √ |  |
| WY080 | Drug | Day 90 | √ | √ | √ |  |
| WY095 | Drug | Day 90 | √ | √ | √ | √ |
| WY175 | Drug | Day 90 | √ | √ | √ |  |
| WY198 | Drug | Day 90 | √ | √ | √ | √ |

Note: *Control: vaccination alone; Drug: vaccination with GLSP intervention.

**Table S4 The Monitoring Ion Pairs, CV, and CE of Analytes**

| No. | Analyte | Precursor ion >Product ion | CV  (V) | CE  (V) |
| --- | --- | --- | --- | --- |
| 1 | GC2 | 517.3 > 287.2* | 48 | 40 |
|  |  | 517.3 > 151.1 | 48 | 40 |
| 2 | GC6 | 511.3 > 437.3 | 92 | 42 |
|  |  | 511.3 > 303.2* | 92 | 42 |
| 3 | GG | 513.3 > 265.1* | 92 | 34 |
|  |  | 513.3 > 301.2 | 92 | 34 |
| 4 | GB | 497.3 > 249.2* | 72 | 30 |
|  |  | 497.3 > 303.2 | 72 | 30 |
| 5 | GK | 555.3 > 265.1* | 60 | 40 |
|  |  | 555.3 > 469.3 | 60 | 40 |
| 6 | GA | 515.3 > 285.2* | 80 | 34 |
|  |  | 515.3 > 301.2 | 80 | 34 |
| 7 | GH | 553.4 > 437.4* | 92 | 46 |
|  |  | 553.4 > 467.5 | 92 | 32 |
| 8 | LA | 457.3 > 149.1* | 80 | 28 |
|  |  | 457.3 > 285.2 | 80 | 28 |
| 9 | GD2 | 511.3 > 263.1* | 80 | 30 |
|  |  | 511.3 > 299.2 | 80 | 30 |
| 10 | GED | 493.3 > 149.1* | 92 | 44 |
|  |  | 493.3 > 449.3 | 92 | 44 |
| 11 | GC1 | 495.3 > 149.1* | 82 | 40 |
|  |  | 495.3 > 301.2 | 82 | 40 |
| 12 | GF | 569.4 > 509.4* | 2 | 30 |
|  |  | 569.4 > 435.4 | 2 | 48 |

Note: *indicates the quantitative ion pair.

**Table S5 The Basic Information for the Molecular Docking of Target Proteins**

| Target Protein | PDB ID | Organisms | Agonist | PubChem CID of Agonists | Center Coordinates of Grid Boxes | | |
| --- | --- | --- | --- | --- | --- | --- | --- |
|  |  |  |  |  | x | y | z |
| *TLR7* | 5GMH | Macaca mulatta | R-848 | 159603 | -14.418 | -28.211 | -12.240 |
|  |  |  | CL097 | 11579618 |  |  |  |
|  |  |  | CL075 | 10198719 |  |  |  |
| *TLR8* | 3W3N | Homo sapiens | Same agonists as TLR7 | | 21.838 | 2.823 | 32.877 |
| *STING* | 4KSY | Homo sapiens | 2',3'-cGAMP | 136313972 | 47.008 | 5.242 | -17.871 |
|  |  |  | Cyclic di-GMP | 135440063 |  |  |  |
|  |  |  | MK-1454 | 137102568 |  |  |  |
| IFN-α2-*IFNAR2* complex | 3S9D | Homo sapiens | RO8191 | 2768133 | 32.481 | -19.837 | 63.912 |
| *IFNAR2* | Isolated from the IFN-α2-*IFNAR2* complex | Homo sapiens | Same agonist as the IFN-α2-*IFNAR2* complex | | 32.505 | -19.927 | 63.910 |

**Table S 6 The Collection Centers, Donors, and The Ethical Approval Information**

| Catalog Number | Lot Number | Donor ID | Gender | Age | Ethical Approval Scheme Number | Ethics Committee |
| --- | --- | --- | --- | --- | --- | --- |
| hPB010C | 059K111 | HZ251407 | Male | 21 | HYS-XSY-003 | Clinical Trials of Drugs, Ethics Committee, Zhejiang Xiaoshan Hospital, Hangzhou |
| hPB010C | 059K128 | HZ251621 | Male | 20 |  |  |
| hPB010C | 059K060 | 20240402MFY183 | Male | 26 |  |  |
| hPB010C | 059K114 | HZ251518 | Male | 22 |  |  |
| W-hPB010C | WEZ4172 | WPBZ1020 | Female | 21 | HYS-LQ-001 | Shanghai Liquan Hospital Institutional Ethics Committee |
| W-hPB010C | WEZ4333 | HW240578 | Female | 21 |  |  |
| W-hPB010C | WCZ2056 | C2208319591W | Female | 24 | HYS-KDF-001 | Medical Ethics Committee of Dr. Kang Healthcare (Zhejiang) Co., Ltd. |
| W-hPB010C | WCZ2109 | C220818309BW | Female | 23 |  |  |

**Table S 7 The Primer Sequences for qRT-PCR**

| Gene Name | Gene ID | Primer Sequences (5' to 3') |
| --- | --- | --- |
| *ACTB* | 60 | Forward: CACTCTTCCAGCCTTCCTTC  Reverse: GTACAGGTCTTTGCGGATGT |
| *MX1* | 4599 | Forward: GGCTGTTTACCAGACTCCGACA  Reverse: CACAAAGCCTGGCAGCTCTCTA |
| *OAS1* | 4938 | Forward: AGGAAAGGTGCTTCCGAGGTAG  Reverse: GGACTGAGGAAGACAACCAGGT |
| *ISG15* | 9636 | Forward: CTCTGAGCATCCTGGTGAGGAA  Reverse: AAGGTCAGCCAGAACAGGTCGT |
| *IFI44L* | 10964 | Forward: TGCACTGAGGCAGATGCTGCG  Reverse: TCATTGCGGCACACCAGTACAG |

**Table S8 Markers for Cell Type and Sub-type Annotation**

| Cell Types | Name of Cell Sub-types | | Markers |
| --- | --- | --- | --- |
| Innate immune cells (*PTPRC*^+^*CD19*^-^*CD3E*^-^[1]) | Natural killer cells (NK) | | *NCR1^+^*[2] |
|  | α-lymphoid progenitor cells (αLP) | | *IL7R^+^*[3] |
|  | Plasmacytoid dendritic cells (pDC) | | *IL3RA^+^* or *CLEC4C^+^*[4] |
|  | Hematopoietic stem cells (HSC) | | *CD34^+^*[4] |
|  | Myeloid (*CST3*^+^*LYZ*^+^[5]) | Classical monocytes (CM) | *CD14^+^FCGR3A^-^*[4] |
|  |  | Intermediate monocytes (IM) | *CD14^+^FCGR3A^+^*[4] |
|  |  | Non-classical monocytes (NM) | *CD14^-^FCGR3A^+^*[4] |
|  |  | Classical dendritic cells (cDC) | *CD1C^+^*[4] or *FLT3^+^*[6] |
|  |  | Platelet-monocyte aggregates (PMA) | *PF4^+^*[7] |
| B cells (*CD19*^+^[1]) | Naïve B cells (Bn) | | *IGHD^+^IGHM^+^CD24^+^*[8] |
|  | Immunoglobulin (Ig)-class unswitched memory B cells (Busm) | | *IGHD^+^IGHM^+^CD27^+^*[8] |
|  | Atypical memory B cells (Bam) | | *IGHD^+^CD27^-^ITGAX^+^*[8] |
|  | Ig-class switched memory B cells (Bsm) | | *IGHD^-^IGHM^-^CD27^+^*[8] |
|  | *CD3*^+^*CD19*^+^ dual-expressing cells (Bde) | | *CD3E^+^*[9] |
|  | *IgD*^-^*CD27*^-^ double-negative B cells (Bdn) | | *IGHD^-^CD27^-^*[10] |
|  | Platelet-B aggregates (PBA) | | *PF4*^+^[7] |
| T cells (*CD3D*^+^*CD3E*^+^[5]) | *CD4*^+^ central memory T cells (CD4 Tcm) | | *CD4*^+^*LEF1*^+^*CD27*^+^[5,11] |
|  | *CD4*^+^ regulatory T cells (CD4 Treg) | | *CD4*^+^*FOXP3*^+^[12] |
|  | *CD4*^+^ effector T cells (CD4 Te) | | *CD4*^+^*LEF1*^-^*CD27*^-^*GZMA*^+^[5,11] |
|  | *CD8*^+^ central memory T cells (CD8 Tcm) | | *CD8A*^+^*LEF1*^+^*CD27*^+^[5,11] |
|  | *CD8*^+^ effector memory T cells (CD8 Tem) | | *CD8A*^+^*LEF1*^-^CD27^+^*GZMA*^+^[5,11] |
|  | *CD8^+^* effector T cells (CD8 Te) | | *CD8A*^+^*LEF1*^-^*CD27*^-^*GZMA*^+^[5,11] |
|  | Natural killer T cells | | *CD4*^-^*CD8A*^-^*NCR1*^+^[13] |
|  | Mucosal-associated invariant T cells (MAIT) | | *SLC4A10*^+^*TRAV1−2*^+^[5] |
|  | Proliferating T cells (Tprolif) | | *MKI67*^+^[5] |
|  | γδ T cells (γδ T) | | *CD4*^-^*CD8A*^-^*TRDV2*^+^[5] |
|  | Platelet-T aggregates (PTA) | | *PF4*^+^[7] |
| Plasma | | | *CD19*^+^*MZB1*^+^[5] |
| Platelet | | | *PF4*^+^[7] |

**Table S9 The Compounds in GLSP Identified Using UPLC-QTOF-MS**

| No. | tR/min | Observed mass | Mass error/ppm | Formula | Identification | Major Fragments |
| --- | --- | --- | --- | --- | --- | --- |
|  |  |  |  |  |  |  |
|  |  |  |  |  |  |  |
| 1 | 3.97 | 533.3107 | -2.35 | C_30_H_46_O_8_ | Ganoderic acid L | 515.3007, 303.1593, 195.1052 |
| 2 | 4.14 | 529.2805 | -0.29 | C_30_H_42_O_8_ | (7beta,12alpha,24E)-7,12,28-Trihydroxy-3,11,15-trioxolanosta-8,24-dien-26-oic acid | 475.2963, 319.1874, 209.1164 |
| 3 | 4.72 | 527.2635 | -2.93 | C_30_H_40_O_8_ | Elfvingic acid B | 453.2277, 319.1533 |
| 4 | 4.8 | 513.2849 | -1.67 | C_30_H_42_O_7_ | (7b,15a,24Z)-7,15,23-trihydroxy-3,11-dioxolanosta-8,20,24-trien-26-oic acid isomer | 469.2957, 319.1922, 149.0973 |
| 5 | 5.77 | 531.2955 | -1.53 | C_30_H_44_O_8_ | Ganoderic acid I | 483.2376, 271.1339, 129.0554 |
| 6 | 6.54 | 515.3007 | -1.37 | C_30_H_44_O_7_ | Ganoderic acid DF | 441.2611, 249.1485, 73.0290 |
| 7 | 7.29 | 473.2547 | 0.41 | C_27_H_38_O_7_ | Lucidenic acid B isomer | 443.2060, 193.0863, 79.0547 |
| 8 | 7.29 | 515.3005 | -1.87 | C_30_H_44_O_7_ | Ganoderenic acid C | 443.2060, 303.1954, 79.0547 |
| 9 | 7.59 | 529.2795 | -2.21 | C_30_H_42_O_8_ | 20-hydroxyganoderic acid AM1 | 467.2788, 303.1582 |
| 10 | 7.73 | 513.2848 | -1.88 | C_30_H_42_O_7_ | Ganoderenic acid A isomer | 301.1801, 249.1494 |
| 11 | 8.02 | 475.2696 | -1.17 | C_27_H_40_O_7_ | Lucidenic acid C | 427.2111, 303.1949, 287.1638 |
| 12 | 8.32 | 571.2907 | -1.03 | C_32_H_44_O_9_ | 12β-acetoxy-7β-hydroxy-3,11,15,23-tetraoxo-5α-lanost-8-en-26-oic acid | 455.2471, 425.1956, 303.1572 |
| 13 | 8.42 | 517.3167 | -0.82 | C_30_H_46_O_7_ | Ganoderic acid C2 | 499.3057, 287.1648, 151.1122 |
| 14 | 8.79 | 527.2646 | -0.79 | C_30_H_40_O_8_ | Ganoderic acid AW1 isomer | 479.2048, 435.2155, 302.1509 |
| 15 | 9.01 | 531.2958 | -1.02 | C_30_H_44_O_8_ | 7β,15α,20-trihydroxy-3,11,23-trioxo-5α-lanosta-8-en-26-oic acid | 303.1953, 265.1435 |
| 16 | 9.2 | 529.2795 | -2.25 | C_30_H_42_O_8_ | (3β,7β,12β,20Z)-3,7,12-Trihydroxy-11,15,23-trioxolanosta-8,20(22)-dien-26-oic acid | 301.1809, 265.1436, 147.0811 |
| 17 | 10.1 | 511.2691 | -1.95 | C_30_H_40_O_7_ | Ganoderic acid C6 | 437.2328, 303.1592, 189.1276 |
| 18 | 11.05 | 511.2692 | -1.79 | C_30_H_40_O_7_ | Ganoderenic Acid D isomer | 481.2205, 300.1725, 263.1270 |
| 19 | 11.12 | 513.2853 | -0.85 | C_30_H_42_O_7_ | Ganoderic acid G | 301.1801, 207.1380, 149.0967 |
| 20 | 11.61 | 513.285 | -1.48 | C_30_H_42_O_7_ | Ganoderenic acid B | 495.2744, 303.1965, 147.0808 |
| 21 | 11.98 | 529.2793 | -2.59 | C_30_H_42_O_8_ | Ganoderic acid N | 399.2164, 129.0553 |
| 22 | 12.4 | 497.2906 | -0.43 | C_30_H_44_O_7_ | Ganoderic acid B | 494.2906, 303.1958, 149.0968 |
| 23 | 12.66 | 511.2696 | -1.01 | C_30_H_40_O_7_ | 23S-hydroxy-11,15-dioxo-ganoderic acid DM | 463.2100, 301.1797, 95.0492 |
| 24 | 13.28 | 513.2851 | -1.29 | C_30_H_42_O_7_ | Ganoderic acid AM1 isomer | 443.2068, 301.1797, 137.0958 |
| 25 | 13.64 | 571.2906 | -1.15 | C_32_H_44_O_9_ | 12β-acetoxy-3β,7β-dihydroxy-11,15,23-trioxo-lanost-8,16-dien-26-oic acid | 467.2792, 303.1958, 147.0811 |
| 26 | 14.03 | 513.2848 | -1.88 | C_30_H_42_O_7_ | Ganoderic acid LM2 | 301.1802, 193.0861, 79.0546 |
| 27 | 14.09 | 555.2691 | -0.4 | C_32_H_46_O_9_ | Ganoderic acid K | 513.2853, 493.2958, 193.0868, 79.0551 |
| 28 | 14.4 | 505.2798 | -1.81 | C_27_H_40_O_6_ | Chol-8-en-24-oic acid, 7,15-dihydroxy-4,4,14-trimethyl-3,11-dioxo-, (5alpha)- | 285.1485, 155.0707 |
| 29 | 14.61 | 527.2642 | -1.63 | C_30_H_40_O_8_ | Gibbosic acid C isomer | 317.1750, 101.0237 |
| 30 | 15.06 | 509.253 | -2.92 | C_30_H_40_O_8_ | Ganoderenic acid E | 447.2534, 299.1647, 101.0237 |
| 31 | 15.26 | 569.275 | -1.07 | C_32_H_42_O_9_ | Ganoleuconin F | 509.2529, 479.2071, 302.1519 |
| 32 | 15.44 | 569.2748 | -1.47 | C_32_H_42_O_9_ | 3beta-Hydroxy-12beta-acetoxyganodernoid D | 497.2158, 453.2282 |
| 33 | 15.51 | 515.3017 | 0.55 | C_30_H_44_O_7_ | Ganoderic acid A | 285.1491, 195.1019, 97.0652 |
| 34 | 15.79 | 553.2804 | -0.55 | C_32_H_44_O_9_ | Ganoderic acid H | 467.2800, 303.1594, 121.1015 |
| 35 | 15.97 | 501.3212 | -1.94 | C_30_H_46_O_6_ | Ganolucidic acid B | 421.2398, 287.2006 |
| 36 | 16.41 | 509.2538 | -1.35 | C_30_H_40_O_8_ | Deacetyl ganoderic acid F | 435.2172, 301.1431, 189.1279 |
| 37 | 16.78 | 511.2692 | -1.85 | C_30_H_42_O_8_ | Ganoderic acid D2 | 457.2585, 299.1646, 149.0600, 97.0652 |
| 38 | 16.79 | 457.2585 | -2.29 | C_27_H_38_O_6_ | Lucidenic acid A | 299.1647, 263.1280, 149.0600 |
| 39 | 17.45 | 515.2637 | -2.53 | C_29_H_40_O_8_ | Lucidenic acid E isomer | 455.2433, 425.1956 |
| 40 | 17.7 | 511.269 | -2.11 | C_30_H_40_O_7_ | Ganoderenic Acid D | 493.2579, 449.2686 |
| 41 | 18.68 | 495.2754 | 0.29 | C_30_H_40_O_6_ | Ganoderic acid C1 | 451.2855, 301.1802, 149.0602 |
| 42 | 19.65 | 513.2494 | -0.02 | C_29_H_38_O_8_ | Lucidenic acid D2 | 471.2384, 441.1920, 195.1025 |
| 43 | 19.97 | 511.2698 | -0.65 | C_30_H_40_O_7_ | Ganoderenic acid H isomer | 434.2457, 299.1647 |
| 44 | 20 | 553.2797 | -1.73 | C_32_H_44_O_9_ | Ganodernoid G | 299.1647, 285.1492, 97.0650 |
| 45 | 20.32 | 493.2591 | -0.99 | C_30_H_40_O_7_ | 3β-hydroxyganodernoid D isomer | 419.2207, 301.1807, 147.0810 |
| 46 | 20.63 | 499.3056 | -1.92 | C_30_H_44_O_6_ | Ganolucidic acid A | 437.3062, 285.1856, 149.0972 |
| 47 | 20.73 | 511.2689 | -2.31 | C_30_H_40_O_7_ | 3β-hydroxyganodernoid D | 419.2222, 299.1633 |
| 48 | 20.8 | 525.2468 | -4.89 | C_30_H_38_O_8_ | Ganoapplanoid E isomer | 461.1967, 315.1591 |
| 49 | 20.88 | 567.2588 | -2.11 | C_32_H_40_O_9_ | Ganodernoid D | 507.2369, 477.1915, 300.1355 |
| 50 | 21.33 | 551.2639 | -2 | C_32_H_42_O_9_ | Ganoderic acid F | 509.2534, 435.2173, 301.1433 |
| 51 | 21.59 | 513.2842 | -3.03 | C_30_H_42_O_7_ | Ganoderic acid J | 421.2372, 301.1799 |
| 52 | 22.84 | 533.3469 | -2.8 | C_31_H_50_O_7_ | methyl 4,23,29-trihydroxy-3,4-seco-olean-12-en-3-oate-28-oic acid isomer | 487.3408, 287.1636, 149.0610 |
| 53 | 23.4 | 499.3062 | -0.63 | C_30_H_44_O_6_ | Ganoderic acid GS-2 | 285.1477, 149.0597 |
| 54 | 23.59 | 485.3261 | -2.29 | C_30_H_46_O_5_ | Ganoderic acid XL3 | 375.2929, 289.2166 |
| 55 | 25.48 | 613.3003 | -2.52 | C_34_H_46_O_10_ | (4β,5β,6α,22R)-5-Hydroxy-1,26-dioxo-22,26-epoxyergosta-2,24-dien-4,6,27-triyl-triacetat | 509.2921, 479.2424, 345.1708 |
| 56 | 25.63 | 483.3111 | -0.94 | C_30_H_44_O_5_ | Ganolucidic acid E | 297.1515, 149.0448 |
| 57 | 26.78 | 527.3371 | -1.27 | C_32_H_48_O_6_ | (7alpha,22S,24E)-22-Acetoxy-7-hydroxy-3-oxolanosta-8,24-dien-26-oic acid | 485.3263, 289.2158 |
| 58 | 28.32 | 295.227 | -2.9 | C_18_H_32_O_3_ | 9-HODE | 281.2478, 79.9566 |
| 59 | 28.9 | 525.3204 | -3.29 | C_32_H_46_O_6_ | (22S,24E)-22-Acetoxy-3,7-dioxolanosta-8,24-dien-26-oic acid | 483.3109, 287.2012 |
| 60 | 29.46 | 517.3528 | -1.28 | C_30_H_48_O_4_ | Ganodermanontriol | 485.2769, 269.2103 |
| 61 | 31 | 467.3165 | -0.43 | C_30_H_44_O_4_ | Ganoderic acid DM | 423.3272, 325.1841 |
| 62 | 31.23 | 467.3165 | -0.36 | C_30_H_44_O_4_ | Ganoderic acid TR | 325.1841, 255.1747, |
| 63 | 33.25 | 271.2268 | -3.94 | C_16_H_32_O_3_ | Hexadecaneperoxoic acid | 227.2009, 83.0495 |

**Table S10 The Concentrations of Triterpenoids in GLSP Obtained by UPLC-MRM-MS**

| NO. | Triterpenoid | Concentration (%) |
| --- | --- | --- |
| 1 | Ganoderic acid C2 | 0.021 |
| 2 | Ganoderic acid C6 | 0.034 |
| 3 | Ganoderic acid G | 0.048 |
| 4 | Ganoderic acid B | 0.043 |
| 5 | Ganoderic acid K | 0.021 |
| 6 | Ganoderic acid A | 0.130 |
| 7 | Ganoderic acid H | 0.078 |
| 8 | Lucidenic acid A | 0.011 |
| 9 | Ganoderic acid D2 | 0.025 |
| 10 | Ganoderenic aid D | 0.050 |
| 11 | Ganoderic acid C1 | 0.073 |
| 12 | Ganoderic acid F | 0.080 |
| Total | | 0.614 |

**References**

[1] Mitsialis V, Wall S, Liu P, Ordovas-Montanes J, Parmet T, Vukovic M, et al. Single-Cell Analyses of Colon and Blood Reveal Distinct Immune Cell Signatures of Ulcerative Colitis and Crohn’s Disease. Gastroenterology 2020;159:591-608.e10. https://doi.org/10.1053/j.gastro.2020.04.074.

[2] Mandelboim O, Lieberman N, Lev M, Paul L, Arnon TI, Bushkin Y, et al. Recognition of haemagglutinins on virus-infected cells by NKp46 activates lysis by human NK cells. Nature 2001;409:1055–60. https://doi.org/10.1038/35059110.

[3] Ghaedi M, Takei F. Innate lymphoid cell development. J Allergy Clin Immunol 2021;147:1549–60. https://doi.org/10.1016/j.jaci.2021.03.009.

[4] Villani A-C, Satija R, Reynolds G, Sarkizova S, Shekhar K, Fletcher J, et al. Single-cell RNA-seq reveals new types of human blood dendritic cells, monocytes, and progenitors. Science 2017;356:eaah4573. https://doi.org/10.1126/science.aah4573.

[5] Ren X, Wen W, Fan X, Hou W, Su B, Cai P, et al. COVID-19 immune features revealed by a large-scale single-cell transcriptome atlas. Cell 2021;184:1895-1913.e19. https://doi.org/10.1016/j.cell.2021.01.053.

[6] Liu K, Nussenzweig MC. Origin and development of dendritic cells. Immunological Reviews 2010;234:45–54. https://doi.org/10.1111/j.0105-2896.2009.00879.x.

[7] Wang H, Liu C, Xie X, Niu M, Wang Y, Cheng X, et al. Multi-omics blood atlas reveals unique features of immune and platelet responses to SARS-CoV-2 Omicron breakthrough infection. Immunity 2023;56:1410-1428.e8. https://doi.org/10.1016/j.immuni.2023.05.007.

[8] Glass DR, Tsai AG, Oliveria JP, Hartmann FJ, Kimmey SC, Calderon AA, et al. An Integrated Multi-omic Single-Cell Atlas of Human B Cell Identity. Immunity 2020;53:217-232.e5. https://doi.org/10.1016/j.immuni.2020.06.013.

[9] Ahmed R, Omidian Z, Giwa A, Cornwell B, Majety N, Bell DR, et al. A Public BCR Present in a Unique Dual-Receptor-Expressing Lymphocyte from Type 1 Diabetes Patients Encodes a Potent T Cell Autoantigen. Cell 2019;177:1583-1599.e16. https://doi.org/10.1016/j.cell.2019.05.007.

[10] Beckers L, Somers V, Fraussen J. IgD-CD27- double negative (DN) B cells: Origins and functions in health and disease. Immunol Lett 2023;255:67–76. https://doi.org/10.1016/j.imlet.2023.03.003.

[11] Borst J, Hendriks J, Xiao Y. CD27 and CD70 in T cell and B cell activation. Curr Opin Immunol 2005;17:275–81. https://doi.org/10.1016/j.coi.2005.04.004.

[12] Scheinecker C, Göschl L, Bonelli M. Treg cells in health and autoimmune diseases: New insights from single cell analysis. J Autoimmun 2020;110:102376. https://doi.org/10.1016/j.jaut.2019.102376.

[13] Yu J, Mitsui T, Wei M, Mao H, Butchar JP, Shah MV, et al. NKp46 identifies an NKT cell subset susceptible to leukemic transformation in mouse and human. J Clin Invest 2011;121:1456–70. https://doi.org/10.1172/JCI43242.
